# Homochiral Emissive Λ_8_‐ and Δ_8_‐[Ir_8_Pd_4_]^16+^ Supramolecular Cages

**DOI:** 10.1002/chem.201703273

**Published:** 2017-09-12

**Authors:** Diego Rota Martir, Daniel Escudero, Denis Jacquemin, David B. Cordes, Alexandra M. Z. Slawin, Herbert A. Fruchtl, Stuart L. Warriner, Eli Zysman‐Colman

**Affiliations:** ^1^ Organic Semiconductor Centre EaStCHEM School of Chemistry University of St Andrews St Andrews, Fife KY16 9ST UK; ^2^ CEISAM UMR CNRS 6230 Université de Nantes 2 rue de la Houssinière, BP 92208 44322 Nantes Cedex 3 France; ^3^ Institut Universitaire de France 1, rue Descartes 75005 Paris Cedex 5 France; ^4^ School of chemistry University of Leeds Woodhouse Lane Leeds LS2 9JT UK

**Keywords:** cage compounds, iridium, palladium, photochemistry, self-assembly

## Abstract

Synthetic self‐assembly is a powerful technique for the bottom‐up construction of discrete and well‐defined polyhedral nanostructures resembling the spherical shape of large biological systems. In recent years, numerous Archimedean‐shaped coordination cages have been reported based on the assembly of bent monodentate organic ligands containing two or more distal pyridyl rings and square‐planar Pd^II^ ions. The formation of photoactive Pd^II^ metallamacrocycles and cages, however, remain rare. Here we report the first examples of emissive and homochiral supramolecular cages of the form [Ir_8_Pd_4_]^16+^. These cages provide a suitably sized cavity to host large guest molecules. Importantly, encapsulation and energy transfer have been observed between the blue‐emitting NBu_4_[Ir(dFppy)_2_(CN)_2_] guest and the red‐emitting Δ_8_‐[Ir_8_Pd_4_]^16+^ cage.

## Introduction

Chemists have often found inspiration from the spontaneous and precise self‐assembly exhibited by biological systems into giant, well‐defined and functional superstructures.^1^ In natural photosynthesis, for instance, organisms optimise solar energy conversion through self‐organised assemblies of photofunctional chromophores.^2^ Similarly, catalysis is possible as a consequence of the secondary and tertiary structures of self‐assembled proteins, providing well‐defined local environments to mediate biochemical transformations.^3^ Much effort has been devoted to the preparation of large artificial nanostructures to mimic the precise assembly of multiple protein subunits into giant, polyhedral functional structures.^4^ Nowadays, the self‐assembly between square‐planar palladium(II) or platinum(II) metal ions and complementary bent ligands containing specifically positioned distal pyridine moieties, first demonstrated by Fujita et al.,^5^ is one of the most popular and successful strategies to prepare molecular capsules or cages.4b, 6 These nanostructures generally possess well‐defined internal cavities that promote the ingress of guest molecules and have been exploited in sensing,^7^ gas storage and purification,^8^ and catalysis.^9^ Usually the metal ions play solely a structural role within these supramolecular architectures; however, more recently, there has been increasing interest in the investigation of photophysically active supramolecular architectures. These have included systems that incorporate photophysically active metal ions as structural units within the architecture frameworks,^10^ as well as those that employ ligand scaffolds decorated with photoactive units, including luminescent metal complexes.^11^ Photoactive cages and metallamacrocycles provide restricted shape and size to govern host–guest interactions and, as a consequence of the optoelectronic communication between host and guest, distinct photophysical properties that are difficult to attain in conventional molecular materials can be achieved.^12^

Iridium(III) complexes possess a highly desirable set of optoelectronic and physical properties, including colour tunability across the visible spectrum, high photoluminescence quantum yields with short phosphorescence lifetimes and high chemical stability.^13^ They have been used as integral components of sensors,^14^ as luminescent biological probes^15^ and as emitters in electroluminescent devices.^13^, ^16^ However, despite their desirable photophysical properties, there exist to date only a handful of examples of photoactive iridium(III) complexes in supramolecular architectures, including cages,^17^ coordination capsules and metallamacrocycles,9b, 10a, 18 coordination polymers and MOFs,^19^ discrete paddlewheel structures,^20^ and soft salts.^21^ These metallosupramolecular assemblies generally show red‐shifted emission compared to their mononuclear analogues and, except for the coordination capsule reported by Lusby et al.,10a decreased photoluminescence quantum yields, *Φ*_PL_, and shorter emission lifetimes, *τ*_e_.

Here we report the first examples of homochiral red‐emitting supramolecular cages of the form of [Ir_8_Pd_4_]^16+^ that are able to encapsulate large anionic guests, including a blue‐emitting NBu_4_[Ir(dFppy)_2_(CN)_2_] complex. Controlled photoinduced energy transfer from the donor anionic iridium complex guest to the acceptor iridium metalloligands in the [Ir_8_Pd_4_]^16+^ cage is efficiently promoted. Such photoactive homochiral assemblies have the potential to mediate enantioselective photocatalytic reactions and act as single white‐light emissive materials.

## Results and Discussion

**Figure 1:**
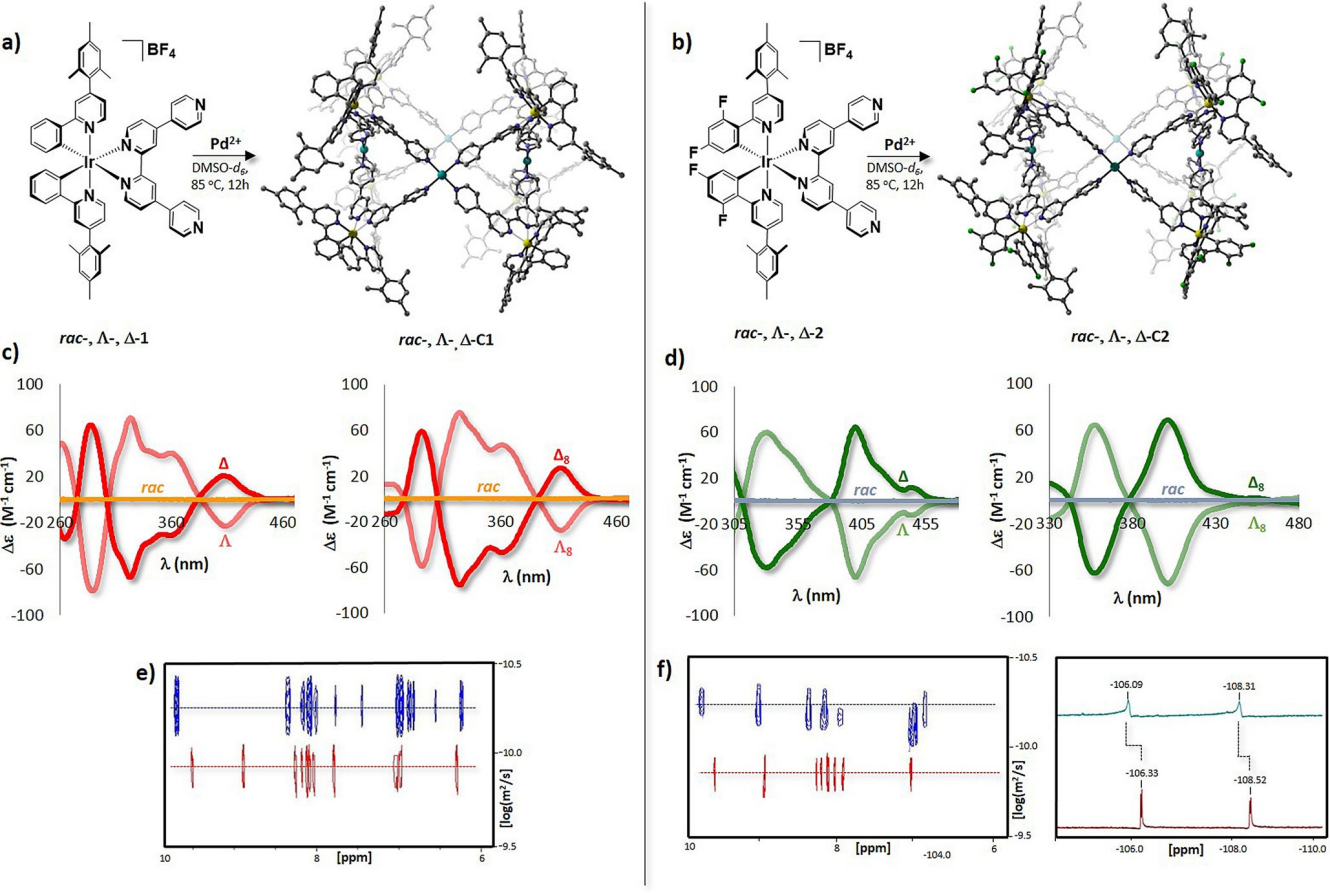
Self‐assembly between the metalloligands, and Pd^2+^ ions yielding: a) for *rac*‐**1**, Λ‐**1**, Δ‐**1**; racemic *rac*‐**C1**, and homochiral Λ_8_‐**C1** and Δ_8_‐**C1** cages, respectively (for clarity, only the calculated structure of Λ‐**C1** obtained from Λ‐**1** is shown) and b) for *rac*‐**2**, Λ‐**2,** Δ‐**2**; *rac*‐**C2**, Λ_8_‐**C2** and Δ_8_‐**C2** cages (for clarity, only the calculated structure of Λ‐**C2** obtained from Λ‐**2** is shown). c) CD spectra collected in CH_2_Cl_2_ at 298 K; light‐red lines: Λ‐**1** (left), Λ‐**C1** (right); red lines: Δ‐**1** (left) and Δ‐**C1** (right); orange lines: *rac*‐**1** (left) and *rac*‐**C1** (right). d) CD spectra collected in CH_2_Cl_2_ at 298 K; light‐green lines: Λ‐**2** (left), Λ‐**C2** (right); green lines: Δ‐**2** (left) and Δ‐**C2** (right); light‐blue lines: *rac*‐**2** (left) and *rac*‐**C2** (right). The CD spectra of **1** and **2** were collected at a concentration of 5×10^−5^ m while the concentration of **C1** and **C2** was maintained at 1×10^−5^ M. e) ^1^H DOSY NMR of Δ‐**1**, in red and Δ‐**C1**, in blue. f) ^1^H DOSY NMR of Δ‐**2**, in red and Δ‐**C2**, in blue (left) and stacked ^19^F NMR spectra of Δ‐**2** in red, and Δ‐**C2** in blue (right). The geometries of Λ‐**C1** and Λ‐**C2** have each been determined in vacuo at the HF/6‐31G(d) level of theory.

We report herein the first example of phosphorescent cages based on the self‐assembly between two families of Ir^III^ metalloligands of the form [Ir(C^N)_2_(qpy)]BF_4_ [where C^N is mesppy=2‐phenyl‐4‐mesitylpyridinato and dFmesppy=2‐(4,6‐difluorophenyl)‐4‐mesitylpyridinato, and qpy is 4,4′:2′,2′′:4′′,4′′′‐quaterpyridine] with Pd^2+^ ions through N_py_−Pd coordination (Figure 1 a, b). Each family of metalloligands is easily accessed in racemic form in a five step synthesis;^22^ however, with the aim of assessing the impact of iridium‐based chirality on the self‐assembly, we also prepared the enantiopure metalloligands Λ‐ and Δ‐[Ir(mesppy)_2_(qpy)]BF_4_ (Λ‐**1** and Δ‐**1**) and Λ‐ and Δ‐[Ir(dFmesppy)_2_(qpy)]BF_4_ (Λ‐**2** and Δ‐**2**) by using l‐ and d‐serine as chiral auxiliaries, following the protocol illustrated in Scheme S1 in the Supporting Information.^23^ The absolute configuration for each of the enantiopure iridium dimers Λ,Λ‐**D1**, Δ,Δ‐**D1**, Λ,Λ‐**D2** and Δ,Δ‐**D2** (Scheme S1) has been unambiguously determined by X‐ray crystallography (Figure S59 in the Supporting Information) and used to ascertain the absolute configurations of the enantiomers Λ‐**1**, Δ‐**1**, Λ‐**2** and Δ‐**2**.^23^ The enantiomeric excess of the bulk samples was confirmed by CD spectroscopy (Figure 1 c, d). We introduced the bulky mesityl substituent at the 4‐position of the pyridine of C^N ligands of **1** and **2** to increase the solubility of the complexes in organic solvents and to reduce intramolecular interactions without interfering in the assembly process.^24^ The presence of fluorine atoms in **2** provides a useful tag for monitoring both the self‐assembly process and the purity of the cage by ^19^F NMR spectroscopy and, by virtue of their electron‐withdrawing nature, for promoting a blue‐shift in the absorption and emission spectra concomitant with a stabilisation of the HOMO of the complex.^25^

When any of the metalloligands *rac*‐**1**, Λ‐**1**, Δ‐**1** and *rac*‐**2**, Λ‐**2**, Δ‐**2** and [Pd(NCMe)_4_][BF_4_]_2_ were heated in a 2:1 ratio in [D_6_]dimethyl sulfoxide ([D_6_]DMSO) at 85 °C for 12 h, the proton resonances associated with the metalloligand broadened and experienced downfield shifts (Figures S23 and S28 in the Supporting Information). The broad ^1^H NMR signals are indicative of the formation of very large assemblies, the tumbling motion of which is very slow on the NMR timescale.^26^ As expected, the proton resonances associated with the proton in *ortho*‐position to the distal nitrogen of the qpy moiety (H^a^, H^b^, in Figures S23 and S28) were most sensitive to the axial coordination of the pyridine ring to Pd. Evidence for the formation of a single species was confirmed by ^1^H DOSY NMR spectroscopy with a single diffusion coefficient (*D*) in [D_6_]DMSO of 5.2×10^−11^ m^2^ s^−1^ and 4.9×10^−11^ m^2^ s^−1^, respectively, for **C1** and **C2** (Figure 1 e, f, and Figures S26 and S30). These diffusion coefficients are indicative of much larger structures than either of the two metalloligands **1** and **2**, which show nearly identical diffusion coefficients in [D_6_]DMSO of 1.3×10^−10^ m^2^ s^−1^ and 1.2×10^−10^ m^2^ s^−1^, respectively (Figure 1 e, f and Figures S27 and S31). The corresponding hydrodynamic radii (*r*_s_) of **C1** and **C2** are calculated to be 19.8 Å and 20.0 Å, respectively (Table S1 in the Supporting Information). ^19^F NMR spectroscopy further confirmed quantitative conversion from **2** to **C2**, with the fluorine resonances associated with the dFppy ligand shifted downfield from −106.33 ppm and −108.52 ppm in **2** to −106.09 ppm and −108.31 ppm in **C2** (Figure 1 f and Figure S29 in the Supporting Information). As the two doublets associated with the fluorine resonances of the dFppy ligands in **2** are maintained in the ^19^F NMR spectra of **C2**, the local *C*_2_ symmetry present around the iridium centre in **2** is maintained also in the cage. Furthermore, no differences in the ^1^H, ^1^H DOSY and ^19^F NMR spectra were observed when the enantiopure metalloligands Λ‐**1** and Δ‐**1** or Λ‐**2** and Δ‐**2** were employed towards the self‐assembly of the cages in lieu of the racemic analogues *rac*‐**1** and *rac*‐**2** (Figures S25–S29 in the Supporting Information).

The compositions of the assemblies **C1** and **C2** have been unequivocally established to be [**1**_8_Pd_4_][BF_4_]_16_ and [**2**_8_Pd_4_][BF_4_]_16_, respectively, by HR‐ESI‐MS spectrometry, showing isotopically resolved peaks for [**C1**‐(BF_4_)_*n*_]^*n*+^ (*n*=4–8). For example, each of the ESI‐MS spectra of *rac*‐, Λ‐**C1** and Δ‐**C1** reveal peaks at *m*/*z=*2635.5650, 2067.2391, 1707.8640, 1451.5934 and 1259.2675, which are assigned to [**C1**‐(BF_4_)_4_]^4+^, [**C1**‐(BF_4_)_5_]^5+^, [**C1**‐(BF_4_)_6_]^6+^, [**C1**‐(BF_4_)_7_]^7+^ and [**C1**‐(BF_4_)_8_]^8+^, respectively (Figure S33 in the Supporting Information). Similarly, the charge states [**C2**‐(BF_4_)_*n*_]^*n*+^ (*n*=5–8), were likewise observed in the MS spectra of each of *rac*‐, Λ‐**C2** and Δ‐**C2** at *m*/*z=*2067.2391, 1707.8640, 1451.5934 and 1259.2765, respectively (Figure S38). The isotopically resolved distributions of these spectra closely match the simulated spectra. The ESI‐MS spectra of all the cages can be found in Figures S33–S41 in the Supporting Information. Among supramolecular assemblies composed of ligands containing two or more pyridine units possessing divergent vectors and Pd^2+^ ions, the stoichiometry [(L)_8_Pd_4_] is rare as this relative stoichiometry is only possible when the angle between the coordinating 4‐pyridyl units is inferior to 90°.^26^

The CD spectra of Λ‐**C1**, Δ‐**C1**, Λ‐**C2** and Δ‐**C2** revealed that the Ir‐centred stereochemistry of the eight metalloligands was maintained during the self‐assembly, and homochiral cages of compositions Λ_8_‐ and Δ_8_‐[**1**_8_Pd_4_][BF_4_]_16_ and Λ_8_‐ and Δ_8_‐[**2**_8_Pd_4_][BF_4_]_16_ were formed (Figure 1 c, d). When *rac*‐**1** and *rac*‐**2** were employed as the metalloligands, racemic mixtures of composition *rac*‐[**1**_8_Pd_4_][BF_4_]_16_ and *rac*‐[**2**_8_Pd_4_][BF_4_]_16_, respectively, were formed (orange and light‐blue lines in Figure 1 c, d), although this did not enable us to determine if these complexes were racemic cages, or racemic mixtures of enantiopure cages.

**Figure 2:**
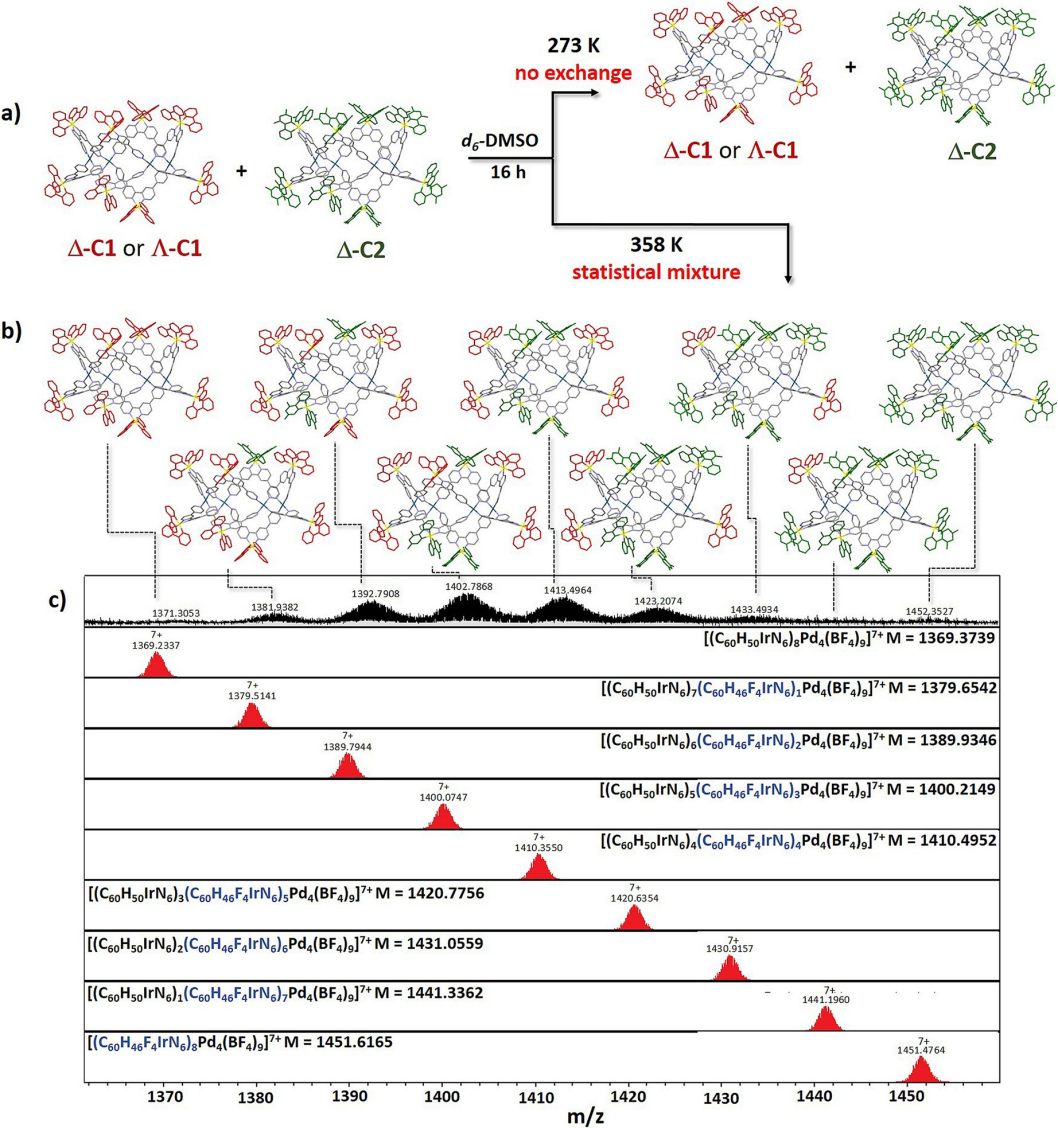
a) Schematic representation of the exchange experiments carried out by mixing Δ‐**C1** or Λ‐**C1** (ppy ligand in red) with Δ‐**C2** (dFppy ligand in green). The mesityl substituents have been omitted for clarity. b) Illustration of the formation of statistical mixture of cage species of compositions [(Λ‐**/**Δ‐**1**)_*n*_(Δ‐**2**)_*m*_Pd_4_][BF_4_]_16_ (*n*+*m*=8, from Λ‐**/**Δ‐**1**:Δ‐**2**=7:1 to Λ‐**/**Δ‐**1**:Δ‐**2**=1:7). c) ESI mass spectra of the 7+ charge states [(**1**)_*n*_(**2)**_*m*_(BF_4_)_7_]^7+^ (*n*+*m*=8, from **1:2**=7:1 to **1:2**=1:7) of Δ_8_‐**C1**‐BF_4_+Δ_8_‐**C2**‐BF_4_ heated at 85 °C for 12 h. In red are illustrated the simulated 7+ charge states of the heteroleptic cages. The same statistical distributions are observed for the 8+, 6+ and 5+ charge states.

In order to ascertain the impact of the nature of the iridium‐centred stereochemistry on the assembly of the cages, we examined the self‐assembly, in [D_6_]DMSO, of Pd^2+^ ions with one equivalent of one of the isostructural and enantiopure metalloligands, Λ‐**1** or Δ‐**1**, and one equivalent of Δ‐**2**. There are three possibilities by which similarly shaped components can self‐assemble in structures: 1) random mixing,^27^ 2) well‐defined mixing,^28^ or 3) self‐sorting.^29^ ESI‐MS of a [D_6_]DMSO solution containing either Λ‐**1** or Δ‐**1** with Δ‐**2** and [Pd(NCMe)_4_][BF_4_]_2_ stirred at 85 °C for 12 h show a statistical mixture of cage species of composition [(Λ‐**1/**Δ‐**1**)_*n*_(Δ‐**2**)_*m*_Pd_4_][BF_4_]_16_ (*n*+*m*=8, from Λ‐**1**/Δ‐**1**:Δ‐**2**=7:1 to Λ‐**1**/Δ‐**1**:Δ‐**2**=1:7, Figures S42 and S43 in the Supporting Information), indicating that our cages do not assemble by self‐sorting with respect to either the chirality or identity of the metalloligands. Similarly, mixing the pre‐formed cages Λ‐**C1** and Δ‐**C1** with Δ‐**C2** (Figure 2 a) at 85 °C for 12 h resulted in a rapid exchange between ligands Λ‐**1**, Δ‐**1,** and Δ‐**2** (Figure 2 b, cand Figures S44 and S45). As illustrated in Figures S45 a, c, the isotopically resolved distributions of the 7+ charge states, [(Λ‐**C1**/Δ‐**C1**)_*n*_(Δ‐**C2)**_*m*_(BF_4_)_7_]^7+^ closely match the simulated spectra. When homochiral cages of the same stereochemistry, Δ‐**C1** and Δ‐**C2**, are mixed at 85 °C in [D_6_]DMSO, the formation of homochiral heteronuclear cages are observed by CD spectroscopy with a CD spectrum intermediate for the mixed cage assemblies (Figure S45 h, dark‐blue line). However, when homochiral cage Λ‐**C1** is mixed with Δ‐**C2** at 85 °C, which is of opposite stereochemistry, the formation of racemic heteronuclear cages is promoted (Figure S45 h, light‐blue line). This was expected considering that the chirality of the iridium core does not contribute directly to the overall self‐assembly process. No metalloligand exchange is observed when either homochiral cage Λ‐**C1**/Δ‐**C1** is mixed with Δ‐**C2** at room temperature, and the cages show a high degree of kinetic inertness (Figures S45 e–S47).

The structures of both **C1** and **C2** were modelled at the HF/6‐31G(d) level of theory (Figure 1 a), and were found to be very similar. They resemble metallamacrocyclic structures in which two ligands doubly bridge between adjacent Pd centres around the macrocycle, in a crown‐like fashion.^30^ Of potential structures of [L_8_Pd_4_] cages, this particular arrangement is rare, only five examples of assemblies with this structural motif were reported to date.^30^, ^31^ The calculated structure confirmed that the qpy vector of the metalloligands **1** and **2** is compatible to form the [Ir_8_Pd_4_]^16+^ cages identified by mass spectrometry (Figure S32 in the Supporting Information). The optimised cage exhibits a diameter of approximately 18.8 Å (corresponding to the Pd⋅⋅⋅Pd distance), an internal volume from the top to the bottom bounds of the structure of approximately 3480 Å^3^, and a distance between neighbouring Ir atoms bridging the same Pd⋅⋅⋅Pd edge of approximately 13.7 Å. The radius around the metallamacrocyclic core across long axes of the structure, measures 21.5 Å (Figure S32) and matches with the hydrodynamic radii obtained by ^1^H NMR DOSY analysis (*r*_s_=19.8 Å). The cage structure can be seen to be approximately *C*_4_ symmetric about the Pd_4_ square. Unfortunately, while single crystals of cages **C1** or **C2** could be grown, and were examined by X‐ray diffraction, all crystals investigated showed extremely weak diffraction, with even synchrotron radiation not showing diffraction above 1.6 Å. Attempted structure solutions have given the positions of the metal cations and poorly ordered parts of the ligands, the data not, as yet, being amenable to refinement (Figure S62).

**Figure 3:**
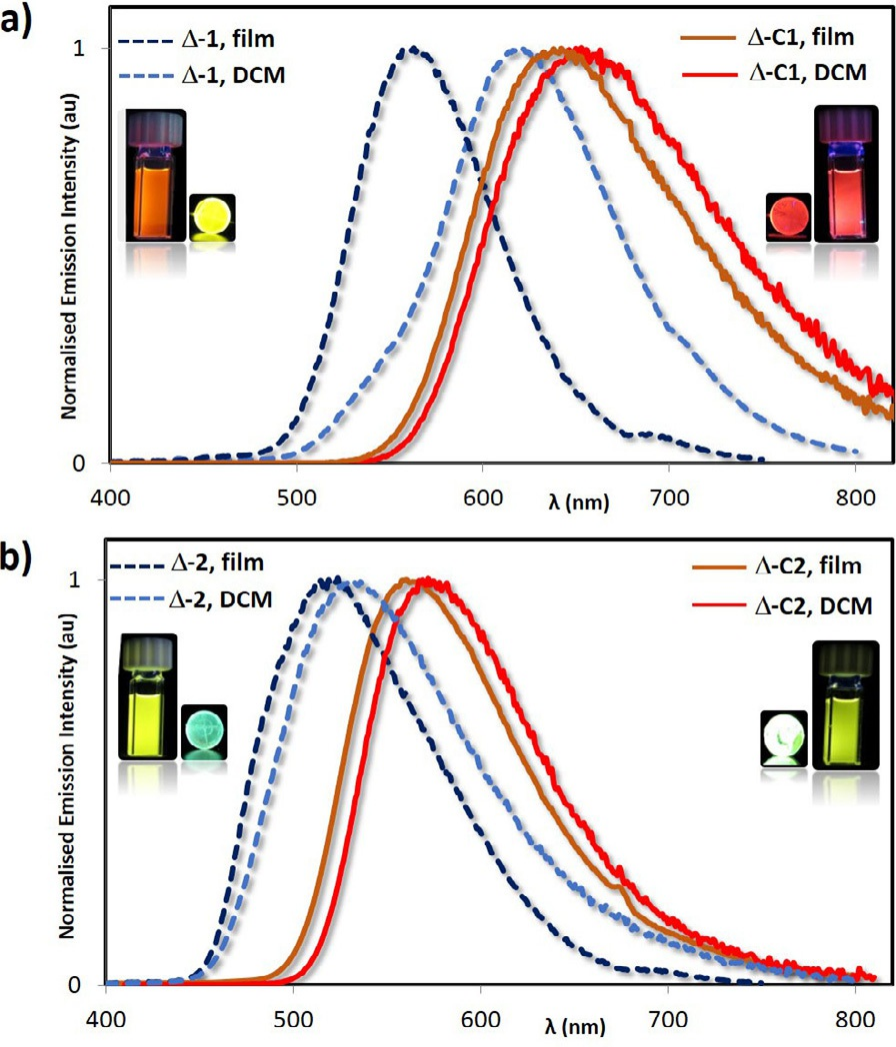
Normalised emission spectra of: a) Δ‐**1** and Δ‐**C1** and b) Δ‐**2** and Δ‐**C2**. Dotted dark‐blue lines: PMMA‐doped film with 5 wt % of metalloligands Δ‐**1** and Δ‐**2** spin‐coated on quartz substrates; Dotted light‐blue lines: deaerated CH_2_Cl_2_ solution of Δ‐**1** and Δ‐**2**; Solid orange lines: PMMA‐doped film with 5 wt % of cages Δ‐**C1** and Δ‐**C2** spin‐coated on quartz substrates; Solid red lines: deaerated CH_2_Cl_2_ solution of Δ‐**C1** and Δ‐**C2**.

In CH_2_Cl_2_, the photophysical properties of the racemic metalloligands *rac*‐**1** and *rac*‐**2** and of the racemic cages *rac*‐**C1** and *rac*‐**C2** are identical to those of the respective homochiral analogues Λ‐**1**/Δ‐**1**, Λ‐**2**/Δ‐**2**, Λ‐**C1**/Δ‐**C1**, and Λ‐**C2**/Δ‐**C2** (Table 1). The emission profiles of both families of cages **C1** and **C2** in CH_2_Cl_2_ are red‐shifted, respectively, at 655 nm and 561 nm, relative to those of the corresponding metalloligands **1** (*λ*_max_=620 nm) and **2** (*λ*_max_=527 nm). Their photoluminescence quantum yields, *Φ*_PL_, and emission lifetimes, *τ*_e_, are correspondingly lower and shorter, respectively (e.g., *rac*‐**C1**: *Φ*_PL_=5 %, *τ*_e_=202 ns; *rac*‐**C2**: *Φ*_PL_=10 %, *τ*_e_=825 ns), compared to those of **1** and **2** (e.g., *rac*‐**1**: *Φ*_PL_=14 %, *τ*_e_=300 ns; *rac*‐**2**: *Φ*_PL_=34 %, *τ*_e_=1000 ns).^22^, ^32^ These features are reflected in the excited‐state decay kinetics. Indeed, both families of homochiral and racemic coordination cages **C1** and **C2** exhibit slightly smaller radiative rate constants (*k*_r_, e.g., 2.47×10^5^ s^−1^ for *rac*‐**C1** and 1.21×10^5^ s^−1^ for *rac*‐**C2**), and slightly larger non‐radiative rate constants (*k*_nr_, e.g., 4.66×10^6^ s^−1^ for *rac*‐**C1** and 1.09×10^6^ s^−1^ for *rac*‐**C2**), relative to the corresponding metalloligands (e.g., *rac*‐**1**: *k*_r_ **=**4.67×10^5^ s^−1^, *k*_nr_ **=**2.45×10^6^ s^−1^; *rac*‐**2**: *k*_r_ **=**3.40×10^5^ s^−1^, *k*_nr_ **=**6.60×10^5^ s^−1^). The presence of the electron‐withdrawing fluorine atoms in **C2** induces a blue‐shift in the emission relative to the fluorine‐free cage **C1**. Similar to that observed for **1** and **2**, the emission profiles of **C1** and **C2** are broad and unstructured, an indication that the nature of the emission remains unchanged and is from mixed metal‐to‐ligand and ligand‐to‐ligand charge transfer (^3^MLCT/^3^LLCT) states (Figure 3 and Figures S64 and 65 in the Supporting Information).^22^ The red‐shifted emissions of the cages **C1** and **C2** in both CH_2_Cl_2_ and PMMA‐doped films (PMMA=poly(methyl methacrylate)) relative to those of the corresponding metalloligands can be interpreted as the result of coordination of the Lewis acidic Pd^II^ to the iridium complex. By acting as a Lewis acid, the Pd^II^ ions lower the LUMO levels of complexes **1** and **2** located on the qpy ligand,^22^ giving rise to smaller optical gaps.

**Table 1:** Photophysical properties of **1**, **C1**, **2** and **C2**.

		*λ*_em_ [nm]			*Φ*_PL_ [%]			*τ*_e_ [ns]	
		CH_2_Cl_2_^[a]^	film^[b]^		CH_2_Cl_2_^[c]^	film^[b,d]^		CH_2_Cl_2_^[a]^	film^[b,e]^
*rac*‐**1**		620	564		14	28.0		300	344 (0.14), 1045 (0.86)
Δ‐**1**		620	563		13	28.7		300	333 (0.13), 1038 (0.87)
Λ‐**1**		620	563		13	26.4		299	343 (0.12), 1044 (0.88)
*rac*‐**C1**		655	643		5	10.9		204	49 (0.12), 270 (0.68), 715 (0.20)
Δ‐**C1**		655	643		5	10.3		202	47 (0.12), 269 (0.67), 707 (0.21)
Λ‐**C1**		655	643		5	9.6		202	48 (0.12), 266 (0.67), 695 (0.21)
*rac*‐**2**		565	518		34	41.2		1000	48 (0.09), 259 (0.21), 1195 (0.70)
Δ‐**2**		564	518		35	42.3		1001	46 (0.08), 246 (0.22), 1184 (0.70)
Λ‐**2**		565	519		31	40.9		1001	48 (0.08), 240 (0.22), 1189 (0.70)
*rac*‐**C2**		573	531		10	16.8		825	13 (0.14), 412 (0.17), 1125 (0.69)
Δ‐**C2**		572	531		8	16.3		824	13 (0.14), 378 (0.14), 1101 (0.72)
Λ‐**C2**		573	531		11	15.8		824	11 (0.13), 372 (0.14), 1117 (0.73)

[a] Measurements in degassed CH_2_Cl_2_ at 298 K (*λ*_ex_=360 nm). [b] Thin films formed by spin‐coating on a pristine quartz substrate. [c] *Φ*_PL_ measurements were carried out in degassed CH_2_Cl_2_ under nitrogen (*λ*_exc_=360 nm) using quinine sulfate as the external reference (*Φ*_PL_=54.6 % in 0.5 m H_2_SO_4_ at 298 K).^33^ [d] Values obtained using an integrating sphere. [e] Values in parentheses are pre‐exponential weighting factor, in relative % intensity, of the emission decay kinetics (*λ*_exc_=378 nm).

In order to mitigate non‐radiative vibrational motion, we spin‐coated 5 wt % of **1**, **2**, **C1** and **C2** in PMMA, which serves as an inert matrix. As a result of the less polar environment and the rigidification conferred by the PMMA host, the emissions of **1**, **2**, **C1** and **C2** in the thin films were blue‐shifted, respectively at 564 nm, 518 nm, 643 nm and 531 nm (Figure 3, Figures S64 and 65 in the Supporting Information), with enhanced *Φ*_PL_ and longer multi‐exponential *τ*_e_ (as representative examples: *rac*‐**1**: *Φ*_PL_=28 %, *τ*_e_=344, 1045 ns, *rac*‐**2**: *Φ*_PL_=41 %, *τ*_e_=48, 259, 1195 ns, *rac*‐**C1**: *Φ*_PL_=11 %, *τ*_e_=49, 270, 715 ns, and *rac*‐**C2**: *Φ*_PL_=17 %, *τ*_e_=13, 412, 1125 ns) relative to the photophysical behaviour in CH_2_Cl_2_ (Table 1).

The calculated cage structures, *rac*‐**C1**, Λ‐**C1**, Δ‐**C1** all show an internal pocket volume of approximately 3480 Å^3^, which is sufficient to include large guest molecules, including mononuclear phosphorescent iridium complexes. Several studies have demonstrated that the photophysical properties of luminescent transition‐metal complexes emitting from CT states strongly depend on the local environment.18b, 34 For example, Umakoshi et al.18b encapsulated an Ir^III^ complex, [Ir(ppy)_2_(bpy)]Cl (bpy is 2,2′‐bipyridine), within a hexameric resorcinarene hydrogen‐bonded capsule and observed that the capsule effectively hampers the non‐radiative decay thereby enhancing both the *Φ*_PL_ and the *τ*_e_ of the encapsulated iridium guest. We targeted the encapsulation of blue‐emitting Ir^III^ guests within the confined cavity of our red‐emitting cage Δ‐**C1** to study the nature of the energy‐transfer process between donor guest and acceptor host cage. Importantly, by modulating the degree of energy transfer between the donor and the acceptor as a function of the choice of medium or concentration, emission of white light can also be achieved.21a, 35 In the context of iridium phosphors, this approach has been investigated in multi‐metallic covalently linked complexes,^36^ soft salts,21a or in MOFs containing emissive materials,19d but still remains unexplored in photo‐active host–guest assemblies.^35^

**Figure 4:**
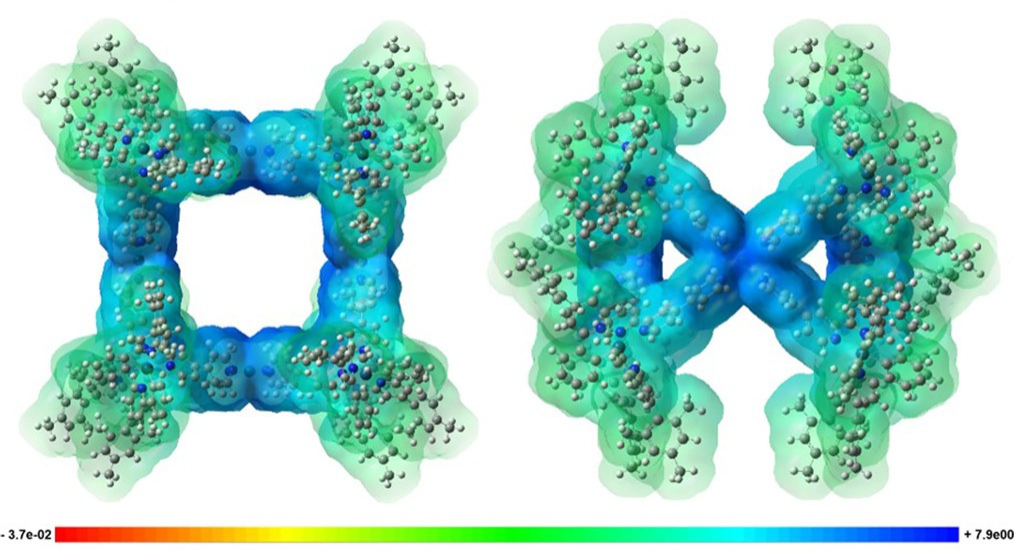
Molecular electrostatic potential [HF/6‐31G(d)] map of Δ‐**C1** with front (left) and central (right) views. The most positive potential regions are shown in deep blue.

**Figure 5:**
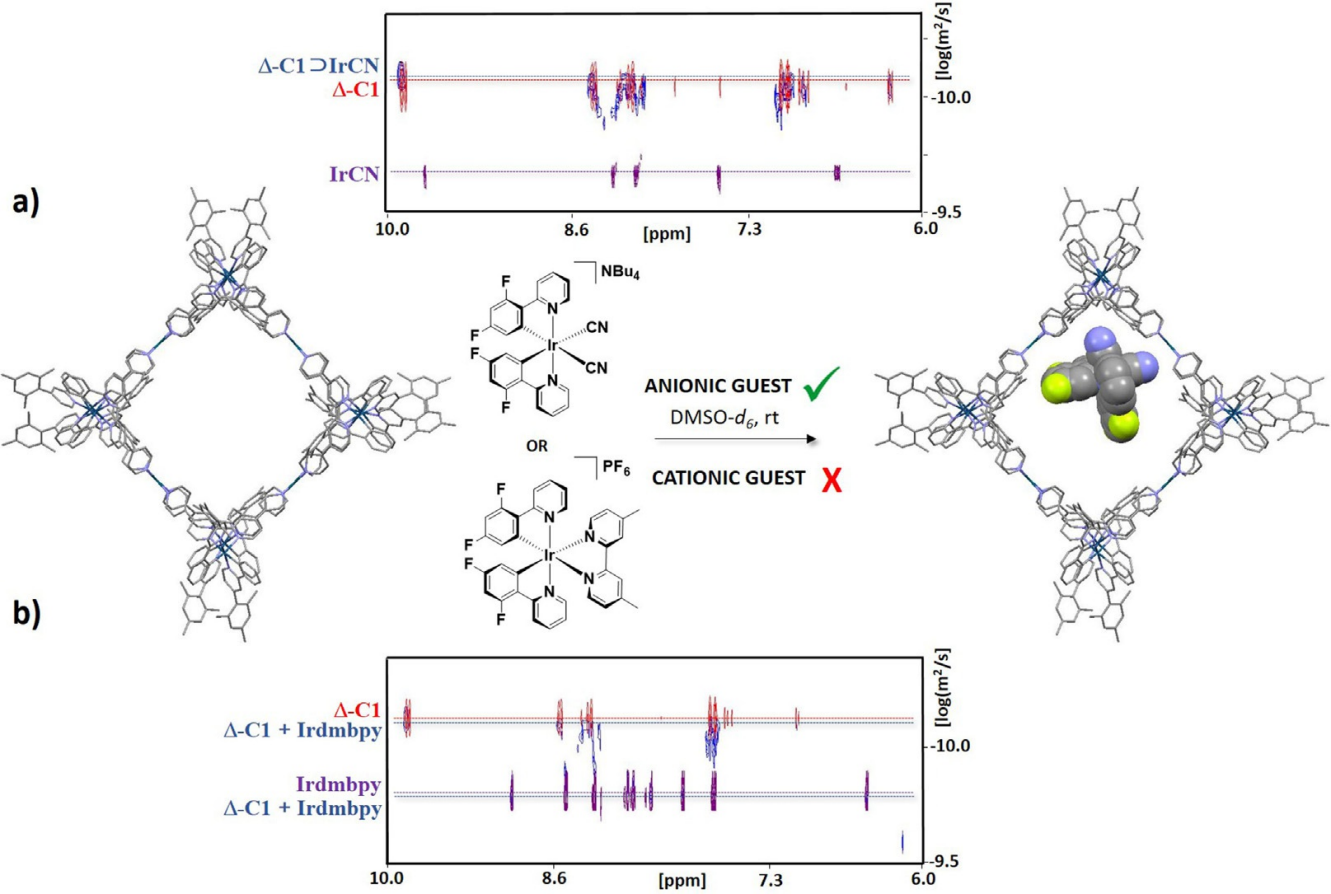
**a)** Representation of encapsulation in [D_6_]DMSO at room temperature of complex the NBu_4_[Ir(dFppy)_2_(CN)_2_] (**IrCN**) within the cavity of cage Δ‐**C1**; including (top) the ^1^H DOSY NMR spectra of **IrCN** (in purple), Δ‐**C1** (in red) and a 1:1 solution of **IrCN** and Δ‐**C1** (Δ‐**C1**⊃**IrCN**, in blue). The HF/6‐31G(d) optimised host–guest structure Δ‐**C1⊃IrCN** is shown. b) ^1^H DOSY NMR spectra of **Irdmbpy** (in purple), Δ‐**C1** (in red) and a 1:1 solution of **Irdmbpy** and Δ‐**C1** (Δ‐**C1** + **Irdmbpy**, in blue).

Preliminary ^1^H NMR studies on the interactions between a selected range of small organic guest compounds and Δ‐**C1** revealed that the cage interacts selectively with anionic guests in [D_6_]DMSO. Interactions can be observed with ammonium tetraphenyl borate or ammonium pyrenecarboxylate (see ^1^H NMR spectra in Figures S48 c and S49), but no interaction is observed for neutral guest compounds, such as pyrene or pyrene carboxylic acid (see ^1^H NMR spectra in Figure S48 a,b in the Supporting Information). A similar behaviour was observed for the interaction of guest molecules with a polycationic [Pd_2_L_4_]^12+^ cage (in whihch L are acridinium panels connected by a *meta*‐phenylene spacers).18b Indeed, in the computed molecular electrostatic potential map of Δ‐**C1** (Figure 4), all regions are of positive potential, the most positive potential regions of Δ‐**C1** being found in the pocket of the cage (in the closest proximity to the Pd^II^ ions). Therefore, favourable interactions are only expected with negatively charged guests. We next turned our attention to investigate the interactions between the blue‐emitting anionic [Ir(dFppy)_2_(CN)_2_]^−^complex (**IrCN**)^37^ with cage Δ‐**C1** (Figure 5 a). ^1^H DOSY NMR analysis of a room‐temperature solution containing one equivalent of both Δ‐**C1** and **IrCN** in [D_6_]DMSO revealed a significant reduction of the diffusion coefficient of **IrCN** (*D*_(**IrCN)**_=1.9×10^−10^ m^2^ s^−1^) after mixing with cage Δ‐**C1** to form the host–guest system Δ‐**C1**⊃**IrCN** (*D*_(Δ‐**C1**⊃**IrCN)**_=4.9–6.0×10^−11^ m^2^ s^−1^, Figure 5 a), with a diffusion coefficient similar to that of host Δ‐**C1** (*D*_(Δ‐**C1)**_=5.3×10^−11^ m^2^ s^−1^). In addition, the ^1^H NMR spectra of Δ‐**C1**⊃**IrCN** revealed that the binding of **IrCN** with cage Δ‐**C1** proceeds with significant broadening of the resonances associated with **IrCN** (Figure S50), confirming its slow tumbling motion on the NMR timescale. A downfield shift and a significant broadening of the fluorine resonances of **IrCN** in Δ‐**C1**⊃**IrCN** were also observed by ^19^F NMR spectroscopy (Figure S52). In contrast, when the cationic complex [Ir(dFppy)_2_(dmbpy)]PF_6_ (dmbpy=4,4′‐dimethyl‐2,2′‐bipyridine; **Irdmbpy**) was mixed with Δ‐**C1** in [D_6_]DMSO at room temperature, no binding affinity was observed by ^1^H, ^19^F and ^1^H DOSY NMR spectroscopy (Figure 5 b and Figures S55–S57).

The potential host–guest complexes, Δ‐**C1**⊃**IrCN** and Δ‐**C1**⊃**Irdmbpy**, were optimised at the HF/6‐31G(d) level of theory in order to gain insights into the nature of the host–guest interactions. For the optimised Δ‐**C1**⊃**IrCN** host–guest structure, the **IrCN** complex is located in the pocket of the cage (Figure 5 a), in agreement with the electrostatic potential map predictions for the cage. Its optimised structure reveals weak interactions between the cyano ligand of **IrCN** with one of the Pd^2+^ ions (3.2 Å) and several C−H units of Δ‐**C1**. By contrast, any attempts to optimise a Δ‐**C1**⊃**Irdmbpy** host–guest structure did not lead to a stable complex. Indeed, both units fall apart during the optimisation process, stressing that no favourable interactions between **Irdmbpy** and Δ‐**C1** could be found, and that this holds both in the pocket and on the exterior surface of the cage.

**Figure 6:**
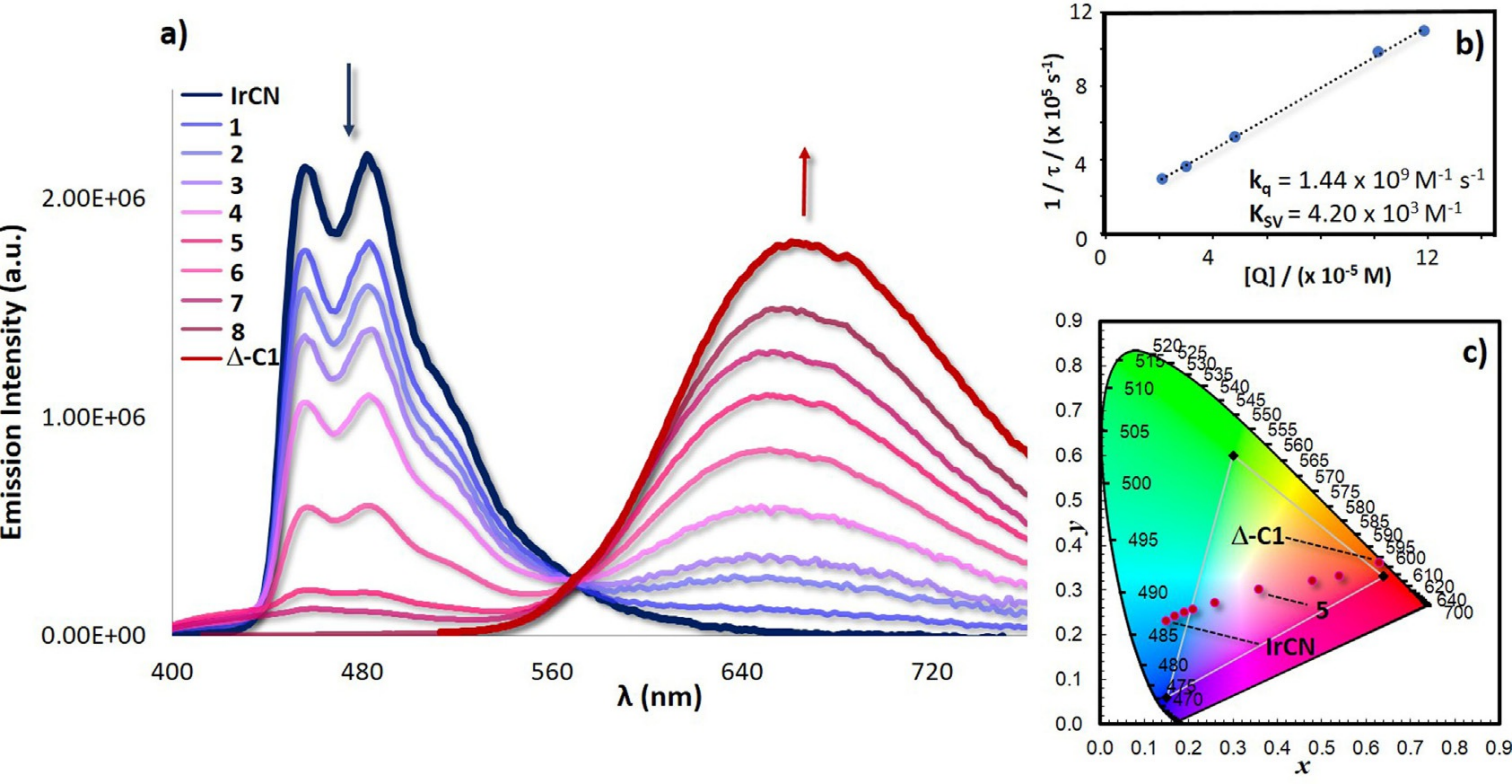
a) Emission titrations of Δ‐**C1** (0 μm: **IrCN**; 2 μm: 1; 5 μm: 2; 10 μm: 3; 21 μm: 4; 30 μm: 5; 48 μm: 6; 78 μm: 7; 101 μm: 7; 120 μm: 8) into a 100 μm solution of **IrCN** at 298 K in degassed DMSO. b) Stern–Volmer plot of the quenching study between **IrCN** and Δ‐**C1**. The emission lifetimes of **IrCN** were monitored at 480 nm (*τ*_e_^o^=2915 ns) upon photoexcitation at 378 nm. c) CIE diagram indicating the change of emission colours during the emission titration.

The anionic complex **IrCN** exhibits a blue ^3^LC emission in DMSO, with two maxima at 458 and 483 nm and a shoulder at 515 nm (blue line in Figure 6 a), a *Φ*_PL_ of 52 %, and a *τ*_e_ of 2915 ns. The same vibronic emission profile, with *λ*_max_ at 460 nm and 485 nm, was observed in CH_2_Cl_2_, but with a higher *Φ*_PL_ of 80 % and a longer *τ*_e_ of 3280 ns.^37^ Emission titration of cage Δ‐**C1** (from 0 to 120 μm) into a 100 μm degassed solution of **IrCN** in DMSO at 298 K results in a gradual quenching of the blue emission of the donor **IrCN** together with a gradual enhancement of the emission of the red‐emitting cage at 666 nm with an isosbestic point observed at 565 nm (Figure 6 a). At a concentration of 110 μm of Δ‐**C1** (titration 8 in Figure 6 a), the emission of the **IrCN** was completely quenched and only emission from Δ‐**C1** was observed. Upon photoexcitation of Δ‐**C1**⊃**IrCN** at 360 nm, energy transfer from the blue‐emitting **IrCN** to the red‐emitting Δ‐**C1** is therefore promoted. This emission titration data could be fitted to a 1:1 binding model (Figure S79 in the Supporting Information) with a binding constant *K*_b_ of 3.9×10^6^±0.2 m ^−1^ for the formation of Δ‐**C1**⊃**IrCN** from Δ‐**C1** and **IrCN**. This association constant is in the range reported for encapsulation of anionic guests into polycationic host cages.^38^

To study the energy transfer between anionic **IrCN** and Δ‐**C1** in Δ‐**C1**⊃**IrCN**, Stern–Volmer quenching analysis was carried out (Table S4 in the Supporting Information).^32^ Based on a bimolecular quenching model, the reciprocal of the lifetime of **IrCN** is linearly correlated to the concentration of the quencher Δ‐**C1** (Figure 6 b). From this analysis, we calculated a quenching rate constant (*k*_q_) of 1.44×10^9^ m ^−1^ s^−1^ and a Stern–Volmer constant (K_SV_) of 4.20×10^3^ m ^−1^, suggesting that the energy‐transfer/quenching process in Δ‐**C1**⊃**IrCN** is very efficient.21a, 39 Förster energy transfer is unlikely to be an efficient pathway for energy transfer due to the poor spectral overlap between the absorption of Δ‐**C1** and the emission of **IrCN** (Figure S81), therefore, Dexter energy transfer is the likely mechanism for the energy transfer in system.21a The CIE (Comminsion Internationale de l′Éclairage) diagram shown in Figure 6 c illustrates the change in the emission colours observed during the emission titration. Titration 5 (Figure 6 a) shows CIE coordinates of (0.36, 0.30), which are close to coordinates of the pure white light (*x*: 0.31, *y*: 0.33). By contrast, emission titrations of Δ‐**C1** (from 0 to 120 μm) into a 100 μm solution of the cationic **Irdmbpy** complex at 298 K in DMSO did not show any evidence of quenching of the emission of **Irdmbpy** to the Δ‐**C1** cage (Figure S90); rather a superposition of the emission spectra of the two species was observed. These findings are consistent with our computational investigation of Δ‐**C1**⊃**Irdmbpy**, which did not lead to a stable complex. Both emission studies and host–guest simulations demonstrate that high binding affinity between the host and the guest is required to promote energy transfer.

## Conclusion

Emissive and homochiral supramolecular Pd_4_L_8_ cages have been prepared by self‐assembly between Pd^2+^ ions and two families of enantiopure metalloligands, Λ‐ and Δ‐[Ir(mesppy)_2_(qpy)]BF_4_ and Λ‐ and Δ‐[Ir(dFmesppy)_2_(qpy)]BF_4_. The polycationic cage Λ‐**C1** selectively encapsulates anionic compounds. Strong binding and efficient energy transfer (*k*_q_=1.44×10^9^ m ^−1^ s^−1^) between the anionic blue‐emitting complex [Ir(dFppy)_2_(CN)_2_]^−^ and the red‐emitting cage Λ‐**C1** has been observed. Examples of efficient energy transfer between luminescent guests and photoactive cages are rare. These cages are promising candidates as chiral photoactive containers capable of absorbing photons and transferring light energy to or from encapsulated guest acceptors. These assemblies open up the possibility of promoting stereoselective photocatalytic transformations, examples of which at present are exceedingly rare. On the materials front, the host–guest assemblies can serve as stable white‐light emitting materials for solution‐processed electroluminescent devices.

## Conflict of interest

The authors declare no conflict of interest.

## Supporting information

As a service to our authors and readers, this journal provides supporting information supplied by the authors. Such materials are peer reviewed and may be re‐organized for online delivery, but are not copy‐edited or typeset. Technical support issues arising from supporting information (other than missing files) should be addressed to the authors.

Supplementary
