# Supplementary material for: Homochiral Emissive Λ8‐ and Δ8‐[Ir8Pd4]16+ Supramolecular Cages
Source: Chemistry. 2017 Sep 12;23(57):14358–66. doi: 10.1002/chem.201703273 (PMC5656816; doi:10.1002/chem.201703273)
Supplement: Supplementary file 1 — Supplementary [file CHEM-23-14358-s001.pdf]

CHEMISTRY
A European Journal

Supporting Information

# Homochiral Emissive $\Lambda_8$- and $\Delta_8$-$[Ir_8Pd_4]^{16+}$ Supramolecular Cages

Diego Rota Martir,[a] Daniel Escudero,[b] Denis Jacquemin,[b, c] David B. Cordes,[a]
Alexandra M. Z. Slawin,[a] Herbert A. Fruchtl,[a] Stuart L. Warriner,[d] and Eli Zysman-Colman*[a]

chem_201703273_sm_miscellaneous_information.pdf

# Table of Contents:


| | Pages |
|---|---|
| Experimental section | **S3** |
| Characterization of $[Ir(C^\wedge N)_2(Serine)]PF_6$ complexes | **S17** |
| Characterization of $[Ir(C^\wedge N)_2(N^\wedge N)]PF_6$ complexes | **S18** |
| NMR characterization of **$\Lambda_8$, $\Delta_8$-C1**, **$\Lambda_8$,$\Delta_8$-C2** | **S29** |
| Calculation of hydrodynamic radii ($r_s$) of **$\Lambda_8$, $\Delta_8$-C1**, **$\Lambda_8$,$\Delta_8$-C2** | **S34** |
| ESI-MS characterization of **$\Lambda_8$, $\Delta_8$-C1**, **$\Lambda_8$,$\Delta_8$-C2** | **S35** |
| Formation of heteronuclear cages by mixing **Λ, Δ-1**, **Δ-2** and $Pd^{2+}$ | **S40** |
| Formation of heteronuclear cages by mixing **$\Lambda_8$, $\Delta_8$-C1**, **$\Delta_8$-C2** | **S42** |
| Host-guest assemblies monitored by NMR spectroscopies | **S45** |
| Crystal structures of **Λ-, Λ-** and **Δ-, Δ-D2** and ***rac*-2** | **S52** |
| Optelectronic characterization | **S56** |
| Energy transfer between **IrCN** and **$\Delta_8$-C1** | **S64** |
| No energy transfer between **Irdmbpy** and **$\Delta_8$-C1** | **S70** |
| Computational details | **S72** |
| References | **S72** |

# Experimental section.

*General Synthetic Procedures*. Commercial chemicals were used as supplied. All reactions in the synthesis of the metalloligands and metallocages were performed using standard Schlenk techniques under inert ($N_2$) atmosphere with reagent-grade solvents. Flash column chromatography was performed using silica gel (Silia-P from Silicycle, 60 Å, 40-63 μm). Analytical thin layer chromatography (TLC) was performed with silica plates with aluminum backings (250 μm with indicator F-254). Compounds were visualized under UV light. $^{1}H$ (including $^{1}H$ DOSY), $^{13}C$ and $^{19}F$ solution-phase NMR spectra were recorded on a Bruker Avance spectrometer operating at 11.7 T (Larmor frequencies of 500, 126 and 471 MHz, respectively). The following abbreviations have been used for multiplicity assignments: "s" for singlet, "d" for doublet, "t" for triplet, "m" for multiplet and "br" for broad. Melting points (Mps) were recorded using open-ended capillaries on an Electrothermal melting point apparatus and are uncorrected. High-resolution mass spectra of ***rac-***, **Λ-** and **Δ-1**, ***rac-***, **Λ-** and **Δ-2** and **Irdmbpy** were recorded at the EPSRC UK National Mass Spectrometry Facility at Swansea University on a quadrupole time-of-flight (ESI-Q-TOF), model ABSciex 5600 Triple TOF in positive electrospray or nanospray ionization mode and spectra were recorded using sodium formate solution as the calibrant. HR-ESI mass spectra of supramolecular cages ***rac-***, **Λ-** and **Δ-C1** and ***rac-***, **Λ-** and **Δ-C2** and mixed-metalloligand cages were recorded at the University of Leeds on a Bruker MaXis Impact instrument in positive ion mode. The HR-ESI mass spectra of **IrCN** was recorded at the University of Leeds on a Bruker MaXis Impact instrument in negative ion mode. The samples were injected by direct infusion from DMSO solutions of concentration of ca. $1\times10^{-4}$ M. The syntheses of 2-(2,4-difluorophenyl)-4-phenyl)pyridine (dFppy),[1] 2-chloro-4-(2,4,6-trimethylphenyl)pyridine,[1] 2-phenyl-4-(2,4,6-trimethylphenyl)pyridine (mesppy),[1] 2-(2,4-difluorophenyl)-4-(2,4,6-trimethylphenyl)pyridine (dFmesppy),[1] and 4,4':2',2":4",4'"-quaterpyridine (qpy)[2] follow previously reported methods.

Synthesis of *rac*-, Λ-, Δ-$[Ir(C^{\wedge}N)_2(qpy)]X^-$; $X^-$ = $PF_6$, $BF_4$ or $SbF_6$.

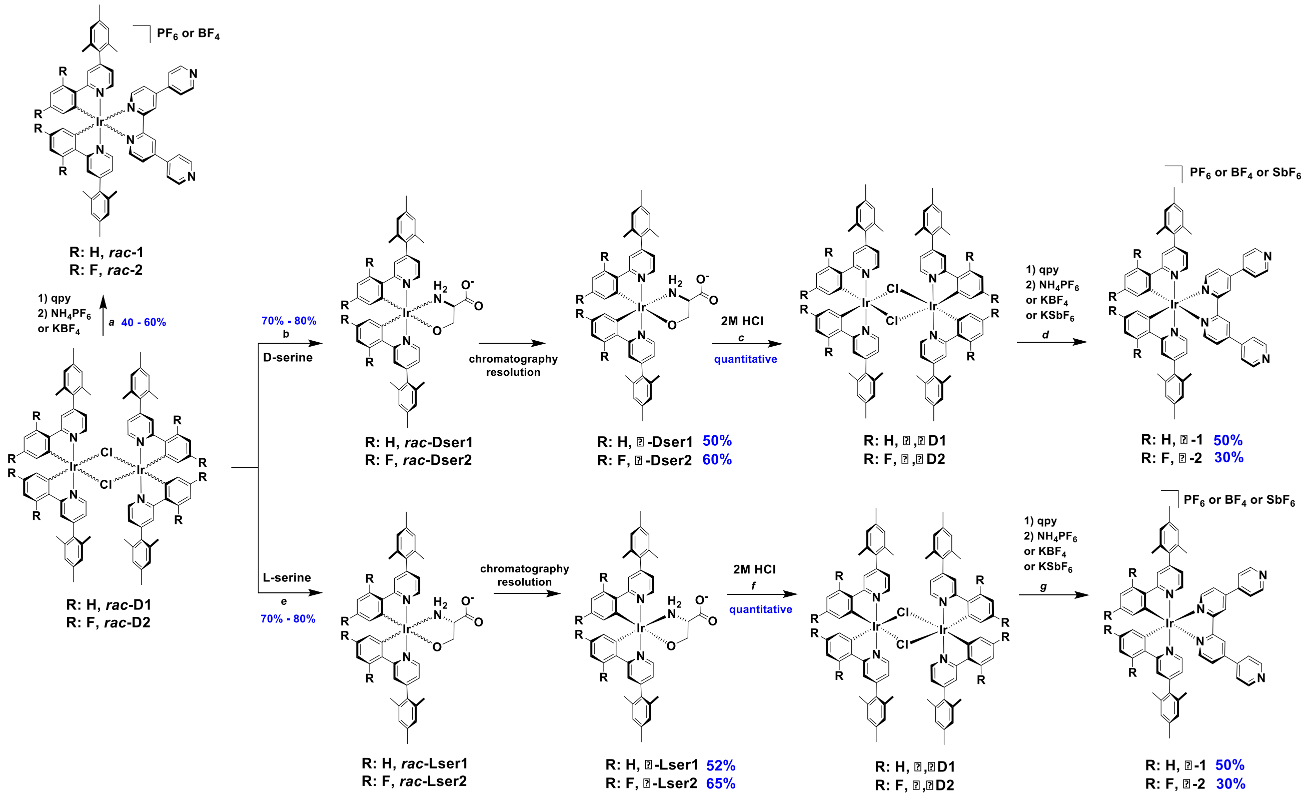


Scheme **S1**. Synthesis of $[Ir(C^{\wedge}N)_2(qpy)]X^-$ ($X^-$ = $PF_6$, $BF_4$ or $SbF_6$) complexes ***rac*-1**, ***rac*-2**, **Δ-1**, **Λ-1, Δ-2, Δ-2**. Reagents and conditions: $^{a,d,g}$ i. 2-$MeOC_2H_4OH$, 110 °C, 19 h; ii. Excess solid $NH_4PF_6$ or $KBF_4$ or $KSbF_6$, $^{b,e}$ $CH_2Cl_2$/MeOH (2:1 v/v), 1.2 equiv. $C_2H_5ONa$, 55 °C, 19 h, $^{c,f}$ MeOH/2M $HCl_{aq}$ (2:1 v/v), 20 °C, 30 min. All reactions were conducted under an $N_2$ atmosphere.

The syntheses of both the *rac*- and Λ- and Δ-enantiopure iridium(III) complexes **1** and **2** are shown in Scheme **S1**. The mesppy and dFmesppy C^N ligands were reacted with $IrCl_3{\cdot}3H_2O$ and the resulting the μ-dichloro-bridged iridium dimers *rac*-$[Ir(mesppy)_2Cl]_2$, ***rac*-D1** and *rac*-$[Ir(dFmesppy)_2Cl]_2$, ***rac*-D2** were formed in good yield under standard conditions.[2] Complexes ***rac*-1** and ***rac*-2** were isolated in moderate yield through cleavage of ***rac*-D1** and ***rac*-D2** with qpy and subsequent anion metathesis with aqueous $NH_4PF_6$ or a methanol solution of $KBF_4$ or a methanol solution of $KSbF_6$. Following a previously reported protocol,[3] treatment of ***rac*-D1** and ***rac*-D2** with

L-serine afforded diastereomers Δ–[Ir(C^N)$_2$(L-serine)] and Λ–[Ir(C^N)$_2$(L-serine)] (respectively, Δ-Lser and Λ-Lser in Scheme **S1**). Chromatographic purification on silica gel permitted only isolation of analytically pure Δ–[Ir(C^N)$_2$(L-serine)], which was the first eluting diastereomer; the second, lower mobility diastereomer, corresponding to Λ–[Ir(C^N)$_2$(L-serine)] was found to be contaminated with traces of the Δ-LSer complex. Fortunately, following chromatographic resolution of the corresponding D-serine complexes, we obtained analytically pure Λ–[Ir(C^N)$_2$(D-serine)] as the first eluting band (Λ-DSer in Scheme **S1**). The $^1$H NMR of the *rac*-[Ir(C^N)$_2$(L-serine)], Δ–[Ir(C^N)$_2$(L-serine)] and Λ–[Ir(C^N)$_2$(D-serine)] complexes are reported in Figures **S1** and **S2**. Addition of a solution of 1M HCl to the Λ- and Δ–[Ir(C^N)$_2$(serine)] complexes yielded the enantiopure Λ,Λ and Δ,Δ-[Ir(C^N)$_2$Cl]$_2$ dimers. Finally, the enantiopure **Λ-1** and **Δ-1** were obtained, respectively, upon reaction of Λ Λ and Δ,Δ-[Ir(mesppy)$_2$Cl]$_2$ dimers with qpy following standard conditions,[2] while the enantiopure **Λ-2** and **Δ-2** were obtained, respectively, by reacting the Λ,Λ- and Δ,Δ-[Ir(dFmesppy)$_2$Cl]$_2$ dimers with qpy. All complexes were purified by column chromatography and isolated as their $PF_6^-$ or $BF_4^-$ or $SbF_6^-$ salts following an anion metathesis reaction using $NH_4PF_6$ or $KBF_4$ or $KSbF_6$. The purity of the complexes was confirmed by $^1$H, $^{19}$F $^{13}$C NMR, HRMS and melting point analyses. The degree of enantiopurity and **Δ-1**, **Λ-1**, **Δ-2** and **Λ-2** was confirmed by circular dichroism (CD) spectroscopy. In addition, X-ray single crystal structures of the enantiopure Λ,Λ- and Δ,Δ-[Ir(dFmesppy)$_2$Cl]$_2$ dimers (**Λ,Λ-D2** and **Δ,Δ-D2**) and of the racemate ***rac*-2** were also obtained (Figure **S59** and **S60**). The crystal structures of the enantiopure Λ,Λ- and Δ,Δ-[Ir(mesppy)$_2$Cl]$_2$ dimers and of the racemate ***rac*-1** were previously reported by our group.[2-3]

*General procedure for the synthesis of rac-[Ir(C^N)$_2$]$_2$Cl$_2$ dimers.* The iridium (III) dimers, [Ir(mesppy)$_2$(μ-Cl)]$_2$ and [Ir(dFmesppy)$_2$(μ-Cl)]$_2$ were prepared according to the procedure described by Nonoyama.[4] Briefly, to $IrCl_3 \cdot 3H_2O$ (1.0 equiv.) and C^N ligand (2.2 equiv.) was added 2-ethoxyethanol to give a concentration of 0.02 M of iridium dimer. The reaction mixture was degassed by multiple vacuum and $N_2$ purging cycles and the mixture was heated to reflux for 19 h. The solution was cooled to room temperature and the yellow precipitate was filtered, washed with water, hexane and ether and finally dried under vacuum to afford the desired material, which was used without further purification.

**Tetrakis[2-(phenyl)-4-(2,4,6-trimethylphenyl)pyridinato-*N*,*C*$^{2'}$]-bis(μ-chloro)diiridium(III)[Ir(mesppy)$_2$(μ-Cl)]$_2$** (*rac*-**D1**). **Yield:** 70%. **$^1$H NMR (500 MHz, $CD_2Cl_2$) δ(ppm):** 9.69 (d, *J* = 6.4 Hz, 2H), 7.77 (s, 4H), 7.55 (d, *J* = 9.0 Hz, 4H), 7.05 (d, *J* = 10.9 Hz, 8H), 6.86 (m, 7H), 6.72 (dt, *J* = 8.1 Hz, 4H), 5.95 (d, *J* = 8.1 Hz, 4H), 2.42 (s, 12H), 2.16 (s, 12H), 2.15 (s, 12H). The characterization matches that reported.[1]

**Tetrakis[2-(4',6'-difluorophenyl)-4-(2,4,6-trimethylphenyl)pyridinato-*N*,*C*$^{2'}$]-bis(μ-chloro)diiridium(III), [Ir(dFmesppy)$_2$(μ-Cl)]$_2$ (*rac*-D2). Yield:** 81%. **$^1$H {$^{19}$F} NMR (400 MHz,$CD_2Cl_2$) δ (ppm):** 9.57 (d, *J* = 6.0 Hz, 4H), 8.13 (s, 4H), 7.02 (d, *J* = 10.4 Hz, 8H), 6.89 (dd, *J* = 6.0, 2.0 Hz, 4H), 5.29 (m, 4H) 2.38 (s, 12H), 2,12 (s, 24H). **$^{19}$F {$^1$H} NMR (471 MHz, $CD_2Cl_2$) δ (ppm):** –108.1 (d, *J* = 11.1 Hz, 4F), –110.2 (d, *J* = 10.8 Hz, 4F)**.** The characterization matches that reported.[1]

*General procedure for the synthesis of* Λ,Λ- and Δ,Δ-*[Ir(C^N)$_2$]$_2$Cl$_2$ dimers.* The enantiopure iridium (III) dimers, Λ,Λ- and Δ,Δ-[Ir(mesppy)$_2$(μ-Cl)]$_2$ (**Λ,Λ-D1** and **Δ,Δ-D1** in Scheme **S1**) Λ,Λ- and Δ,Δ-[Ir(dFmesppy)$_2$(μ-Cl)]$_2$ (**Λ,Λ-D2** and **Δ,Δ-D2** in Scheme **S1**) were prepared according to the procedure previously reported for Λ,Λ- and Δ,Δ-[Ir(mesppy)$_2$(μ-Cl)]$_2$.[3] To a Schlenk tube

containing *rac*-[(Ir(C^N)$_2$Cl)$_2$] (***rac*-D1** or ***rac*-D2** in Scheme **S1**) (0.30 mmol, 1 equiv.), *L* or *D*-serine (0.63 mmol, 2.1 equiv.) and NaOEt (0.63mmol, 2.1 equiv.) were added. DCM and MeOH (1:1 v/v) were added to give a concentration of 0.03 M of iridium dimer. The reaction mixture was degassed by multiple vacuum and $N_2$ purging cycles and the mixture was heated to 50 °C for 19 h under nitrogen atmosphere. The solvent was removed in *vacuo* and the crude product purified using silica-gel flash column chromatography ($CH_2Cl_2$/$CH_3OH$/NEt$_3$ (96:3:1) as eluent). The pure fractions of the higher mobility diastereoisomers, Δ-[Ir(C^N)$_2$(*L*-serine)] (**Δ-Lser** in Scheme **S1**) and Λ-[Ir(C^N)$_2$(*D*-serine)] (**Λ-Dser** in Scheme **S1**) were obtained analytically pure, whereas the slower eluting compounds, Λ-[Ir(C^N)$_2$(*L*-serine)] (**Λ-Lser** in Scheme **S1**) and Δ-[Ir(C^N)$_2$(D-serine)], (**Δ-Dser** in Scheme **S1**) were always contaminated with the other isomer. The pure **Δ-Lser** and **Λ-Dser** complexes isolated from the column were dissolved in $CH_3OH$ and $CH_2Cl_2$ (5:1 v/v) and to this was added 1M HCl solution (2 mL). A precipitate was formed after stirring the solutions for 10 min at room temperature. The solids were filtered, washed with hexane and diethyl ether (1:1 v/v) and air dried to give yellow powders.

**Δ-Iridium(III)bis[2-phenyl-4-(2,4,6-trimethylphenyl)pyridinato]-*L*-serine, Δ-[Ir(mesppy)$_2$(*L*-serine)] (Δ-Lser1). Yield:** 52%. **R$_f$:** 0.35 ($CH_2Cl_2$/$CH_3OH$/NEt$_3$ (96:3:1)). **Mp:** 282 - 286 °C. **$^1$H NMR (500 MHz, $CD_2Cl_2$) δ(ppm)** 9.56 (d, *J* = 6.2 Hz, 2H), 8.73 (d, *J* = 6.1 Hz, 2H), 7.77 (dd, *J* = 0.9, 23.5 Hz, 4H), 7.57 (dt, *J* = 0.4, 5.9 Hz, 4H), 7.22 (dd, *J* = 1.7, 5.7 Hz, 2H), 7.15 (dd, *J* = 1.7, 5.9 Hz, 2H), 7.07 (m, 8H), 6.87 (m, 4H), 6.75 (m, 4H), 6.42 (dd,*J* = 1.0, 8.1 Hz, 2H), 6.31 (dd, *J* = 0.7, 6.7 Hz, 2H), 4.08 (dd, *J* = 5.6 Hz, 2H), 3.89 (dd,*J* = 3.2, 8.5 Hz, 1H), 2.21 (s, 6H), 2.21 (s, 6H), 2.19 (s, 6H).

**Λ-Iridium(III)bis[2-phenyl-4-(2,4,6-trimethylphenyl)pyridinato]-*D*-serine, Λ-[Ir(mesppy)$_2$(*D*-serine)] (Λ-Dser1). Yield:** 52%. **R$_f$:** 0.35 ($CH_2Cl_2$/$CH_3OH$/NEt$_3$ (96:3:1)). **Mp:** 282 - 286 °C. **$^1$H**

**NMR (500 MHz, $CD_2Cl_2$) δ(ppm)** 9.62 (d, *J* = 6.5 Hz, 2H), 8.77 (d, *J* = 5.3 Hz, 2H), 7.72 (dd, *J* = 1.6, 17.8 Hz, 4H), 7.55 (dt, *J* = 1.1, 5.8 Hz, 4H), 7.15 (ddd, *J* = 1.5, 5.5, 9.5 Hz, 2H), 7.04 (m, 8H), 6.84 (m, 4H), 6.73 (m, 4H), 6.47 (dd,*J* = 0.6, 8.0 Hz, 2H), 6.28 (dd, *J* = 0.6, 7.4 Hz, 2H), 3.90 (dd, *J* = 3.7, 11.0 Hz, 2H), 3.82 (dd,*J* = 5.3, 10.8 Hz 1H), 2.21 (s, 6H), 2.21 (s, 6H), 2.19 (s, 6H).

**Δ-Iridium(III)bis[2-(4',6'-difluorophenyl)-4-(2,4,6-trimethylphenyl)pyridinato]-*L*-serine, Δ-[Ir(dFmesppy)₂(*L*-serine)] (Δ-Lser2). Yield:** 55%. **$R_f$:** 0.30 ($CH_2Cl_2/CH_3OH/NEt_3$ (96:3:1)). **Mp:** 292 - 296 °C. **$^1H$ NMR (500 MHz, $CD_2Cl_2$) δ(ppm)** 8.90 (d, *J* = 5.2 Hz, 2H), 8.78 (d, *J* = 5.9 Hz, 2H), 8.18 (dd, *J* = 0.8, 9.6 Hz, 4H), 7.26 (dd, *J* = 1.5, 6.0 Hz, 2H), 7.21 (dd, *J* = 1.7, 5.8 Hz, 2H), 7.08 (bm, 2H), 6.43 (m, 6H), 5.80 (dd, *J* = 2.6, 8.8 Hz, 2H), 5.65 (m, *J* = 2.2, 8.8 Hz, 2H), 5.53 (t, *J* = 1.0 Hz, 2H), 5.18 (t, *J* = 1.1 Hz, 2H), 4.36 (dd, *J* = 2.0, 10.0 Hz, 2H), 3.89 (dd, *J* = 10.1 Hz, 2H), 2.40 (s, 6H), 2.22 (s, 6H), 2.19 (s, 6H). **$^{19}F$ NMR (471 MHz, $CD_2Cl_2$) δ (ppm):** –108.3 (q, *J* = 10.0 Hz, 2F), –108.8 (q, *J* = 10.1 Hz, 2F).

**Λ-Iridium(III)bis[2-(4',6'-difluorophenyl)-4-(2,4,6-trimethylphenyl)pyridinato]-*D*-serine, Λ-[Ir(dFmesppy)₂(*D*-serine)] (Λ-Dser2). Yield:** 55%. **$R_f$:** 0.35 ($CH_2Cl_2/CH_3OH/NEt_3$ (96:3:1)). **Mp:** 292 - 296 °C. **$^1H$ NMR (500 MHz, $CD_2Cl_2$) δ(ppm)** 8.93 (d, *J* = 6.0 Hz, 2H), 8.69 (d, *J* = 6.8 Hz, 2H), 8.15 (dd, *J* = 1.0, 15.6 Hz, 4H), 7.19 (dd, *J* = 1.7, 5.7 Hz, 2H), 7.05 (m, 4H), 6.41 (dt, *J* = 2.4, 8.8 Hz, 2H), 6.29 (dt, *J* = 2.7, 10.2 Hz, 2H), 5.85 (dd, *J* = 2.0, 8.5 Hz, 2H), 5.65 (dd, *J* = 2.6, 9.2 Hz, 2H), 5.53 (t, *J* = 1.2 Hz, 2H), 5.18 (t, *J* = 1.1 Hz, 2H), 4.26 (dd, *J* = 2.2, 10.4 Hz, 2H), 3.84 (m, 2H), 2.40 (s, 6H), 2.39 (s, 6H), 2.20 (s, 6H). **$^{19}F$ NMR (471 MHz, $CD_2Cl_2$) δ (ppm):** –108.4 (q, *J* = 10.0 Hz, 2F), –108.0 (q, *J* = 10.1 Hz, 2F).

**Δ, Δ- a,1 Λ, Λ-Tetrakis[2-(phenyl)-4-(2,4,6-trimethylphenyl)pyridinato-*N*,*C*$^{2'}$]-bis(μ-chloro)diiridium(III)[Ir(mesppy)₂(μ-Cl)]₂ (Δ,Δ-D1 and Λ,Λ-D1). Yield: Δ,Δ-D1**: 5%; **Λ,Λ-D1**: 45%. **$^1H$ NMR (500 MHz, $CD_2Cl_2$) δ(ppm):** 9.69 (d, *J* = 6.4 Hz, 2H), 7.77 (s, 4H), 7.55 (d, *J* = 9.0

Hz, 4H), 7.05 (d, $J$ = 10.9 Hz, 8H), 6.86 (m, 7H), 6.72 (dt, $J$ = 8.1 Hz, 4H), 5.95 (d, $J$ = 8.1 Hz, 4H), 2.42 (s, 12H), 2.16 (s, 12H), 2.15 (s, 12H). The characterization matches that reported.[3]

**Δ, Δ- and Λ, Λ-Tetrakis[2-(4',6'-difluorophenyl)-4-(2,4,6-trimethylphenyl)pyridinato-*N*,*C*$^{2'}$]-bis(μ-chloro)diiridium(III), [Ir(dFmesppy)$_2$(μ-Cl)]$_2$ (Δ,Δ-D2 and Λ,Λ-D2). Yield: Δ,Δ-D2**: 60%; **Λ,Λ-D2**: 50%. **$^1$H {$^{19}$F} NMR (400 MHz,CD$_2$Cl$_2$) δ (ppm):** 9.57 (d, $J$ = 6.0 Hz, 4H), 8.13 (s, 4H), 7.02 (d, $J$ = 10.4 Hz, 8H), 6.89 (dd, $J$ = 6.0, 2.0 Hz, 4H), 5.29 (m, 4H) 2.38 (s, 12H), 2,12 (s, 24H). **$^{19}$F {$^1$H} NMR (471 MHz, CD$_2$Cl$_2$) δ (ppm):** –108.1 (d, $J$ = 11.1 Hz, 4F), –110.2 (d, $J$ = 10.8 Hz, 4F). The characterization matches that reported.[1]

*General procedure for the synthesis of rac-, Λ- and Δ-[Ir(C^N)$_2$(qpy)]PF$_6$, BF$_4$ or SbF$_6$ complexes.* To a Schlenk tube containing one of *rac*-[Ir(mesppy)$_2$Cl]$_2$ (***rac*-D1**), *rac*-[Ir(dFmesppy)$_2$Cl]$_2$, (***rac*-D2**), Λ, Λ-[Ir(mesppy)$_2$Cl]$_2$, (**Λ,Λ-D1**), Δ, Δ-[Ir(mesppy)$_2$Cl]$_2$, (**Δ,Δ-D1**), Λ, Λ-[Ir(dFppymes)$_2$Cl]$_2$, (**Λ,Λ-D2**) or Δ, Δ-[Ir(dFppymes)$_2$Cl]$_2$, (**Δ,Δ-D2**) (1.0 equiv.), and qpy ligand (2.5 equiv.), was added 2-methoxyethanol to give a concentration of 0.03 M of iridium dimer. The reaction mixture was degassed by multiple vacuum and $N_2$ purging cycles and the mixture was heated to 55 °C for 19 h under nitrogen atmosphere. For preparing the $PF_6^-$ salts: the solution was cooled to room temperature and solid $NH_4PF_6$ (10.0 equiv.) was added and the reaction mixture was left to stir for a further 1 h. For preparing the $BF_4^-$ salts: a 2 M solution of $KBF_4$ in MeOH (10 mL) was added at room temperature to the reaction mixture and the resulting suspension was stirred at room temperature for 4 h. For preparing $SbF_6^-$ salts: a 2 M solution of $KSbF_6$ in MeOH (10 mL) was added at room temperature to the reaction mixture and the resulting suspension stirred at room temperature for 4 h. For all three anions, the resulting suspensions were evaporated to dryness, with the residue then copiously washed with $Et_2O$ and distilled water. This crude product was purified by

flash column chromatography (silica, DCM/MeOH/$NEt_3$ gradient 100:0:0 to 60:40:10). Fractions containing the desired complex were combined and solid $NH_4PF_6$ (10.0 equiv.) or $KBF_4$ (10.0 equiv.) or $SbF_6$ (10.0 equiv.) was added. The suspension was stirred at room temperature for 0.5 h. This mixture was then evaporated to dryness, washed vigorously with distilled water and diethyl ether and dried to afford the pure material.

***rac*-, Δ-, Λ–Iridium(III)bis[2-phenyl-4-(2,4,6-trimethylphenyl)pyridinato]-4,4':2',2'':4'',4'''-quaterpyridine hexafluorophosphate, Δ-, Λ-[Ir(mesppy)$_2$(qpy)](PF$_6$)** (***rac*-, Δ-, Λ-1**): **Yield**: ***rac*-1**: 55%; **Δ-1**: 45%; **Λ-1** 50%. **R*f***: 0.20 (DCM : MeOH : $NEt_3$ = 100 : 3 : 2). **Mp**: ***rac*-1**; 350 – 353 °C (decomposed); **Δ-1**; 351 - 354 °C (decomposed); **Λ-1**; 348 – 352 °C (decomposed). **$^1$H NMR (500 MHz, CDCl$_3$) δ(ppm)**: 9.51 (s, 2H), 8.88 (d, *J* = 5.7 Hz, 4H), 8.25 (dd, *J* = 6.1, 2.1 Hz, 2H), 8.17 (s, 2H), 8.12 (d, *J* = 5.6 Hz, 2H), 8.08 (d, *J* = 6.7 Hz, 3H), 8.03 (d, *J* = 7.8 Hz, 2H), 7.78 (d, *J* = 5.6 Hz, 2H), 7.05 - 6.98 (m, 10H), 6.30 (d, *J* = 6.3 Hz, 2H), 2.29 (s, 6H), 2.10 (s, 6H), 1.90 (s, 6H). **$^{13}$C NMR (126 MHz, CDCl$_3$) δ(ppm)**: 167.8, 165.5, 152.4, 151.5, 150.9, 149.8, 149.0, 148.6, 143.9, 143.0, 138.3, 134.9, 133.2, 131.5, 130.8, 128.5, 126.2, 125.1, 124.9, 122.9, 122.3, 121.3, 29.7, 20.8, 20.3. **HR NSI$^+$ MS**: [M-PF$_6$]$^+$ Calculated: ($C_{60}H_{50}IrN_6$): 1047.3726 Found: **Δ-1**; 1047.3719; **Λ-1**; 1047.3718. The characterization of ***rac*-1** matches that previously reported.[2]

***rac*-, Δ-, Λ–Iridium(III)bis[2-(2,4-difluoro)-phenyl-4-(2,4,6-trimethylphenyl)pyridinato]-4,4':2',2'':4'',4'''-quaterpyridine hexafluorophosphate, Δ-, Λ-[Ir(dFmesppy)$_2$(qpy)](PF$_6$) (*rac*-, Δ-, Λ-2). Yield: *rac*-2**; 40%; **Δ-2;** 38%; **Λ-2**; 30%. **R*f*:** 0.20 (DCM : MeOH : $NEt_3$ = 100 : 3 : 2). **Mp: *rac*-2**; 342 - 346 °C (decomposed); **Δ-2**; 340 - 344 °C (decomposed); **Λ-2**; 348 - 352 °C (decomposed). **$^1$H NMR (500 MHz, CD$_2$Cl$_2$) δ(ppm):** 8.83 (m, 5H), 8.31 (d, *J* = 5.4 Hz, 1H), 8.22 (bs, 2H), 7.85 (d, *J* = 5.9 Hz, 1H), 7.80 (d, *J* = 5.3 Hz, 2H), 7.69 (d, *J* = 5.8 Hz, 2H), 7.04 (m, 6H), 6.68 (m, 3H), 5.81 (m, 3H), 2.35 (s, 6H), 2.16 (s, 6H), 1.99 (s, 6H). **$^{19}$F NMR (417 MHz, CD$_2$Cl$_2$) δ**

**(ppm):** –72.5 (d,$J$ = 707.5 Hz,6F), –106.0(d, $J$ = 10.3 Hz, 2F), –108.3(d, $J$ = 10.3 Hz, 2F). **HR NSI$^+$ MS:** [M-$PF_6$]$^+$ Calculated**:** ($C_{60}H_{46}F_4IrN_6$): 1119.3349 **Found: Δ-2**; 1119.3326; **Λ-2**; 1119.3324. The characterization of ***rac*-2** matches that reported.[2]

*rac-,* **Δ-, Λ–Iridium(III)bis[2-phenyl-4-(2,4,6-trimethylphenyl)pyridinato]-4,4':2',2'':4'',4'''-quaterpyridine tetrafluoroborate, Δ-, Λ-[Ir(mesppy)$_2$(qpy)](BF$_4$) (*rac*-, Λ-, Δ-1)**: **Yield:** ***rac*-1**; 55%; **Δ-1**; 52%; **Λ-1**; 50%. $R_f$: 0.20 (DCM + 3% MeOH + 2% $NEt_3$). **Mp:** ***rac*-1**; 348 - 352 °C (decomposed); **Δ-1**; 351 - 354 °C (decomposed); **Λ-1**; 355 - 358 °C (decomposed). **$^1$H NMR (500 MHz, $CDCl_3$) δ(ppm):** 9.20 (s, 2H), 8.86 (d, $J$ = 5.7 Hz, 4H), 8.30 (d, $J$ = 5.3 Hz, 2H), 7.91 (m, 4H), 7.84 (s, 2H), 7.81 (dd, $J$ = 1.5, 6.1 Hz, 2H), 7.76 (d, $J$ = 7.8 Hz, 2H), 7.69 (d, $J$ = 6.8 Hz, 2H), 7.14 (dt, $J$ = 1.0, 6.9 Hz, 2H), 7.05 (m, 7H), 6.93 (dd, J = 0.5, 6.4 Hz, 2H), 6.46 (d, $J$ = 6.4 Hz, 2H), 2.36 (s, 6H), 2.17 (s, 6H), 1.99 (s, 6H). **HR NSI$^+$ MS**: [M-$BF_4$]$^+$ Calculated: ($C_{60}H_{50}IrN_6$): 1047.3726 Found: ***rac*-1**; 1047.3719; **Δ**-1 ; 1047.3719; **Λ-1**: 1047.3715.

*rac-,* **Δ-, Λ–Iridium(III)bis[2-(2,4-difluoro)-phenyl-4-(2,4,6-trimethylphenyl)pyridinato]-4,4':2',2'':4'',4'''-quaterpyridine tetrafluoroborate, Δ-, Λ-[Ir(dFmesppy)$_2$(qpy)](BF$_4$) (*rac*-, Δ-, Λ-2). Yield:** ***rac*-2**; 40%; **Δ-2**; 38%; **Λ-2**; 35%.. **$R_f$:** 0.20 (DCM : MeOH :$NEt_3$ = 100 : 3 : 2). **Mp:** ***rac*-2**; 344 - 348 °C (decomposed); **Δ-2**; 340 - 344 °C (decomposed); **Λ-2**; 341 - 345 °C (decomposed). **$^1$H NMR (500 MHz, $CD_2Cl_2$) δ(ppm):** 9.27 (s, 2H), 8.86 (s, 4H), 8.25 (m, 4H), 7.90 (m, 6H), 7.70 (d, $J$ = 5.6 Hz, 2H), 7.01 (m, 2H), 6.68 (t, $J$ = 11.1 Hz, 2H), 6.68 (m, 3H), 5.83 (d, $J$ = 6.5 Hz, 2H), 2.35 (s, 6H), 2.16 (s, 6H), 1.99 (s, 6H). **$^{19}$F NMR (417 MHz, $CD_2Cl_2$) δ (ppm):**–106.2 (d, $J$ = 10.3 Hz, 2F), –108.4 (d, $J$ = 10.3 Hz, 2F), –150.9 (d, $J$ = 10 Hz, 4F). **HR NSI$^+$ MS:** [M-$BF_4$]$^+$ Calculated: ($C_{60}H_{46}F_4IrN_6$): 1119.3349 **Found:** ***rac*-2;** 1119.3326; **Δ-2**; 1119.3326, **Λ-2**; 1119.3325.

**Δ−Iridium(III)bis[2-phenyl-4-(2,4,6-trimethylphenyl)pyridinato]-4,4':2',2":4",4'"-quaterpyridine hexafluoroantimonate, Δ−[Ir(mesppy)$_2$(qpy)](SbF$_6$) (Δ−1)**: **Yield:** 55%. R$_f$: 0.20 (DCM : MeOH : NEt$_3$ = 100 : 3 : 2). **Mp:** 358 - 360 °C (decomposed). **$^1$H NMR (500 MHz, CDCl$_3$) δ(ppm):** δ 9.51 (d, $J$ = 2.0 Hz, 2H), 8.99 – 8.81 (m, 4H), 8.25 (dd, $J$ = 5.8, 1.8 Hz, 2H), 8.17 (d, $J$ = 1.9 Hz, 2H), 8.11 (d, $J$ = 5.8 Hz, 2H), 8.08 (d, $J$ = 6.3 Hz, 4H), 8.03 (dd, $J$ = 7.7, 1.5 Hz, 2H), 7.78 (d, $J$ = 6.0 Hz, 2H), 7.09 – 6.90 (m, 10H), 6.33 – 6.25 (m, 2H), 2.28 (s, 6H), 2.09 (s, 6H), 1.90 (s, 6H). (s, 6H). **HR NSI$^+$ MS**: [M-SbF$_6$]$^+$ Calculated: ($C_{60}H_{50}IrN_6$): 1047.3726 Found: 1047.3719.

*The general procedures for the synthesis of rac-TBA[Ir(dFppy)$_2$(CN)$_2$] (IrCN)*[5] *and [Ir(dFppy)$_2$(dmbpy)](PF$_6$) (Irdmbpy)*[6] *are according to those previously reported.*

***rac*-Tetrabutyl ammonium bis(cyanide)-bis[2-(2,4-difluoro)-phenylpyridinato]-iridium(III), *rac*-TBA[Ir(dFppy)$_2$(CN)$_2$] (IrCN).** The synthesis of this complex follows a previously reported method.[5] **Yield:** 70%. **$^1$H NMR (500 MHz, CD$_2$Cl$_2$) δ(ppm):** δ 9.54 (d, $J$ = 5.8 Hz, 2H), 8.36 – 7.86 (m, 5H), 7.45 (t, $J$ = 6.8 Hz, 2H), 6.61 (t, $J$ = 11.3 Hz, 3H), 5.53 (d, $J$ = 8.1 Hz, 2H). **$^{19}$F NMR (417 MHz, CD$_2$Cl$_2$) δ (ppm):** –109.9 (d, $J$ = 11.3 Hz, 2F), –111.0 (d, $J$ = 11.3 Hz, 2F). **HR ESI$^-$ MS: [TBA-M]$^-$ Calculated:** ($C_{24}H_{12}F_4IrN_4$): 625.0627 **Found:** 625.0925. The characterization matches that reported.[5]

***rac*-Iridium(III)bis[2-(2,4-difluoro)-phenylpyridinato]-4,4'-dimethyl-2,2'-bypiridine hexafluorophosphate, [Ir(dFppy)$_2$(dmbpy)](PF$_6$) (Irdmbpy).** The synthesis of this complex follows a previously reported method.[6] **Yield:** 80%. **R$_f$:** 0.20 (DCM + 3% MeOH). **Mp:** 290 - 300 °C. **$^1$H NMR (500 MHz, CD$_2$Cl$_2$) δ(ppm):** δ 8.47 – 8.26 (m, 2H), 7.92 – 7.78 (m, 2H), 7.60 – 7.48 (m, 1H), 7.33 (d, $J$ = 5.2 Hz, 1H), 7.09 (ddd, $J$ = 7.4, 5.8, 1.5 Hz, 1H), 6.64 (ddd, $J$ = 12.1, 9.2, 2.4

Hz, 1H), 5.78 (dd, $J$ = 8.4, 2.4 Hz, 1H). **$^{19}$F NMR (417 MHz, $CD_2Cl_2$) δ (ppm):** –109.0 (d, $J$ = 13.4 Hz, 2F), –106.6 (d, $J$ = 13.2 Hz, 2F), –74.0 (s, 3F), –72.2 (s, 3F). **HR NSI$^+$ MS: [M-$PF_6$]$^+$ Calculated:** ($C_{34}H_{24}F_4IrN_4$): 757.1549 **Found:** 757.1562. The characterization matches that reported.[6]

*General procedure for the synthesis of cages* ***rac-, Λ-*** *and* ***Δ-C1*** *and* ***rac-, Λ-*** *and* ***Δ-C2***. In a dry 10 mL Schlenk vial, one of the complexes ***rac-***, **Λ-** or **Δ-1** or ***rac-***, **Λ-** or **Δ-2** (2 equiv.) and $[Pd(NCMe)_4](BF_4)_2$ (1 equiv.) were dissolved in DMSO-$d_6$ (1 mL) to give a concentration of the iridium metallo-ligands of approximately 0.05 M. The solution was degassed for five minutes by bubbling nitrogen and heated at 85 °C for 12 h under a nitrogen atmosphere. The solution was cooled to room temperature and the black solid was filtered through celite. A 2 M aqueous solution of $NH_4PF_6$ (5 mL) or a 2M MeOH/$H_2O$ solution (1:1 v/v) of $KBF_4$ (5 mL) or a 2M MeOH/$H_2O$ solution (1:1 v/v) of $SbF_6$ (5 mL) was added to the resulting DMSO solution and the mixture was stirred at room temperature for 4h. The solution was cooled in an ice bath for 30 minutes and the obtained precipitate was filtered, washed with water and diethyl ether to yield a dark red solid (***rac-***, **Λ-** or **Δ-C1**) or a dark yellow solid (***rac-***, **Λ-** or **Δ-C2**).

***$Δ_8$-[$1_8Pd_4$]($PF_6$)$_{16}$, ($Δ_8$-C1-$PF_6$):*** **Yield: 90%**. **$^1$H NMR (500 MHz, $CDCl_3$) δ(ppm):** δ 9.67 (d, $J$ = 12.2 Hz, 16H), 9.05 (d, $J$ = 36.6 Hz, 11H), 8.84 (s, 15H), 8.44 – 8.22 (m, 19H), 8.06 (dd, $J$ = 45.9, 31.0 Hz, 38H), 7.72 (s, 6H), 7.44 (s, 7H), 7.13 – 6.68 (m, 46H), 6.57 (s, 4H), 6.22 (d, $J$ = 10.4 Hz, 12H), 2.32 (s, 48H), 2.12 (s, 48H), 1.95 (s, 48H). **$^{19}$F NMR {$PF_6^-$} (417 MHz, $CD_2Cl_2$) δ (ppm):** –69.348 (bs), –70.859 (bs). **HR ESI MS: [M-$PF_6$]$^+$ Calculated:** $[(C_{60}H_{50}IrN_6)_8Pd_4(PF_6)_{12}]^{4+}$: 2635.5650 Found: 2635.7938; $[(C_{60}H_{50}IrN_6)_8Pd_4(PF_6)_{11}]^{5+}$: 2079.6497 Found: 2079.6421; $[(C_{60}H_{50}IrN_6)_8Pd_4(PF_6)_{10}]^{6+}$: 1708.7120 Found: 1708.7056; $[(C_{60}H_{50}IrN_6)_8Pd_4(PF_6)_9]^{7+}$: 1404.1881 Found: 1404.0402; $[(C_{60}H_{50}IrN_6)_8Pd_4(PF_6)_8]^{8+}$: 1245.4881 Found: 1245.4146.

***Λ₈-[1₈Pd₄](PF₆)₁₆, (Λ₈-C1-PF₆):*** **Yield: 90%**. **$^{1}$H NMR (500 MHz, CDCl$_3$) δ(ppm):** $^{1}$H NMR (500 MHz, DMSO-$d_6$) δ 9.67 (d, *J* = 11.8 Hz, 16H), 9.58 – 9.40 (m, 11H), 9.33 (s, 7H), 9.21 – 8.77 (m, 28H), 8.61 – 7.85 (m, 104H), 7.74 (d, *J* = 16.8 Hz, 14H), 7.44 (s, 8H), 7.14 – 6.68 (m, 82H), 6.57 (s, 7H), 6.35 – 6.12 (m, 20H), 2.31 (s, 48H), 2.12 (s, 48H), 1.95 (s, 48H). **$^{19}$F NMR {$PF_6^-$} (417 MHz, $CD_2Cl_2$) δ (ppm):** –69.348 (bs), –70.859 (bs). **HR ESI MS: [M-PF$_6$]$^+$ Calculated:** $[(C_{60}H_{50}IrN_6)_8Pd_4(PF_6)_{12}]^{4+}$: 2635.5650 Found: 2635.7938; $[(C_{60}H_{50}IrN_6)_8Pd_4(PF_6)_{11}]^{5+}$: 2079.6497 Found: 2079.6421; $[(C_{60}H_{50}IrN_6)_8Pd_4(PF_6)_{10}]^{6+}$: 1709.0431 Found: 1708.7056; $[(C_{60}H_{50}IrN_6)_8Pd_4(PF_6)_9]^{7+}$: 1404.1881 Found: 1404.0402; $[(C_{60}H_{50}IrN_6)_8Pd_4(PF_6)_8]^{8+}$: 1245.2926 Found: 1245.4146.

***Δ₈-[1₈Pd₄](BF₄)₁₆, (Δ₈-C1-BF₄):*** **Yield: 95%**. **$^{1}$H NMR (500 MHz, CDCl$_3$) δ(ppm):** δ 9.68 (d, *J* = 14.2 Hz, 16H), 9.10 (d, *J* = 42.7 Hz, 11H), 8.34 (s, 20H), 8.24 – 7.88 (m, 38H), 7.76 (s, 6H), 7.43 (s, 5H), 7.10 – 6.73 (m, 45H), 6.54 (s, 6H), 6.22 (d, *J* = 12.6 Hz, 11H), 2.35 (s, 48H), 2.13 (s, 48H), 1.91 (s, 48H). **$^{19}$F NMR {$BF_4^-$} (417 MHz, $CD_2Cl_2$) δ (ppm):** –148.098 (d, *J* = 10.3 Hz, 4F). **HR ESI MS: [M-BF$_4$]$^+$ Calculated:** $[(C_{60}H_{50}IrN_6)_8Pd_4(BF_4)_{11}]^{5+}$: 1951.3302 Found: 1951.7289; $[(C_{60}H_{50}IrN_6)_8Pd_4(BF_4)_{10}]^{6+}$: 1612.9401 Found: 1612.1035; $[(C_{60}H_{50}IrN_6)_8Pd_4(BF_4)_9]^{7+}$: 1369.2538 Found: 1369.2337; $[(C_{60}H_{50}IrN_6)_8Pd_4(BF_4)_8]^{8+}$: 1187.3291 Found: 1187.3267.

***Λ₈-[1₈Pd₄](BF₄)₁₆, (Λ₈-C1-BF₄):*** **Yield: 95%**. **$^{1}$H NMR (500 MHz, CDCl$_3$) δ(ppm):** $^{1}$H NMR (500 MHz, DMSO-$d_6$) δ 9.71 (s, 16H), 9.12 (d, *J* = 40.3 Hz, 8H), 8.94 (s, 8H), 8.35 (s, 18H), 8.22 – 7.93 (m, 49H), 7.77 (s, 9H), 7.13 – 6.77 (m, 62H), 6.55 (s, 5H), 6.37 – 6.10 (m, 15H). 2.35 (s, 48H), 2.13 (s, 48H), 1.91 (s, 48H). **$^{19}$F NMR {$BF_4^-$} (417 MHz, $CD_2Cl_2$) δ (ppm):** –148.098 (d, *J* = 10.3 Hz, 4F). **HR ESI MS: [M-BF$_4$]$^+$ Calculated:** $[(C_{60}H_{50}IrN_6)_8Pd_4(BF_4)_{11}]^{5+}$: 1951.9102 Found: 1951.7289; $[(C_{60}H_{50}IrN_6)_8Pd_4(BF_4)_{10}]^{6+}$: 1610.9905 Found: 1612.1035; $[(C_{60}H_{50}IrN_6)_8Pd_4(BF_4)_9]^{7+}$: 1369.2518 Found: 1369.2337; $[(C_{60}H_{50}IrN_6)_8Pd_4(BF_4)_8]^{8+}$: 1187.3172 Found: 1187.3267.

***rac-[$1_8Pd_4$]($BF_4$)$_{16}$, (rac-C1-$BF_4$):*** **Yield: 90%**. **$^1$H NMR (500 MHz, $CDCl_3$) δ(ppm):** δ 9.67 (d, *J* = 14.0 Hz, 16H), 9.08 (d, *J* = 43.7 Hz, 11H), 8.37 (s, 20H), 8.25 – 7.84 (m, 38H), 7.76 (s, 6H), 7.41 (s, 5H), 7.09 – 6.71 (m, 44H), 6.53 (s, 6H), 6.20 (d, *J* = 11.4 Hz, 11H), 2.34 (s, 48H), 2.15 (s, 48H), 1.95 (s, 48H). **$^{19}$F NMR {$BF_4^-$} (417 MHz, $CD_2Cl_2$) δ (ppm):** –148.093 (d, *J* = 10.0 Hz, 4F). **HR ESI MS: [M-$BF_4$]$^+$ Calculated:** $[(C_{60}H_{50}IrN_6)_8Pd_4(BF_4)_{11}]^{5+}$: 1951.3302 Found: 1951.7285; $[(C_{60}H_{50}IrN_6)_8Pd_4(BF_4)_{10}]^{6+}$: 1612.9401 Found: 1612.1056; $[(C_{60}H_{50}IrN_6)_8Pd_4(BF_4)_9]^{7+}$: 1369.2538 Found: 1369.2345; $[(C_{60}H_{50}IrN_6)_8Pd_4(BF_4)_8]^{8+}$: 1187.3291 Found: 1187.3294.

***$\Delta_8$-[$1_8Pd_4$]($SbF_6$)$_{16}$, ($\Delta_8$-C1-$SbF_6$):*** **Yield: 95%**. **$^1$H NMR (500 MHz, $CDCl_3$) δ(ppm):** δ 9.68 (s, 1H), 9.07 (d, *J* = 30.1 Hz, 1H), 8.30 (s, 1H), 8.20 – 7.87 (m, 2H), 7.71 (d, *J* = 17.5 Hz, 0H), 7.46 (s, 1H), 7.13 – 6.50 (m, 3H), 6.20 (d, *J* = 34.6 Hz, 1H), 2.34 (s, 48H), 2.11 (s, 48H), 1.95 (s, 48H). **$^{19}$F NMR {$SbF_6^-$} (417 MHz, $CD_2Cl_2$) δ (ppm):** –148.104 (d, *J* = 10.3 Hz, 6F). **HR ESI MS: [M-PF6]$^+$ Calculated:** $[(C_{60}H_{50}IrN_6)_8Pd_4(BF_4)_{11}]^{5+}$: 1951.9102 Found: 1951.7289; $[(C_{60}H_{50}IrN_6)_8Pd_4(BF_4)_{10}]^{6+}$: 1610.9905 Found: 1612.1035; $[(C_{60}H_{50}IrN_6)_8Pd_4(BF_4)_9]^{7+}$: 1369.2518 Found: 1369.2337; $[(C_{60}H_{50}IrN_6)_8Pd_4(BF_4)_8]^{8+}$: 1187.3172 Found: 1187.3267.

***$\Delta_8$-[$2_8Pd_4$]($BF_4$)$_{16}$, ($\Delta_8$-C2-$BF_4$):*** **Yield: 88%**. **$^1$H NMR (500 MHz, $CDCl_3$) δ(ppm):** $^1$H NMR (400 MHz, DMSO-$d_6$) δ 9.72 (s, 16H), 8.27 (d, *J* = 75.6 Hz, 34H), 7.96 (s, 15H), 7.22 – 6.72 (m, 28H), 5.63 (s, 11H), 2.37 (s, 48H), 2.10 (s, 48H), 1.96 (s, 48H). **$^{19}$F NMR (417 MHz, $CD_2Cl_2$) δ (ppm):** –106.066 (bs, 32F), -108.331 (bs, 32F), -148.108 (bd, *J* = 15.3 Hz), -146.175 (bd, *J* = 18.1 Hz). **HR ESI MS: [M-$BF_4$]$^+$ Calculated:** $[(C_{60}H_{46}F_4IrN_6)_8Pd_4(BF_4)_{11}]^{5+}$: 2067.0798 Found: 2067.0646; $[(C_{60}H_{46}F_4IrN_6)_8Pd_4(BF_4)_{10}]^{6+}$: 1707.9986 Found: 1708.0532; $[(C_{60}H_{46}F_4IrN_6)_8Pd_4(BF_4)_9]^{7+}$: 1451.4807 Found: 1461.6165; $[(C_{60}H_{46}F_4IrN_6)_8Pd_4(BF_4)_8]^{8+}$: 1259.2943 Found: 1259.2890.

***$Λ_8$-[$2_8$Pd$_4$](BF$_4$)$_{16}$, ($Λ_8$-C2-BF$_4$):*** **Yield: 88%**. **$^1$H NMR (500 MHz, CDCl$_3$) δ(ppm):** $^1$H NMR (400 MHz, DMSO-$d_6$) δ 9.73 (s, 16H), 9.54 (s, 10H), 9.17 (d, $J$ = 29.2 Hz, 16H), 8.98 (s, 13H), 8.37 (s, 21H), 8.22 (d, $J$ = 33.3 Hz, 51H), 7.94 (d, $J$ = 60.4 Hz, 35H), 7.17 (s, 11H), 7.13 – 6.77 (m, 62H), 6.67 (s, 9H), 2.35 (s, 48H), 2.09 (s, 48H), 1.94 (s, 48H). **$^{19}$F NMR (417 MHz, CD$_2$Cl$_2$) δ (ppm):** –106.274 (bs, 32F), -108.477 (bs, 32F), -148.103 (bd, $J$ = 15.3 Hz), -146.209 (bd, $J$ = 18.1 Hz). **HR ESI MS: [M-BF$_4$]$^+$ Calculated:** [(C$_{60}$H$_{46}$F$_4$IrN$_6$)$_8$Pd$_4$(BF$_4$)$_{11}$]$^{5+}$: 2067.1791 Found: 2067.0646; [(C$_{60}$H$_{46}$F$_4$IrN$_6$)$_8$Pd$_4$(BF$_4$)$_{10}$]$^{6+}$: 1707.8640 Found: 1708.0532; [(C$_{60}$H$_{46}$F$_4$IrN$_6$)$_8$Pd$_4$(BF$_4$)$_9$]$^{7+}$: 1451.5934 Found: 1461.6165; [(C$_{60}$H$_{46}$F$_4$IrN$_6$)$_8$Pd$_4$(BF$_4$)$_8$]$^{8+}$: 1259.2675 Found: 1259.2890.

***rac-[$2_8$Pd$_4$](BF$_4$)$_{16}$, (rac-C2-BF$_4$):*** **Yield: 86%**. **$^1$H NMR (500 MHz, CDCl$_3$) δ(ppm):** $^1$H NMR (400 MHz, DMSO-$d_6$) δ 9.71 (s, 16H), 8.29 (d, $J$ = 73.6 Hz, 34H), 7.93 (s, 14H), 7.21 – 6.75 (m, 28H), 5.60 (s, 10H), 2.37 (s, 48H), 2.13 (s, 48H), 1.95 (s, 48H). **$^{19}$F NMR (417 MHz, CD$_2$Cl$_2$) δ (ppm):** –106.064 (bs, 32F), -108.329 (bs, 32F), -148.112 (bd, $J$ = 15.3 Hz), -146.174 (bd, $J$ = 17.9 Hz). **HR ESI MS: [M-BF$_4$]$^+$ Calculated:** [(C$_{60}$H$_{46}$F$_4$IrN$_6$)$_8$Pd$_4$(BF$_4$)$_{11}$]$^{5+}$: 2067.0798 Found: 2067.0695; [(C$_{60}$H$_{46}$F$_4$IrN$_6$)$_8$Pd$_4$(BF$_4$)$_{10}$]$^{6+}$: 1707.9986 Found: 1708.0556; [(C$_{60}$H$_{46}$F$_4$IrN$_6$)$_8$Pd$_4$(BF$_4$)$_9$]$^{7+}$: 1451.4815 Found: 1451.6469; [(C$_{60}$H$_{46}$F$_4$IrN$_6$)$_8$Pd$_4$(BF$_4$)$_8$]$^{8+}$: 1259.2943 Found: 1259.2897.

# Characterization of [Ir(C^N)$_2$(Serine)]PF$_6$

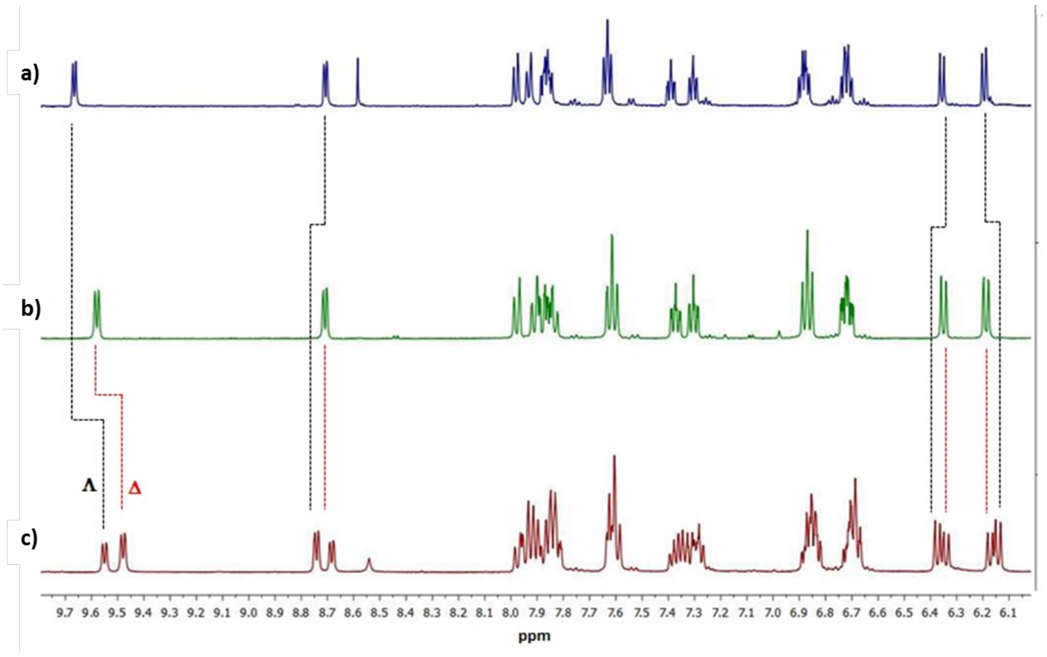


Figure **S1.** $^{1}$H NMR spectra (aromatic region) of **a)** Λ-[Ir(ppymes)$_2$(*D*-serine)] **(Λ-Dser1), b)** Δ-[Ir(ppymes)$_2$(*L*-serine)] **(Δ-Lser1)** and **c)** *rac*-[Ir(ppymes)$_2$(*L*-serine] **(rac-Lser1)** in $CD_2Cl_2$.

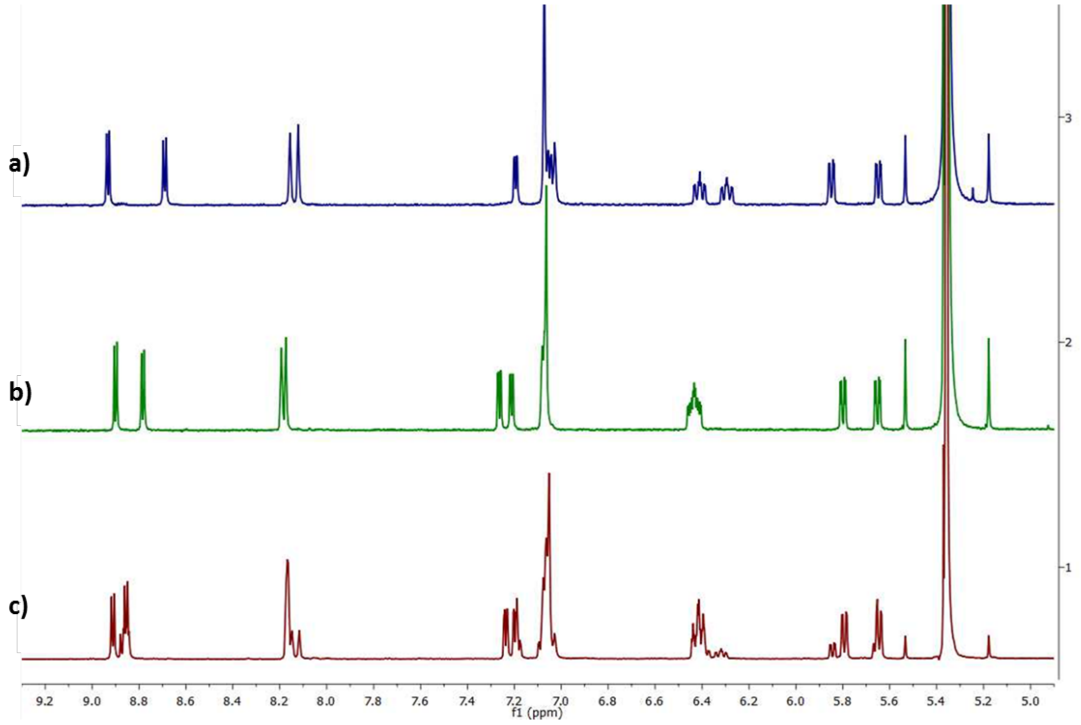


Figure **S2.** $^{1}$H NMR spectra (aromatic region) of **a)** Λ-[Ir(dFmesppy)$_2$(*D*-serine)] **(Λ-Dser2), b)** Δ-[Ir(dFmesppy)$_2$(*L*-serine)] **(Δ-Lser2)** and **c)** *rac*-[Ir(dFmesppy)$_2$(*L*-serine)] **(rac-Lser2)** in $CD_2Cl_2$.

# Characterization of [Ir(C^N)$_2$(N^N)]PF$_6$ Complexes.

### *rac*-, Δ− and Λ−Iridium(III)bis[2-phenyl-4-(2,4,6-trimethylphenyl)pyridinato]-4,4':2',2'':4'',4'''-quaterpyridine hexafluorophosphate, *rac*-, Δ− and Λ−[Ir(mesppy)$_2$(qpy)](PF$_6$).

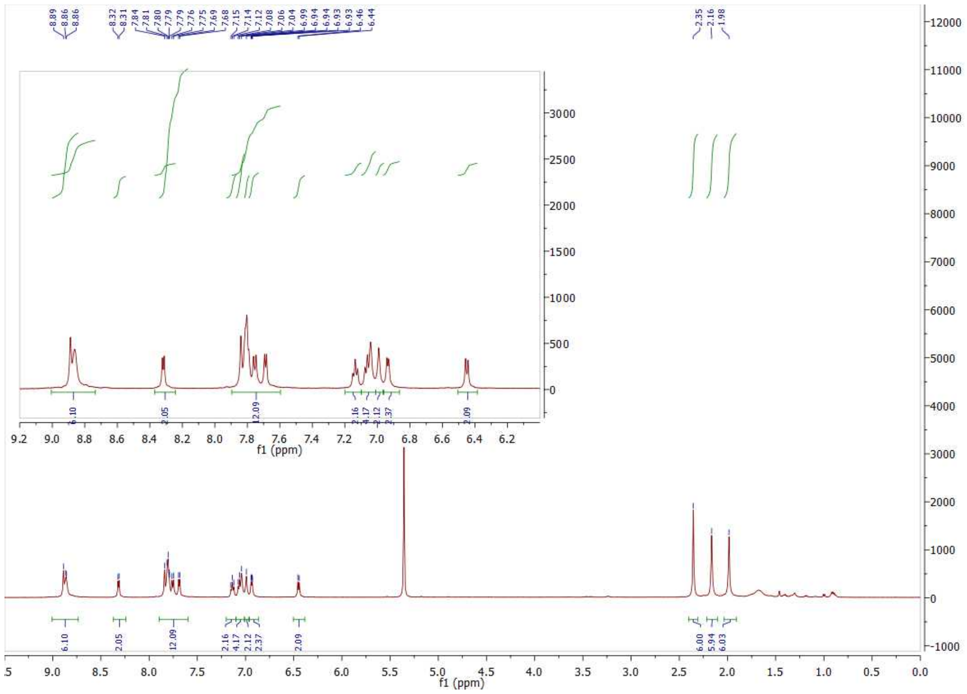


Figure **S3.** $^1$H NMR spectrum of ***rac*-**, **Λ−** and **Δ-[Ir(mesppy)$_2$(qpy)]PF$_6$** (***rac*-, Λ−** and **Δ-1-PF$_6$**) in CD$_2$Cl$_2$.

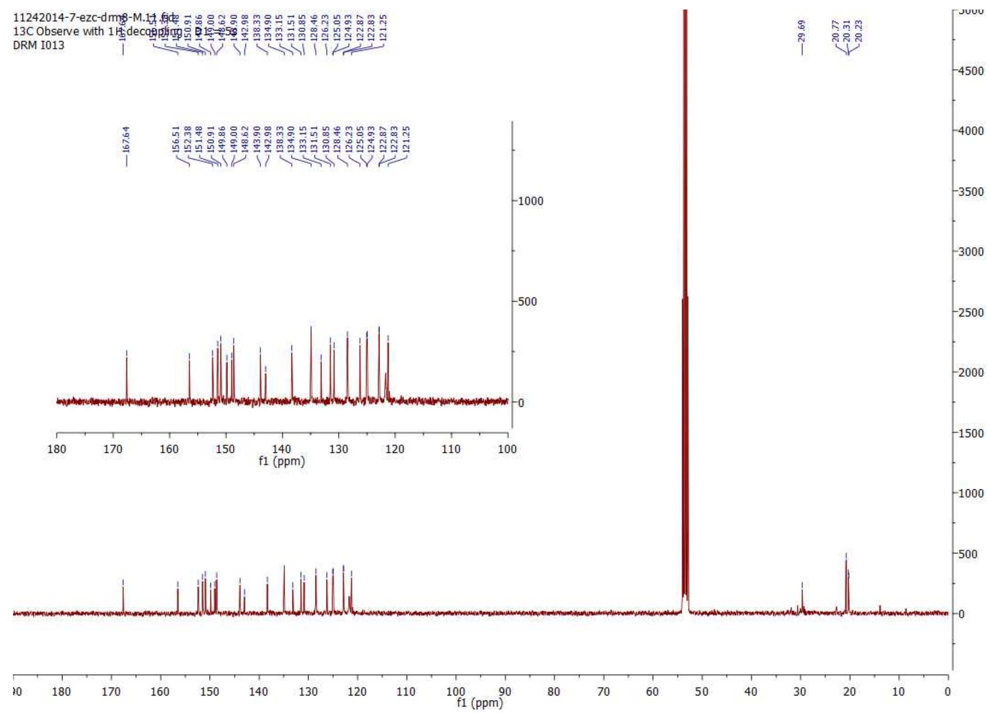

Figure **S4**. $^{13}$C NMR spectrum of ***rac*-, Δ– and Λ-[Ir(mesppy)$_2$(qpy)]PF$_6$** (***rac*-, Δ–** and **Λ-1-PF$_6$**) in $CD_2Cl_2$.

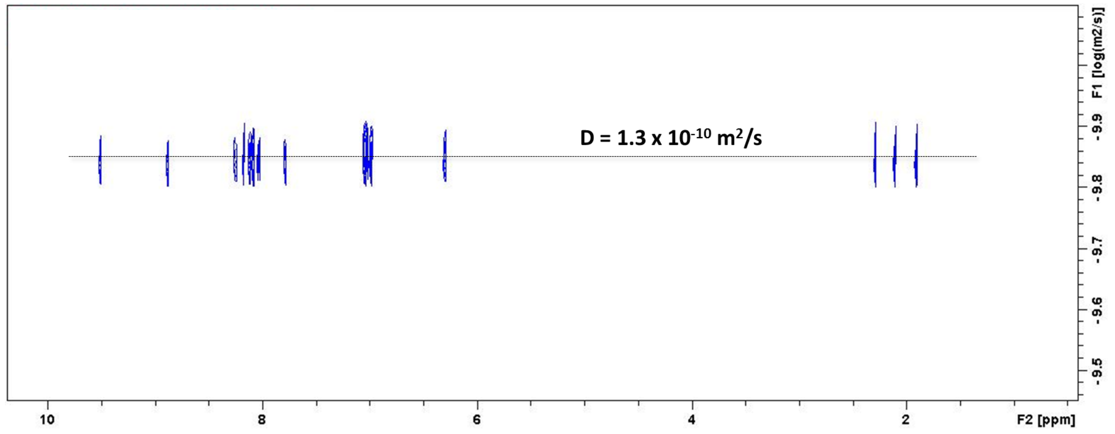


Figure **S5**. $^1$H DOSY NMR spectrum of ***rac*-[Ir(mesppy)$_2$(qpy)]PF$_6$** (***rac*-1-PF$_6$**) in DMSO-$d_6$ (D = 1.3 × $10^{-10}$ $m^2$/s).

**_rac_-, Δ– and Λ–Iridium(III)bis[2-phenyl-4-(2,4,6-trimethylphenyl)pyridinato]-4,4':2',2'':4'',4'''-quaterpyridine tetrafluoroborate, _rac_-, Δ– and Λ–[Ir(mesppy)$_2$(qpy)](BF$_4$)**.

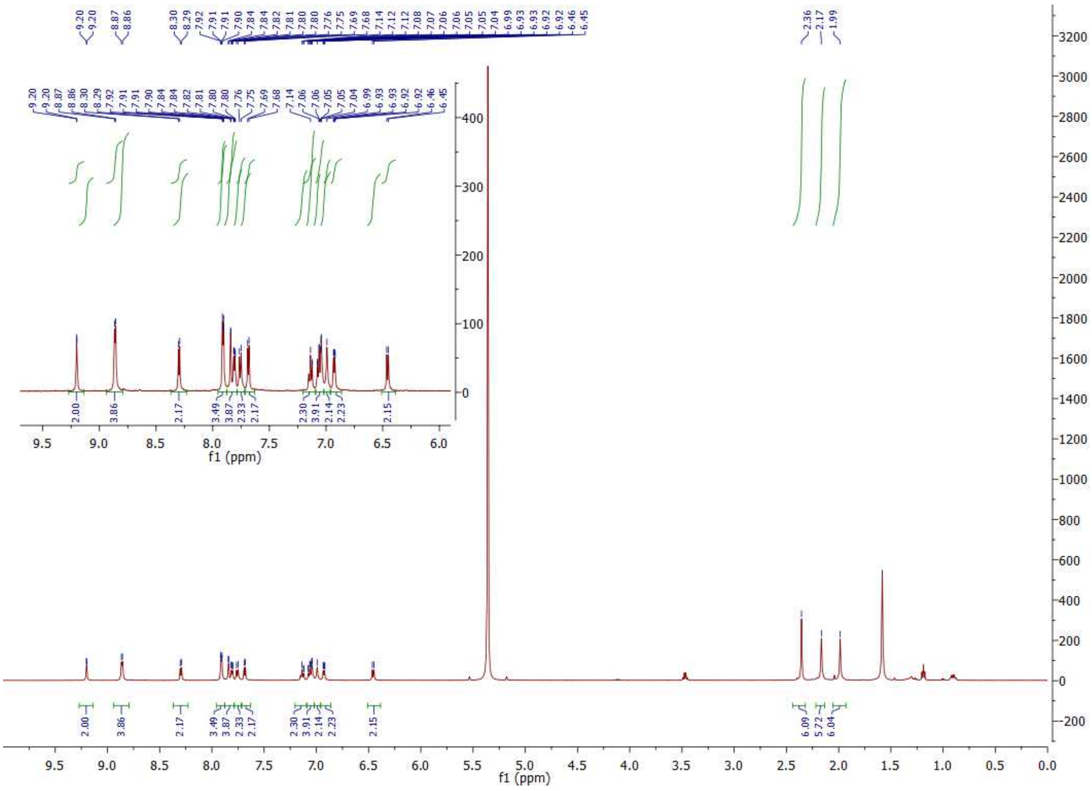


Figure **S6.** $^{1}$H NMR spectrum of ***rac*-, Λ-** and **Δ-[Ir(mesppy)$_2$(qpy)]BF$_4$** (***rac*-**, **Λ-** and **Δ-1-BF$_4$**) in $CD_2Cl_2$.

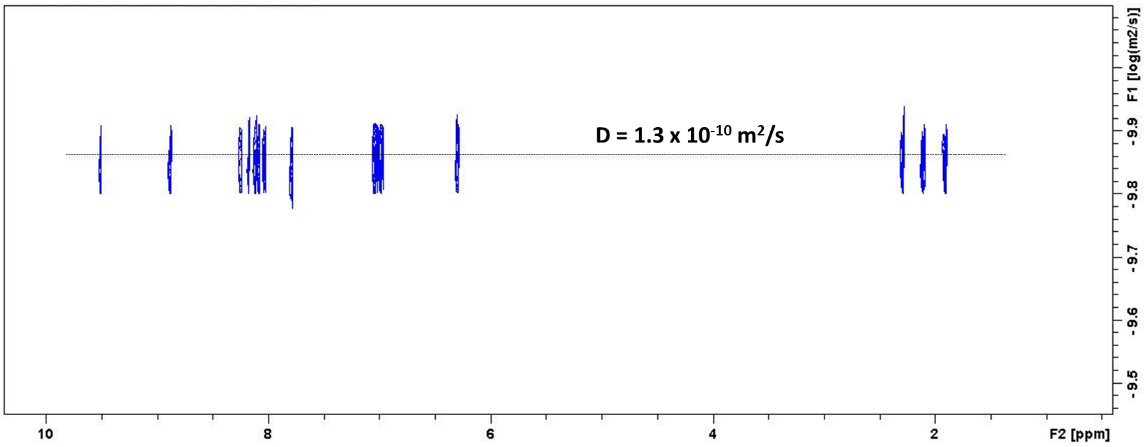


Figure **S7**. $^{1}$H DOSY NMR spectrum of **Δ-[Ir(mesppy)$_2$(qpy)]BF$_4$** (**Δ-1-BF$_4$**) in DMSO-$d_6$ (D = $1.3 \times 10^{-10}$ m$^2$/s).

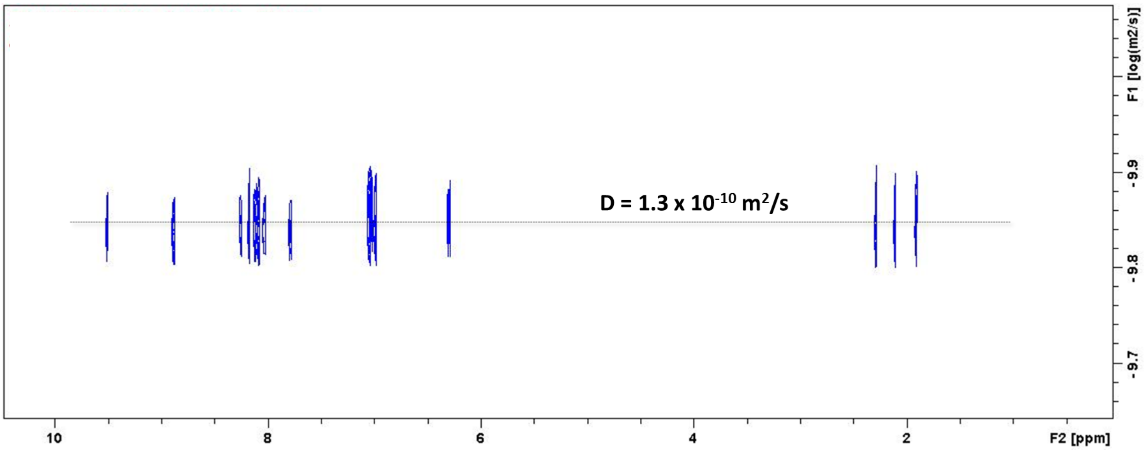


Figure **S8**. $^{1}$H DOSY NMR spectrum of **Λ-[Ir(mesppy)$_2$(qpy)]BF$_4$ (Λ-1-BF$_4$)** in DMSO-$d_6$ (D = 1.3 × $10^{-10}$ m$^2$/s).

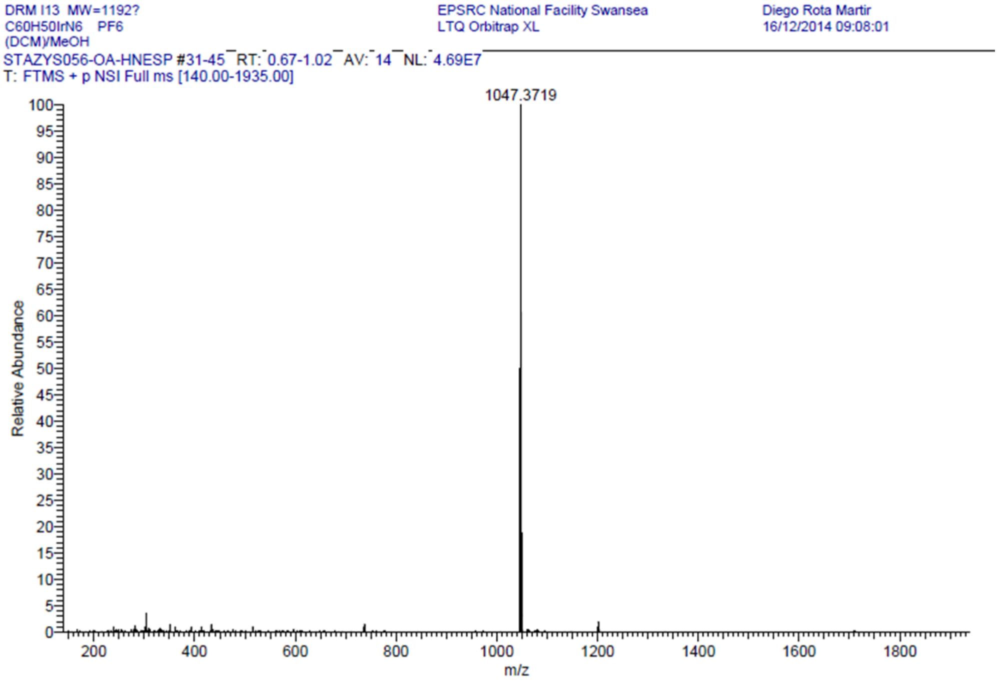


Figure **S9.** FTMS spectra of ***rac-***, **Λ-** and **Δ-[Ir(mesppy)$_2$(qpy)]BF$_4$ (*rac-*, Λ-** and **Δ−1−BF$_4$).**

**Δ−Iridium(III)bis[2-phenyl-4-(2,4,6-trimethylphenyl)pyridinato]-4,4':2',2'':4'',4'''-quaterpyridine hexafluoroantimonate, Δ−[Ir(mesppy)$_2$(qpy)](SbF$_6$).**

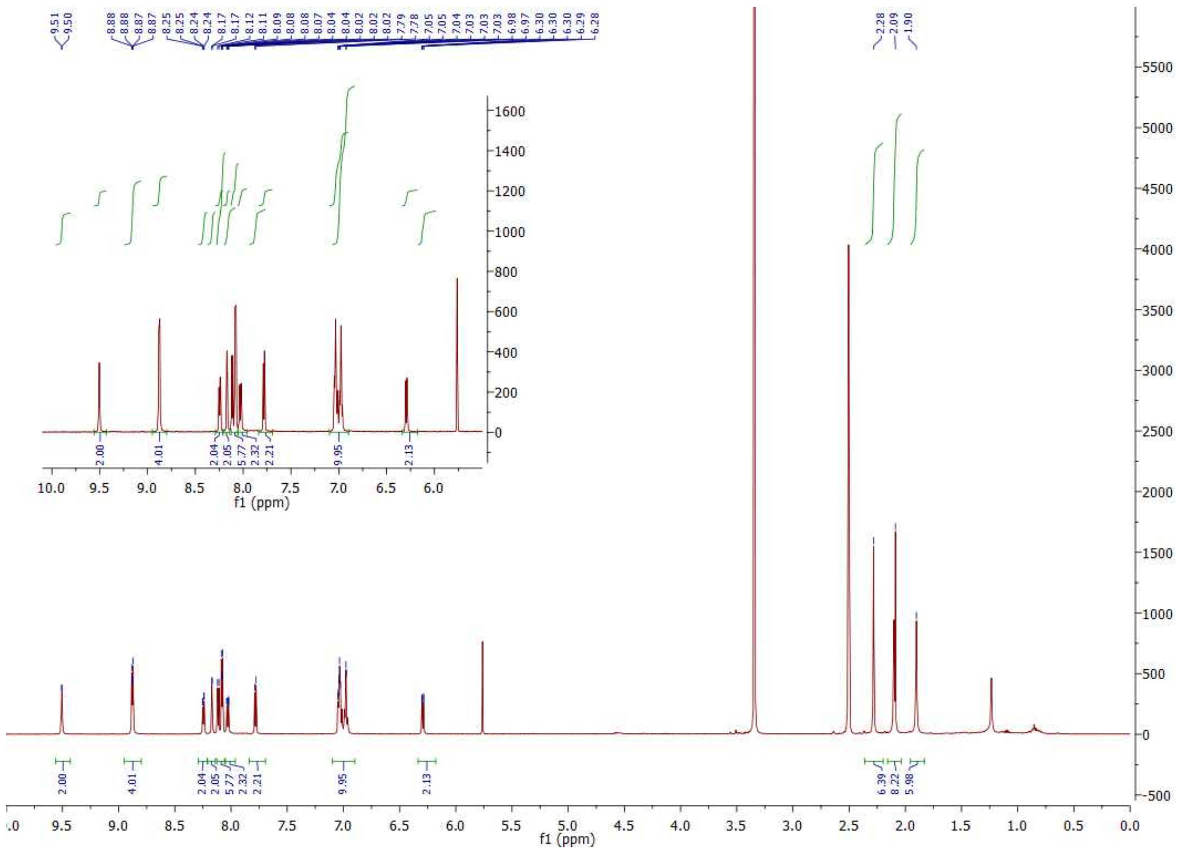

Figure **S10.** $^1$H NMR spectrum of **Δ-[Ir(mesppy)$_2$(qpy)]SbF$_6$** (**Δ-1-SbF$_6$**) in $CD_2Cl_2$.

***rac*-, Δ− and Λ−Iridium(III)bis[2-(2,4-difluoro)-phenyl-4-(2,4,6-trimethylphenyl)pyridinato]-4,4':2',2'':4'',4'''-quaterpyridine tetrafluoroborate, *rac*-, Δ− and Λ−[Ir(dFmesppy)$_2$(qpy)](BF$_4$).**

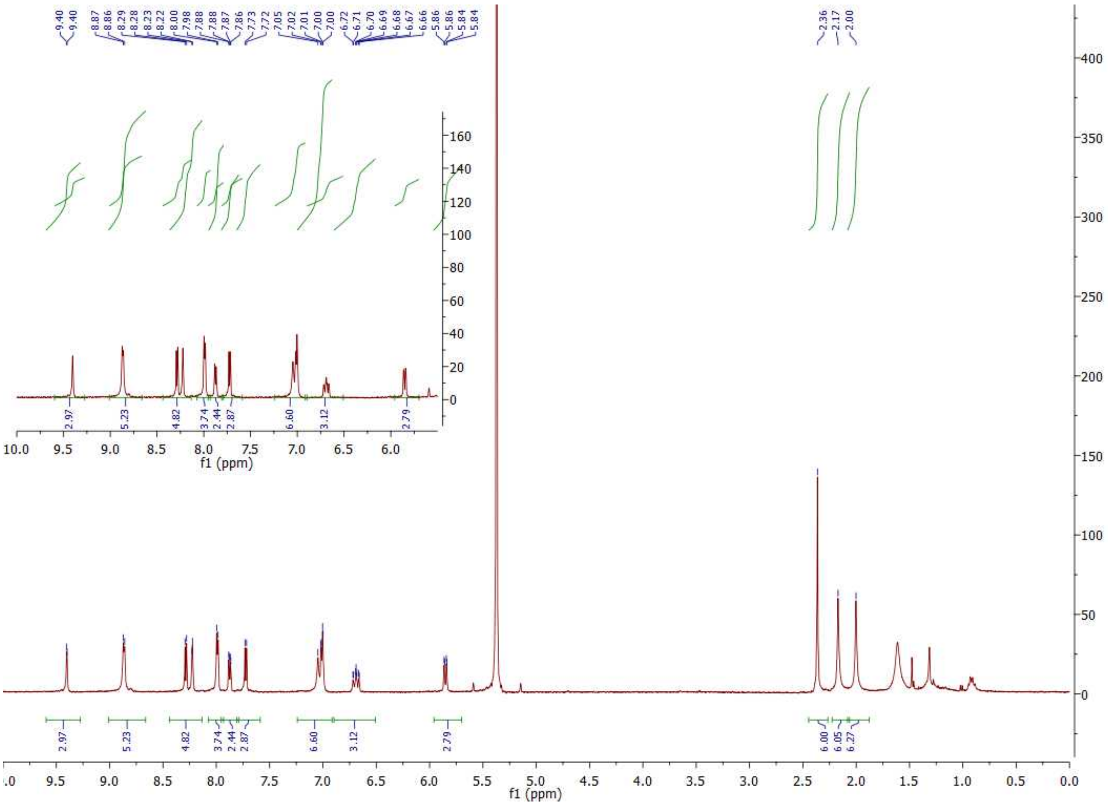


Figure **S11.** $^{1}$H NMR spectrum of ***rac-***, **Δ**- and **Λ-[Ir(dFmesppy)$_2$(qpy)]BF$_4$ (*rac-*, Λ**- and **Δ-2-BF$_4$)** in $CD_2Cl_2$.

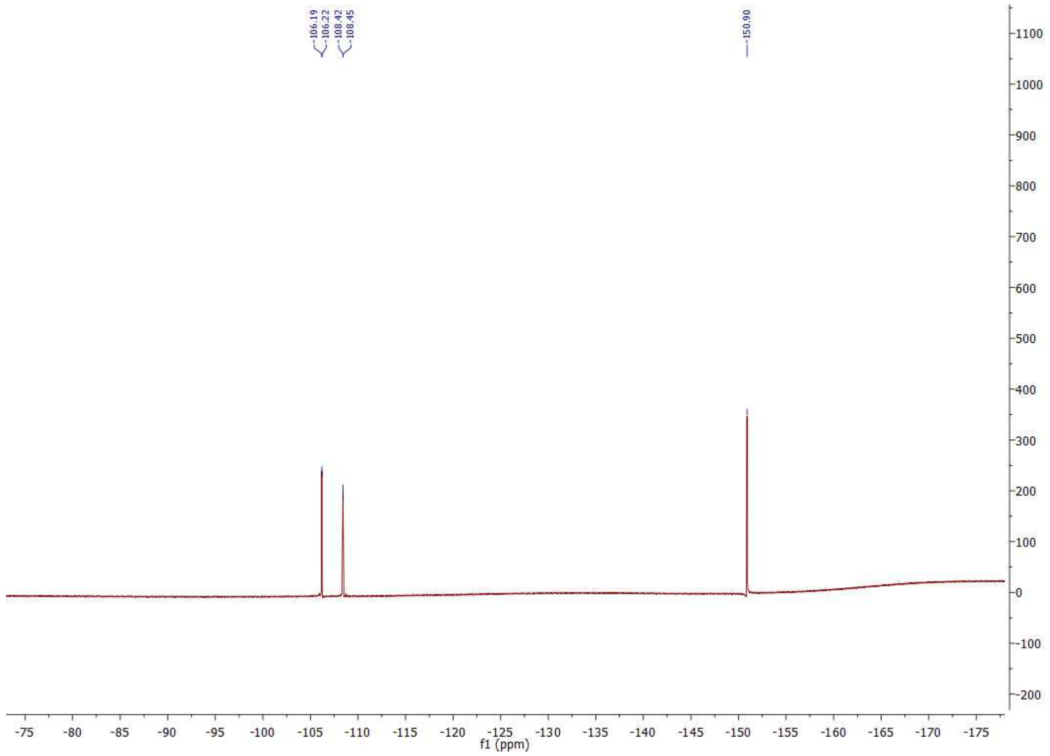


Figure **S12.** $^{19}$F NMR spectrum of ***rac-***, **Δ**- and **Λ-[Ir(mesppy)$_2$(qpy)]BF$_4$ (*rac-*, Λ**- and **Δ-2-BF$_4$)** in $CD_2Cl_2$.

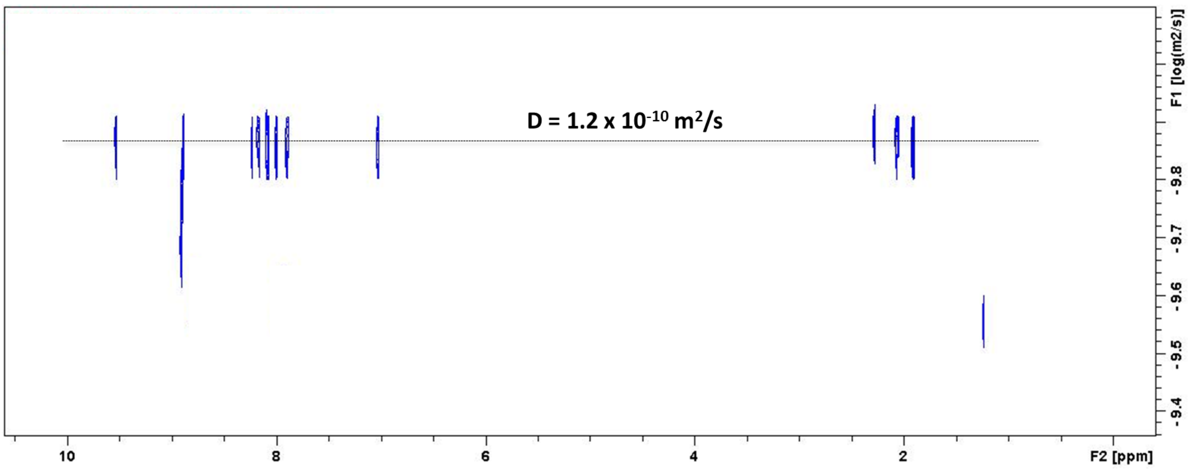


Figure **S13**. $^{1}$H DOSY NMR spectrum of ***rac*-[Ir(dFmesppy)$_2$(qpy)]BF$_4$** (***rac*-2-BF$_4$**) in DMSO-$d_6$ (D = 1.2 × $10^{-10}$ m$^2$/s).

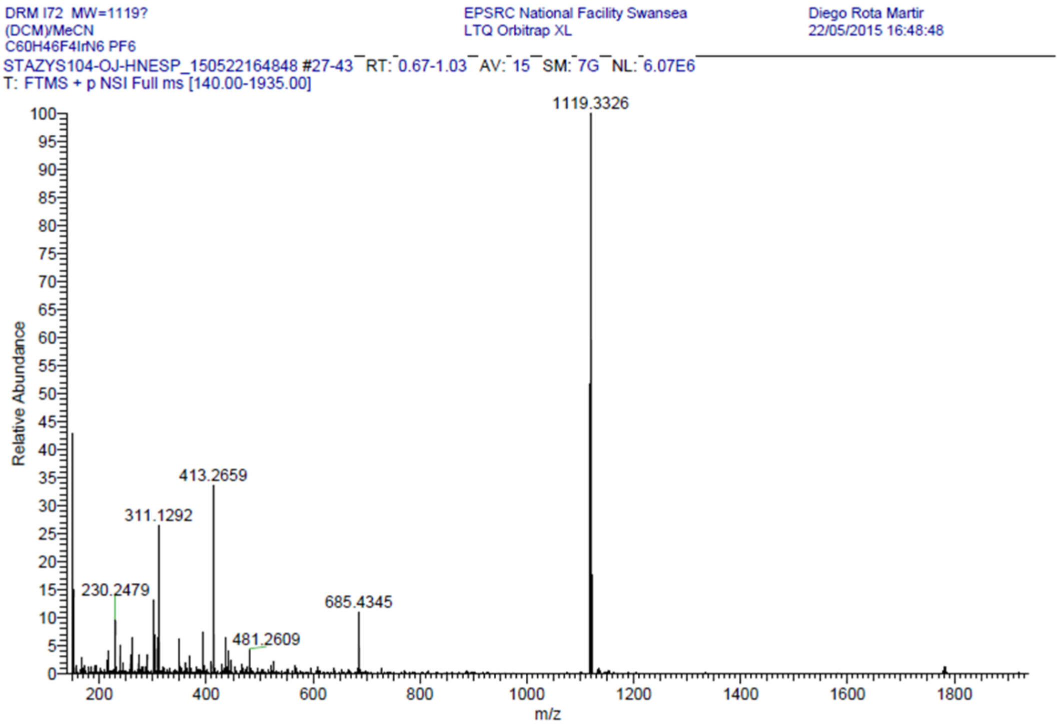


Figure **S14.** FTMS spectra of ***rac-***, **Λ**- and **Δ-[Ir(dFmesppy)$_2$(qpy)]BF$_4$** (***rac-*, Λ**- and **Δ–2–BF$_4$).**

***rac*-Tetrabutyl ammonium bis(cyanide)-bis[2-(2,4-difluoro)-phenylpyridinato]-iridium(III), *rac*-NBu$_4$[Ir(dFppy)$_2$(CN)$_2$] (IrCN).**

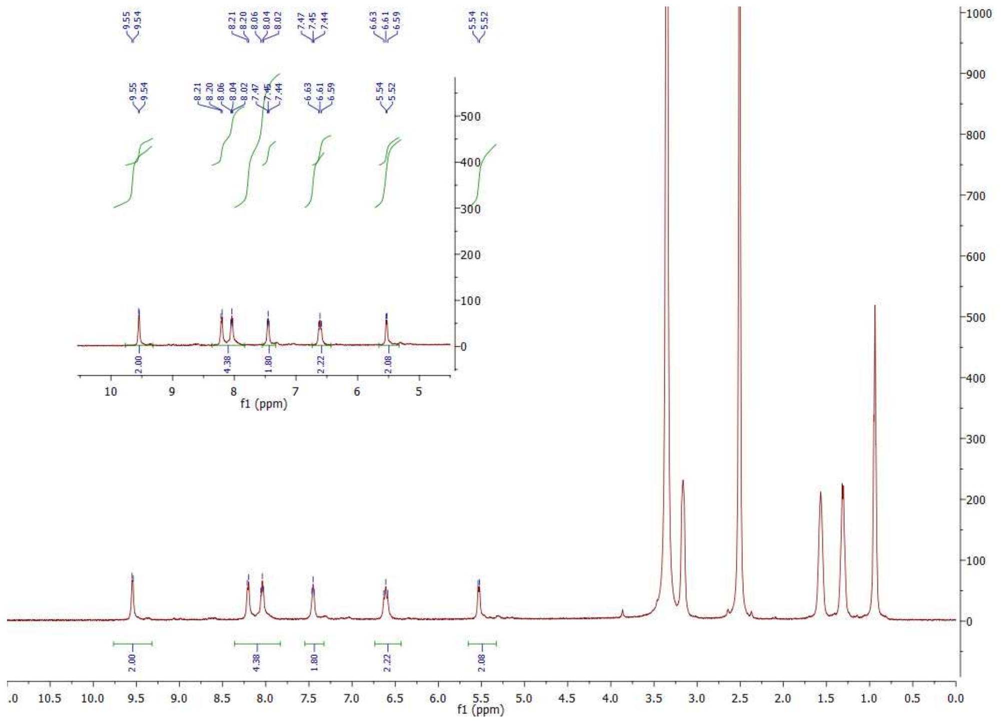

Figure **S15**.$^{1}$H NMR spectrum of **NBu$_4$[Ir(dFppy)$_2$(CN)$_2$] (IrCN)** in DMSO-d$_6$.

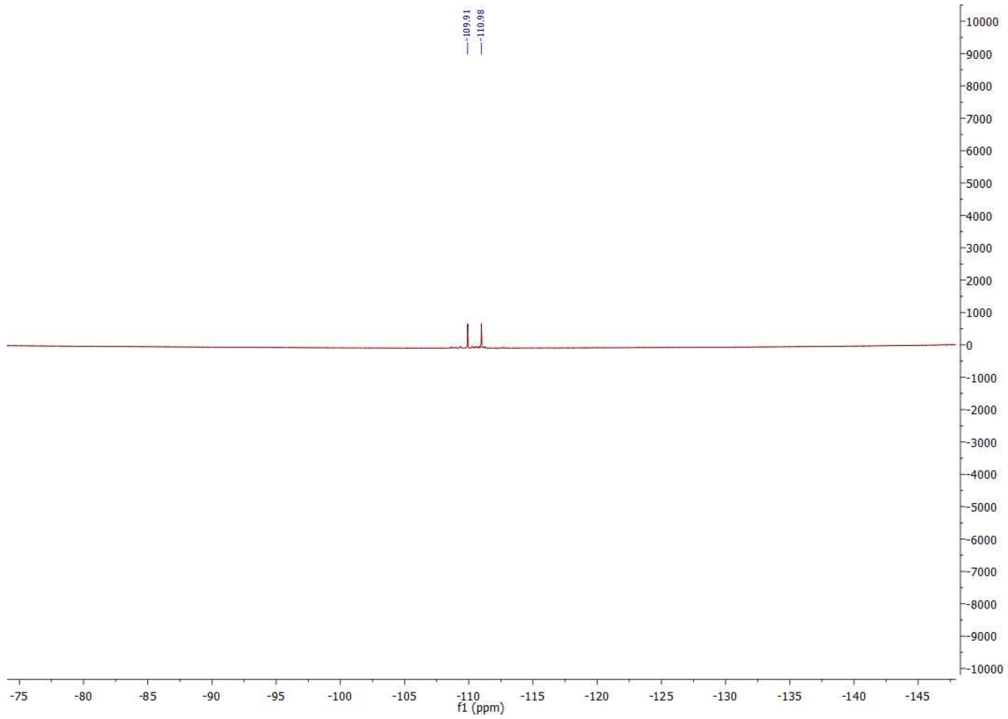

Figure **S16**.$^{19}$F NMR spectrum of **NBu$_4$[Ir(dFppy)$_2$(CN)$_2$] (IrCN)** in DMSO-d$_6$.

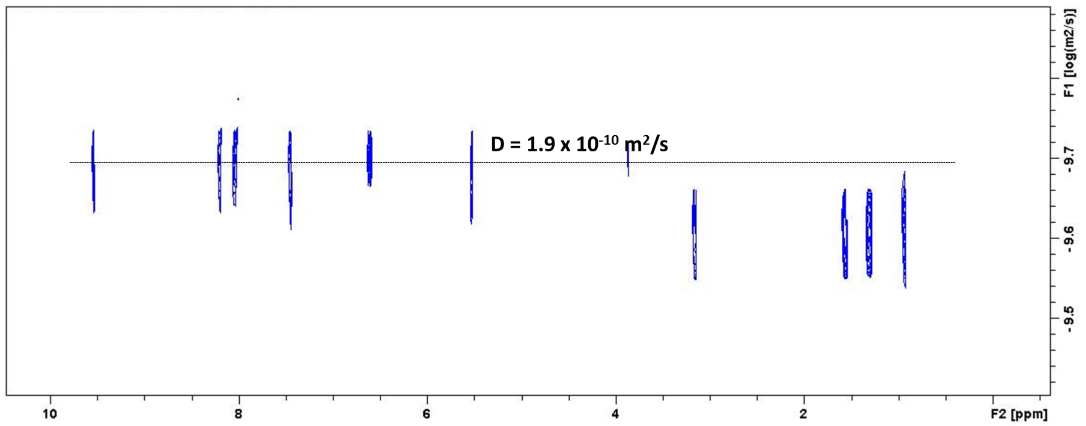


Figure **S17**. $^{1}$H DOSY NMR spectrum of ***rac*-NBu$_4$[Ir(dFppy)$_2$(CN)$_2$] (IrCN)** in DMSO-$d_6$ (D = $1.9 \times 10^{-10}$ m$^2$/s).

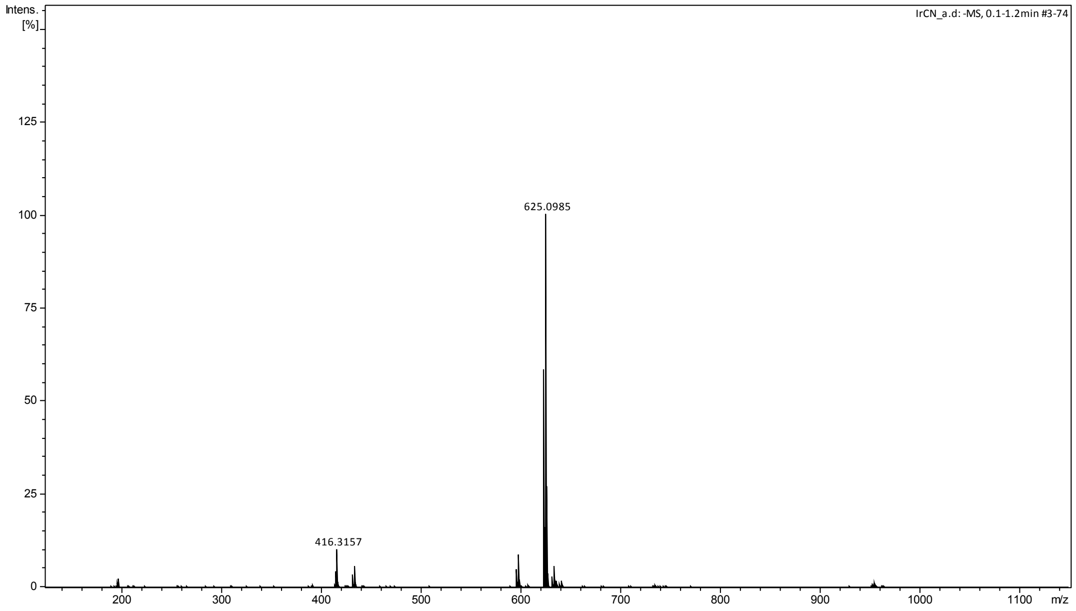


Figure **S18.** ESI-MS spectra of ***rac*-NBu$_4$[Ir(dFppy)$_2$(CN)$_2$] (IrCN).**

***rac*-Iridium(III)bis[2-(2,4-difluoro)-phenylpyridinato]-4,4'-dimethyl-2,2'-bypiridine hexafluorophosphate, [Ir(dFppy)$_2$(dmbpy)](PF$_6$) (Irdmbpy).**

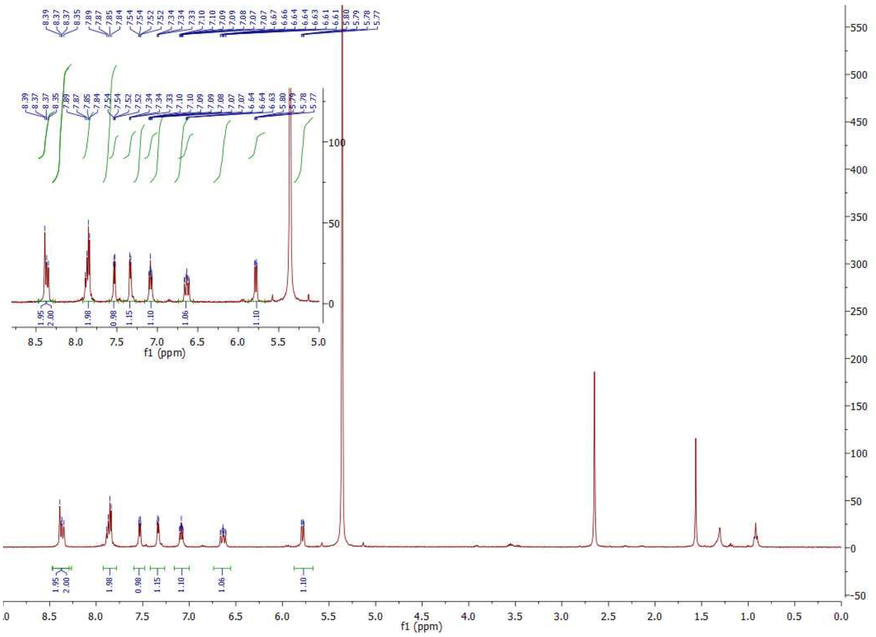


Figure **S19**.$^{1}$H NMR spectrum of ***rac*-[Ir(dFppy)$_2$(dmbpy)]PF$_6$ (Irdmbpy)** in $CD_2Cl_2$.

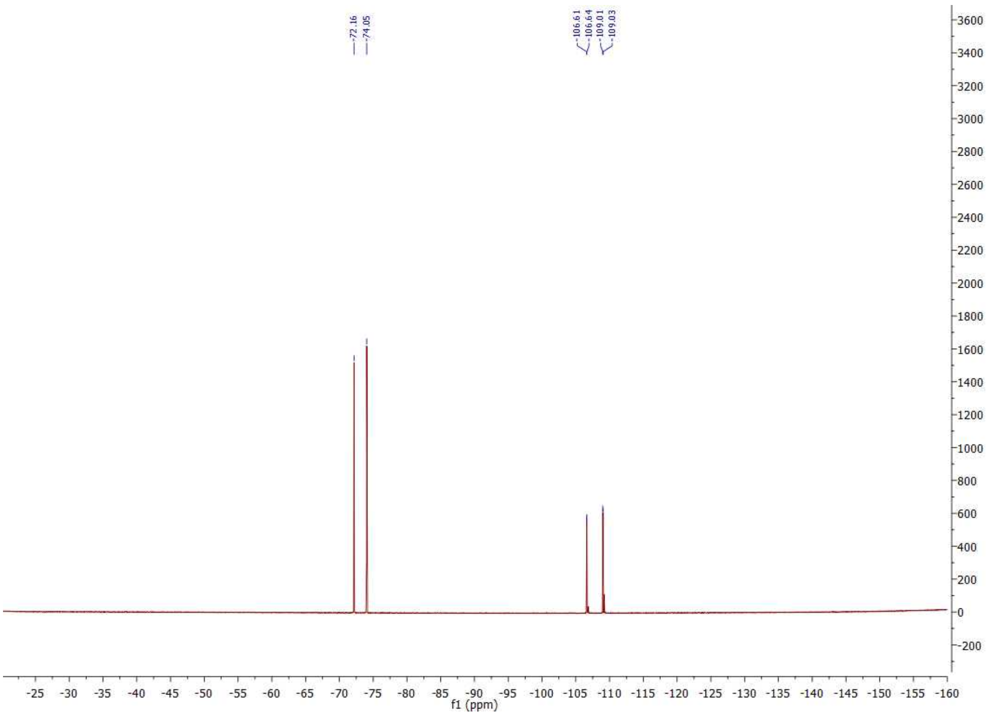


Figure **S20**.$^{19}$F NMR spectrum of ***rac*-[Ir(dFppy)$_2$(dmbpy)]PF$_6$ (Irdmbpy)** in $CD_2Cl_2$.

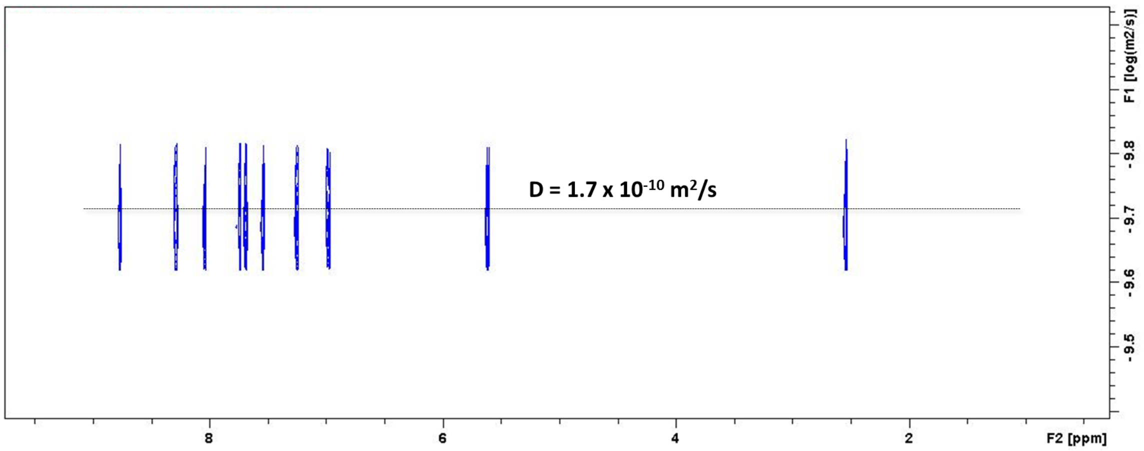


Figure **S21**. $^{1}$H DOSY NMR spectrum of ***rac*-[Ir(dFppy)$_2$(dmbpy)]PF$_6$ (Irdmbpy)** in DMSO-$d_6$ (D = 1.7 × 10$^{-10}$ m$^2$/s).

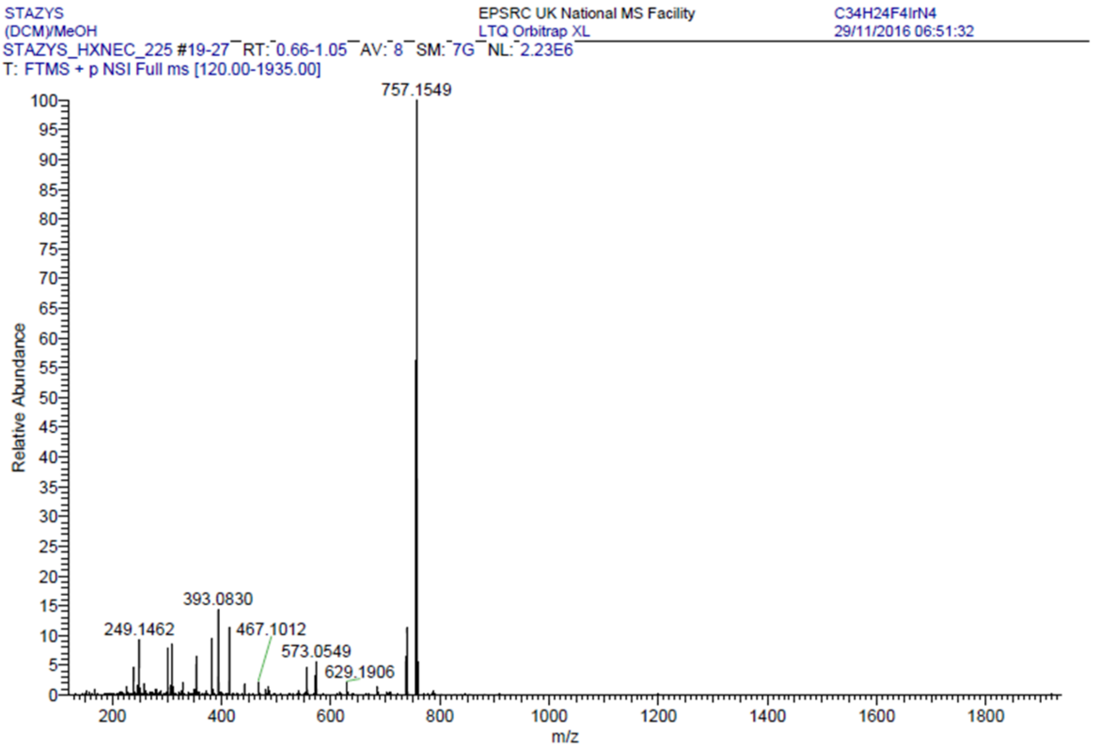


Figure **S22.** FTMS spectra of ***rac*-[Ir(dFppy)$_2$(dmbpy)]PF$_6$ (Irdmbpy).**

# NMR Characterisation of *rac*-, $\Delta_8$-, $\Lambda_8$-[$1_8Pd_4$]($BF_4$)$_{16}$, (*rac*-, $\Delta_8$-, $\Lambda_8$-C1-$BF_4$) Cages.

### NMR Spectra of $\Delta_8$- and $\Lambda_8$-[$1_8Pd_4$]($BF_4$)$_{16}$ Cages ($\Delta_8$- and $\Lambda_8$-C1-$BF_4$).

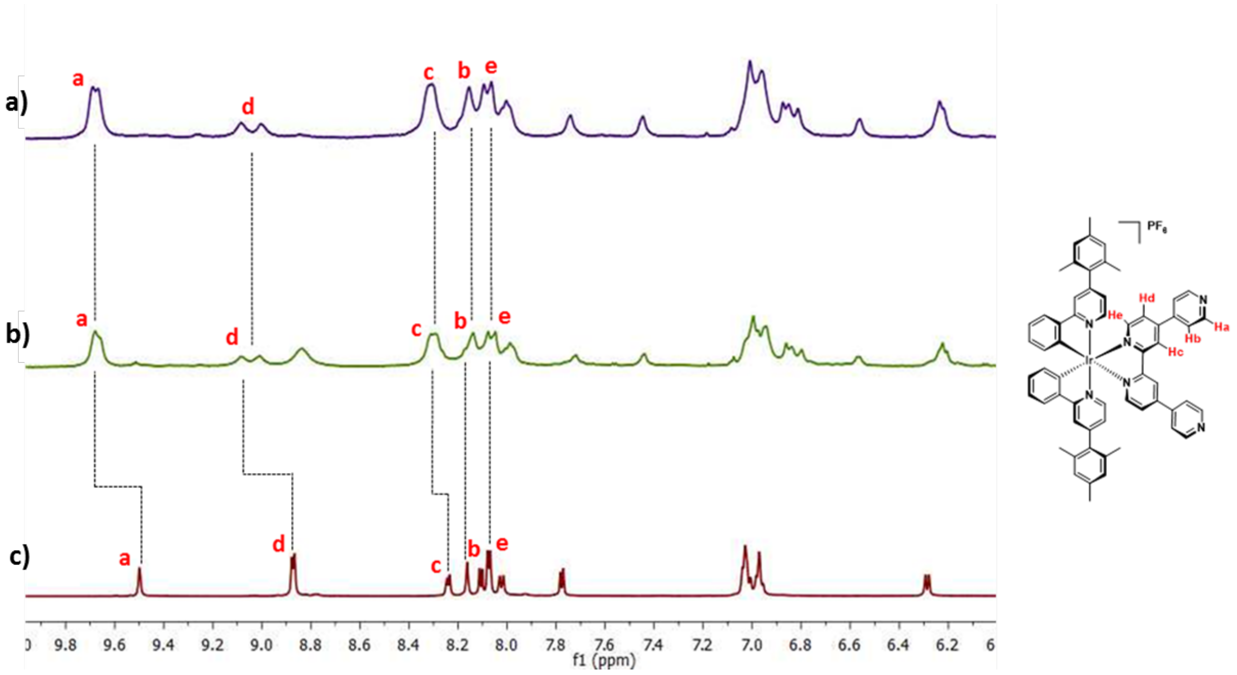


Figure **S23.** $^1$H NMR spectrum of **a)** **$\Lambda_8$–C1-$BF_4$**; **b)** **$\Delta_8$–C1-$BF_4$** and **c)** *rac*--**[Ir(mesppy)$_2$(qpy)]$BF_4$** (***rac*-1-$BF_4$**) collected in DMSO-$d_6$ at 298 K.

### $^1$H NMR of cages $\Delta_8$-[$1_8Pd_4$]$^{16+}$, ($\Delta_8$-C1) with $BF_4^-$, $PF_6^-$ and $SbF_6^-$ as counterions.

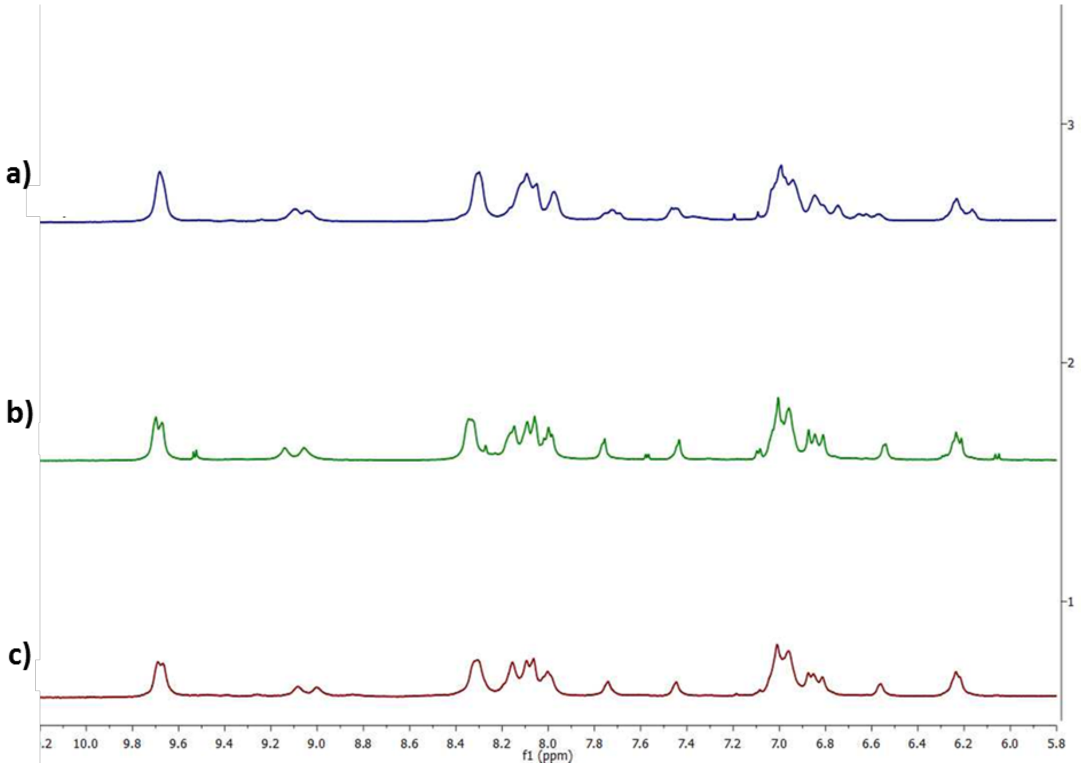


Figure **S24.** $^1$H NMR spectra of **a) $\Delta_8$–C1-SbF$_6$**; **b) $\Delta_8$–C1-PF$_6$** and **c) $\Delta_8$-C1-BF$_4$** collected in DMSO-$d_6$ at 298 K.

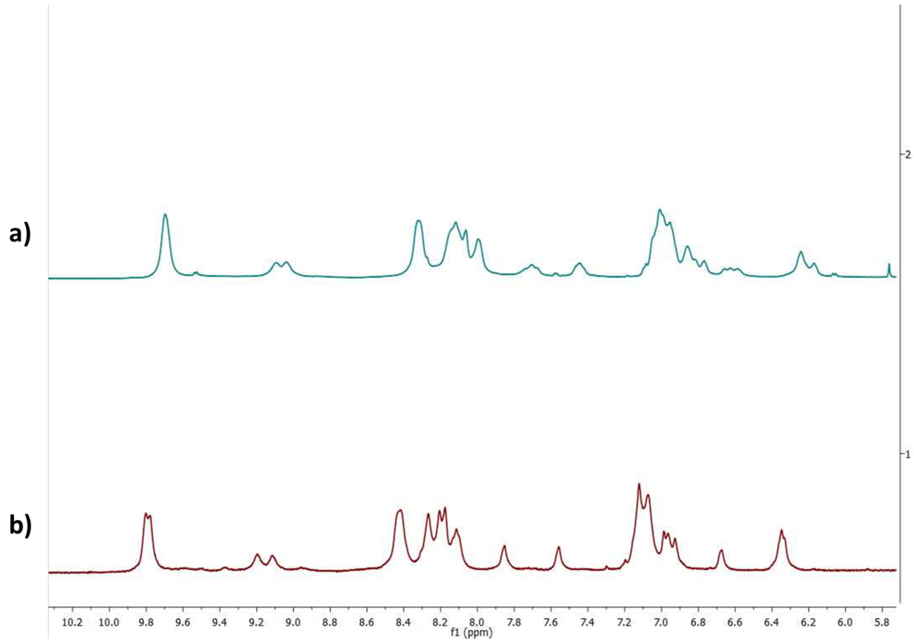


Figure **S25.** $^1$H NMR spectra of **a) *rac*–C1-PF$_6$** and **b) *rac*–C1-BF$_4$** collected in DMSO-$d_6$ at 298 K.

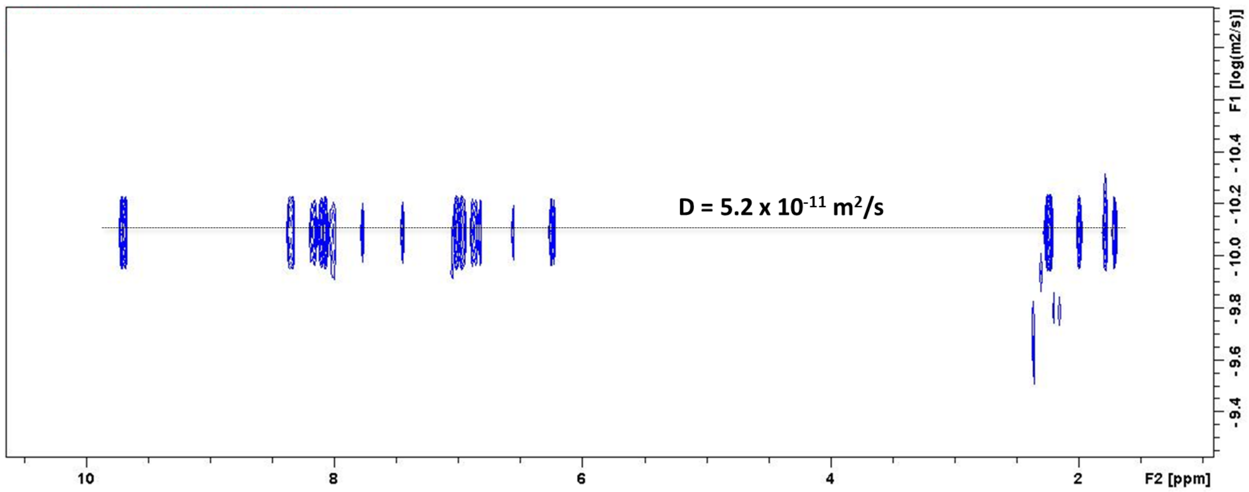


Figure **S26**. $^{1}$H DOSY NMR spectrum of ***rac*-C1-BF$_4$** in DMSO-$d_6$ at 298 K (D = 5.2 × $10^{-11}$ $m^2$/s).

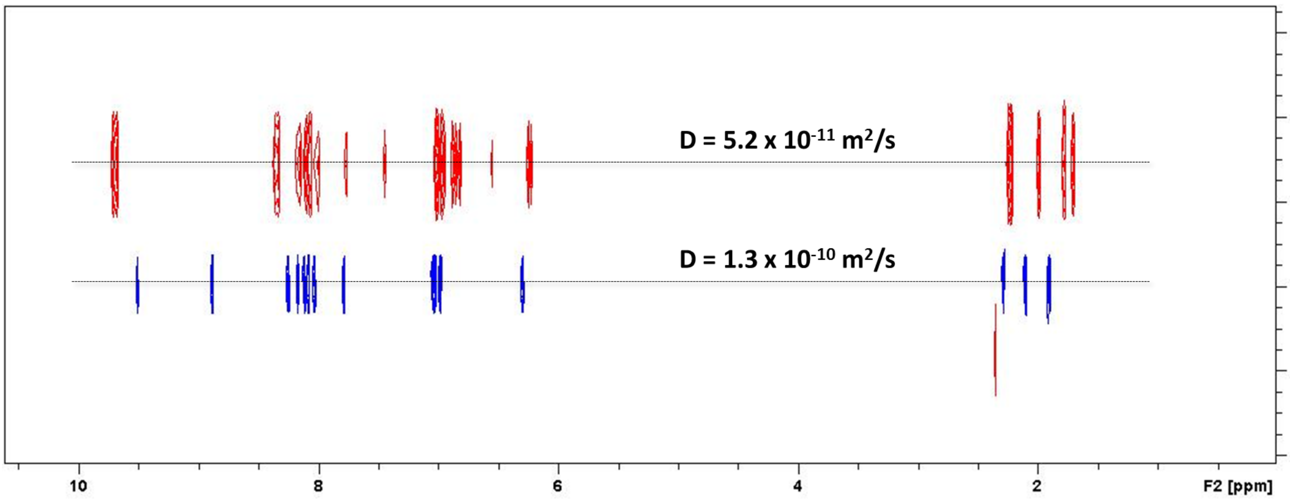


Figure **S27**. Comparison between the $^{1}$H DOSY NMR spectra of **$\Lambda_8$-C1-BF$_4$** (in red), and of **Λ−1** (in blue) in DMSO-$d_6$ at 298 K.

## NMR Spectra of *rac*-, $\Delta_8$- and $\Lambda_8$-[$2_8$Pd$_4$](BF$_4$)$_{16}$, (*rac*-, $\Delta_8$- and $\Lambda_8$-C2-BF$_4$).

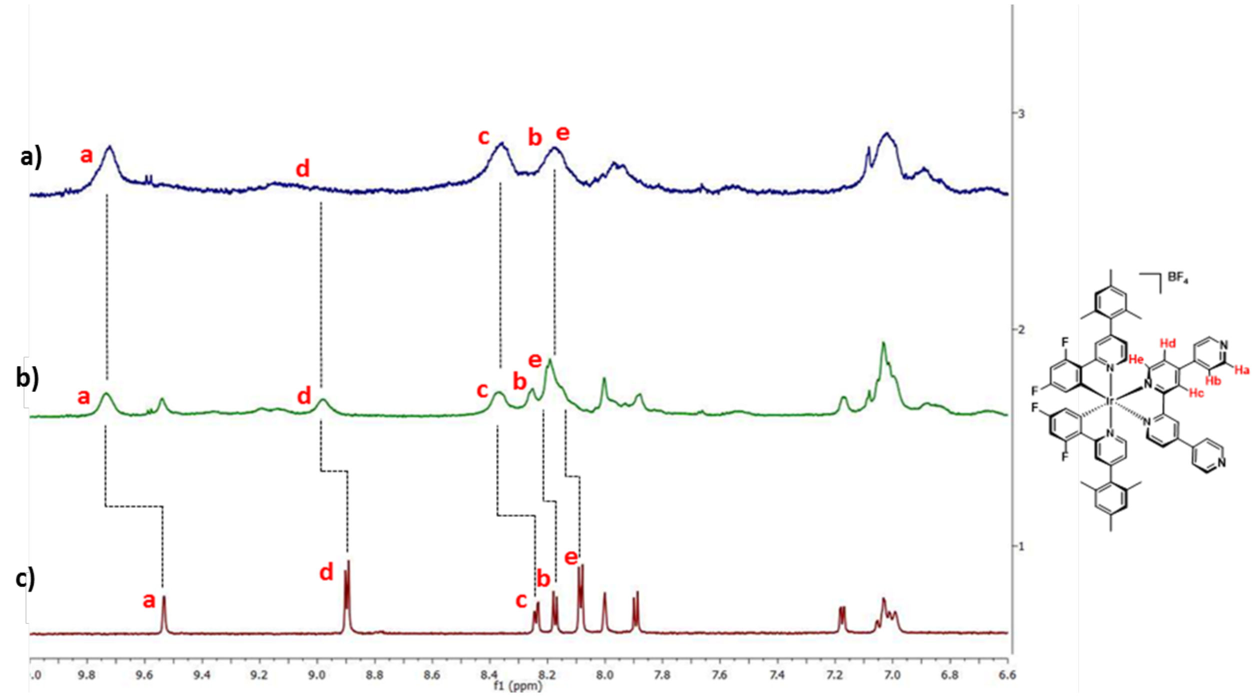


Figure **S28.** $^{1}$H NMR spectra of **a)** $\boldsymbol{\Lambda_8}$**–C2-BF$_4$**; **b)** $\boldsymbol{\Delta_8}$**–C2-BF$_4$** and **c)** ***rac*****--[Ir(dFmesppy)$_2$(qpy)]BF$_4$** (***rac*****-2-BF$_4$**) collected in DMSO-$d_6$ at 298 K.

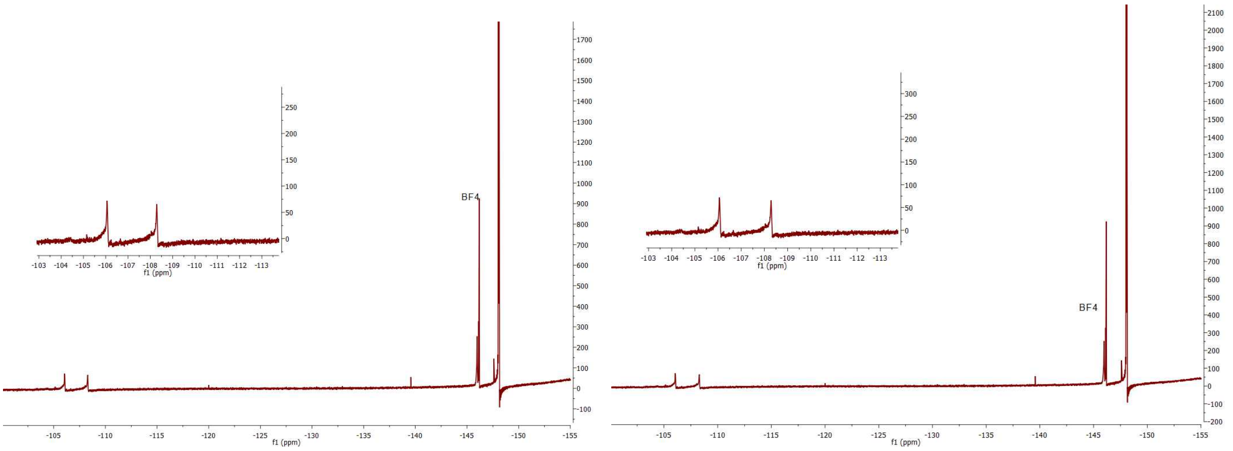


Figure **S29.** $^{19}$F NMR spectra of **a)** ***rac*****–C2-BF$_4$** (left); and **b)** $\boldsymbol{\Lambda_8}$**–C2-BF$_4$** (right) collected in DMSO-$d_6$ at 298 K.

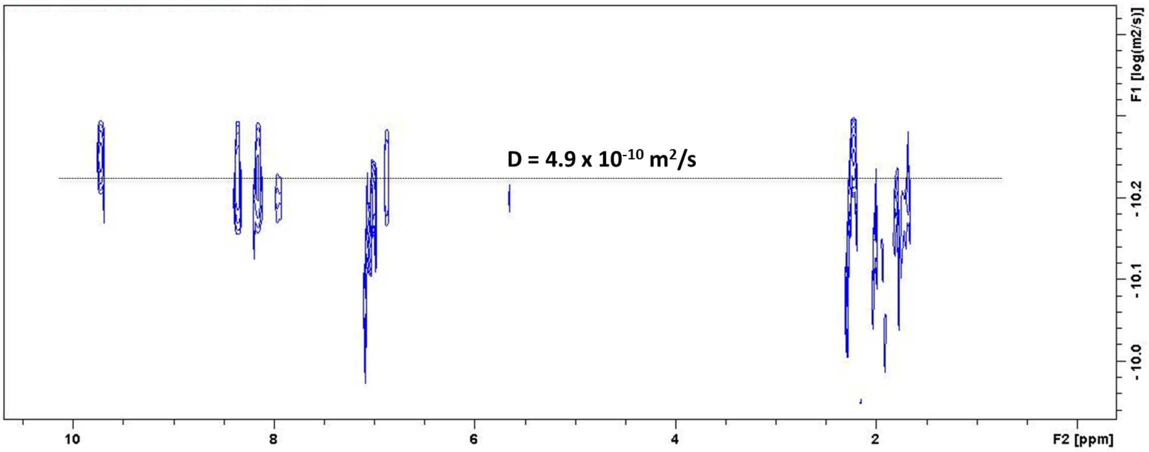


Figure **S30**. $^{1}$H DOSY NMR spectrum of ***rac*-C2-BF$_4$** in DMSO-$d_6$ at 298 K (D = 4.9 × $10^{-11}$ $m^2$/s).

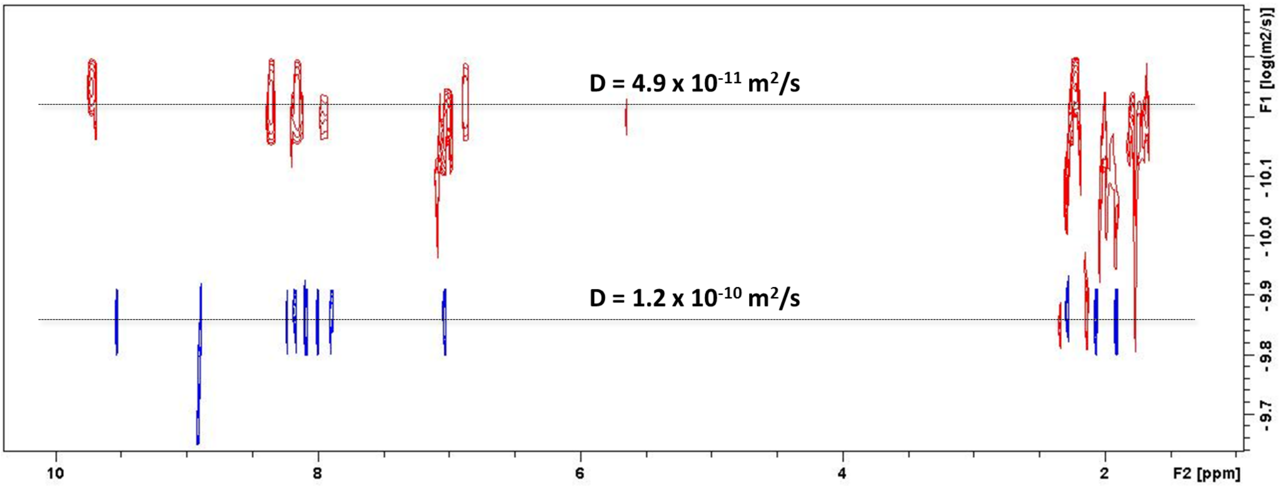


Figure **S31**. Comparison between the $^{1}$H DOSY NMR spectra of **Λ$_8$-C2-BF$_4$** (in red) and of **Λ−2** (in blue) in DMSO-$d_6$ at 298 K.

# Calculation of Hydrodynamic Radii ($r_s$) of *rac*-, Δ- and Λ−1, *rac*-, Δ−, Λ−2 *rac*-, Δ$_8$- and Λ$_8$-C1 and *rac*-, Δ$_8$- and Λ$_8$-C2.

**Table S1.** Comparison of diffusion coefficients (D, $m^2\ s^{-1}$) of ligands and cages obtained by $^1$H DOSY NMR (500 MHz, DMSO-$d_6$).

| **metalloligand** | **D metalloligand ($m^2 s^{-1}$)** | **cages** | **D cages ($m^2 s^{-1}$)** | **$r_s$ metalloligands (nm)** | **$r_s$ cages (nm)** |
|---|---|---|---|---|---|
| ***rac*-, Δ-** and **Λ-1** | $1.3 \times 10^{-10}$ | ***rac*-, Δ-** and **Λ-1** | $5.2 \times 10^{-11}$ | 0.83 | 1.98 |
| ***rac*-, Δ-** and **Λ-2** | $1.2 \times 10^{-10}$ | ***rac*-, Δ-** and **Λ-2** | $4.9 \times 10^{-11}$ | 0.90 | 2.00 |

In diffusion experiments, the molecular size of the metalloligands and metallocages are estimated by the Stokes Einstein equation:

$$r_s = \frac{k_b \cdot \mathrm{T}}{6\pi \cdot \eta \cdot D}$$

With $r_s$ = hydrodynamic or Stokes radii of the metalloligands or metallocages, which are assumed to exhibit a spherical shape, $k_b$ = Boltzmann constant, T = temperature (298 K), η = viscosity of DMSO solution (1.99 mPa • s) and D = diffusion coefficient, obtained here by $^1$H DOSY NMR.

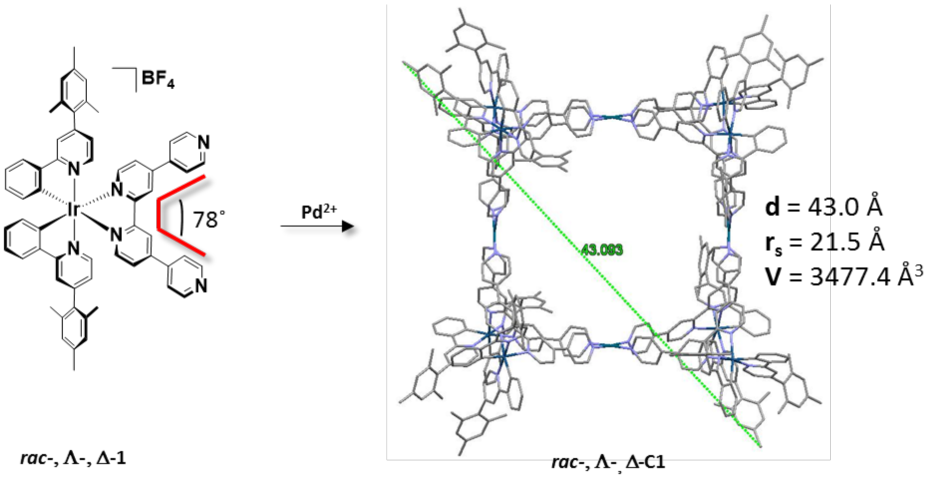


*rac*-, Λ-, Δ-1 *rac*-, Λ-, Δ-C1

Figure **S32**. Illustration of the qpy coordinating vector of **1** and of the diameter around the metallamacrocyclic core across long axes of **C1** from one mesityl on one 'end' to the opposite mesityl on the other 'end'.

# ESI-MS Spectra of *rac*-, $\Delta_8$- and $\Lambda_8$-[$1_8Pd_4$]$^{16+}$, (*rac*-, Δ- and Λ-C1) Cages.

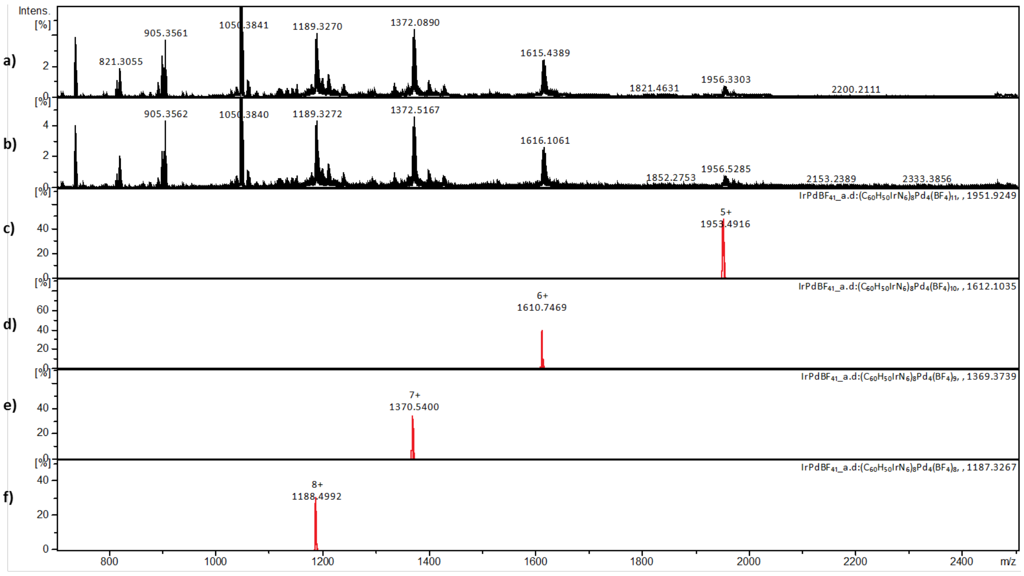


Figure **S33**. ESI-MS spectra of: **a) $\Delta_8$-C1-BF$_4$** and **b) $\Lambda_8$-C1-BF$_4$**. Simulated MS spectra of: **c)** [**C1**-$(BF_4)_5$]$^{5+}$; **d)** [**C1**-$(BF_4)_6$]$^{6+}$; **e)** [**C1**-$(BF_4)_7$]$^{7+}$ and **f)** [**C1**-$(BF_4)_8$]$^{8+}$.

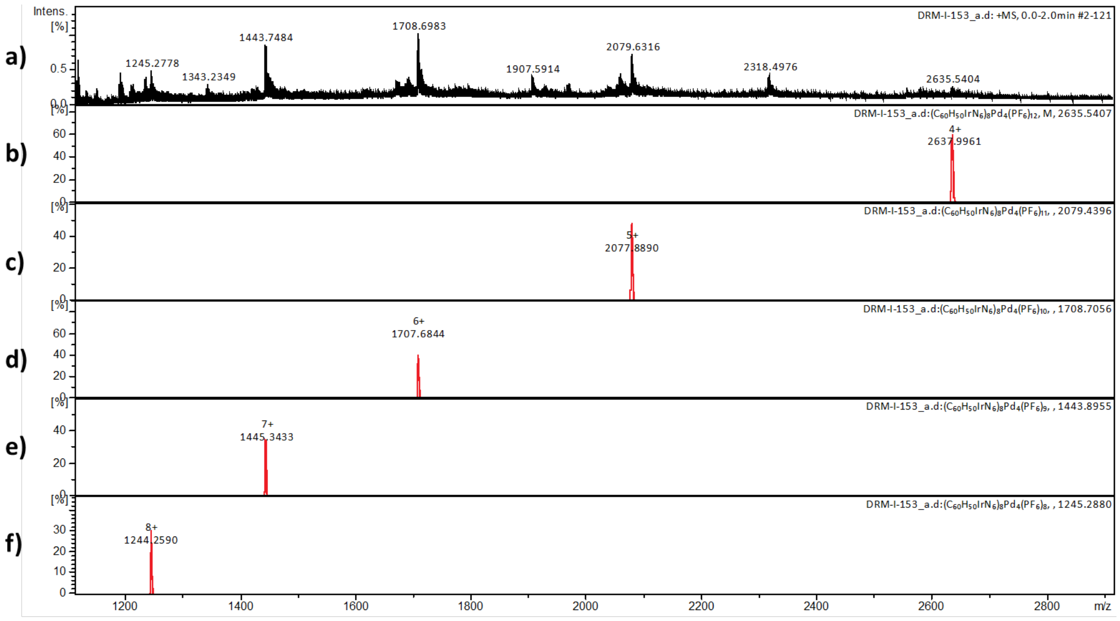


Figure **S34**. **a)** ESI-MS spectra of $\mathbf{\Lambda_8}$**-C1-PF$_6$**. Simulated MS spectra of: **b)** [**C1**-$(PF_6)_4]^{4+}$; **c)** [**C1**-$(PF_6)_5]^{5+}$; **d)** [**C1**-$(PF_6)_6]^{6+}$; **e)** [**C1**-$(PF_6)_7]^{7+}$ and **f)** [**C1**-$(PF_6)_8]^{8+}$.

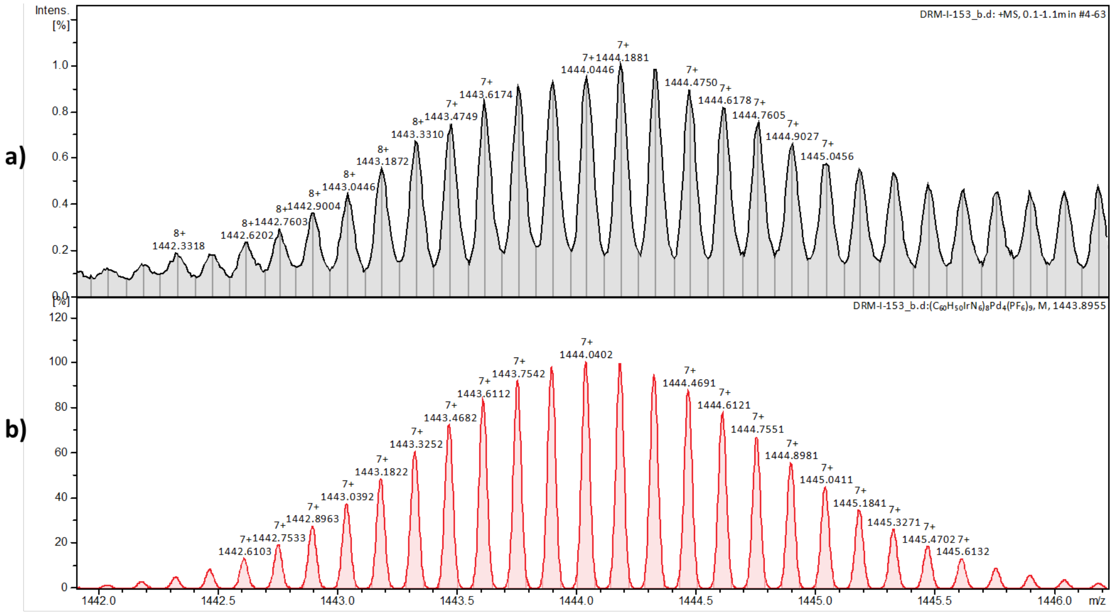


Figure **S35**. **a)** ESI-MS spectra of the [**C1**-$(PF_6)_7]^{7+}$ of $\mathbf{\Delta_8}$**-C1-PF$_6$; b)** Simulation of isotopic distribution pattern of [**C1**-$(PF_6)_7]^{7+}$.

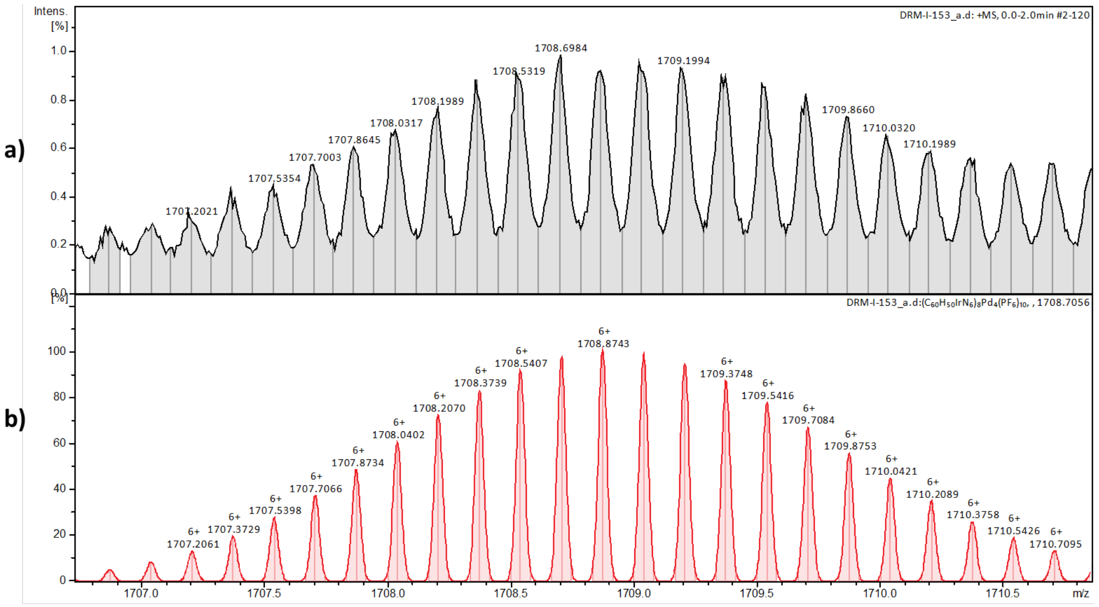


Figure **S36**. **a)** ESI-MS spectra of the [**C1**-($PF_6^-$)$_6$]$^{6+}$ of **Δ$_8$-C1-PF$_6$; b)** Simulation of isotopic distribution pattern of [**C1**-($PF_6$)$_6$]$^{6+}$.

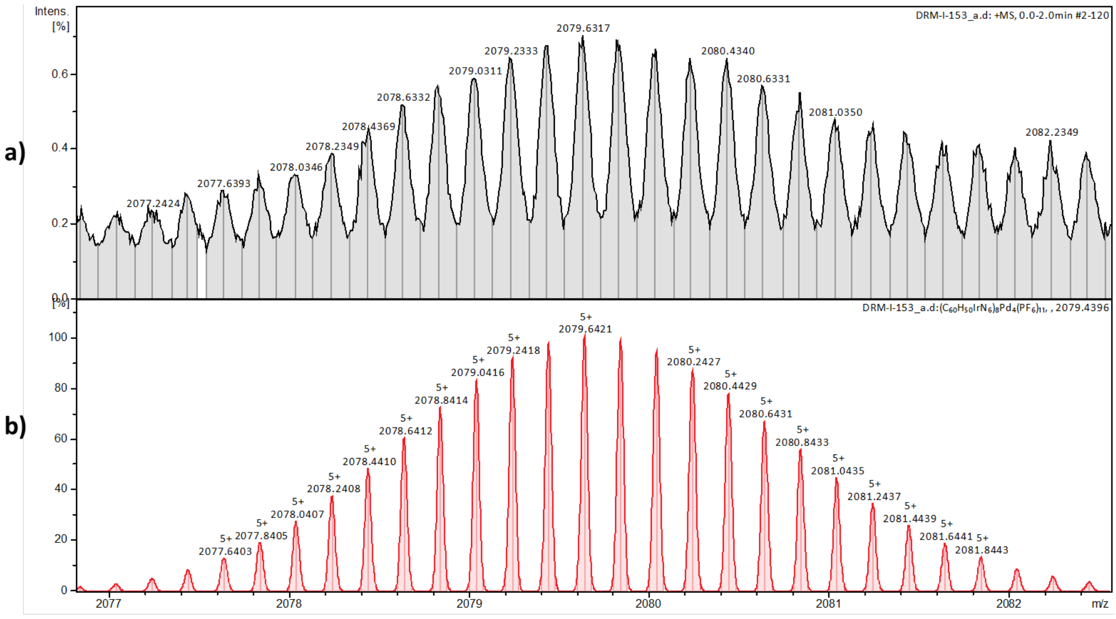


Figure **S37**. **a)** ESI-MS spectra of the [**C1**-($PF_6$)$_5$]$^{5+}$ of **Δ$_8$-C1-PF$_6$, b)** Simulation of isotopic distribution pattern of [**C1**-($PF_6$)$_5$]$^{5+}$.

# ESI-MS of *rac*-, $\Delta_8$- and $\Lambda_8$-[$2_8Pd_4$]$(BF_4)_{16}$, (*rac*-, $\Delta$- and $\Lambda$-C2).

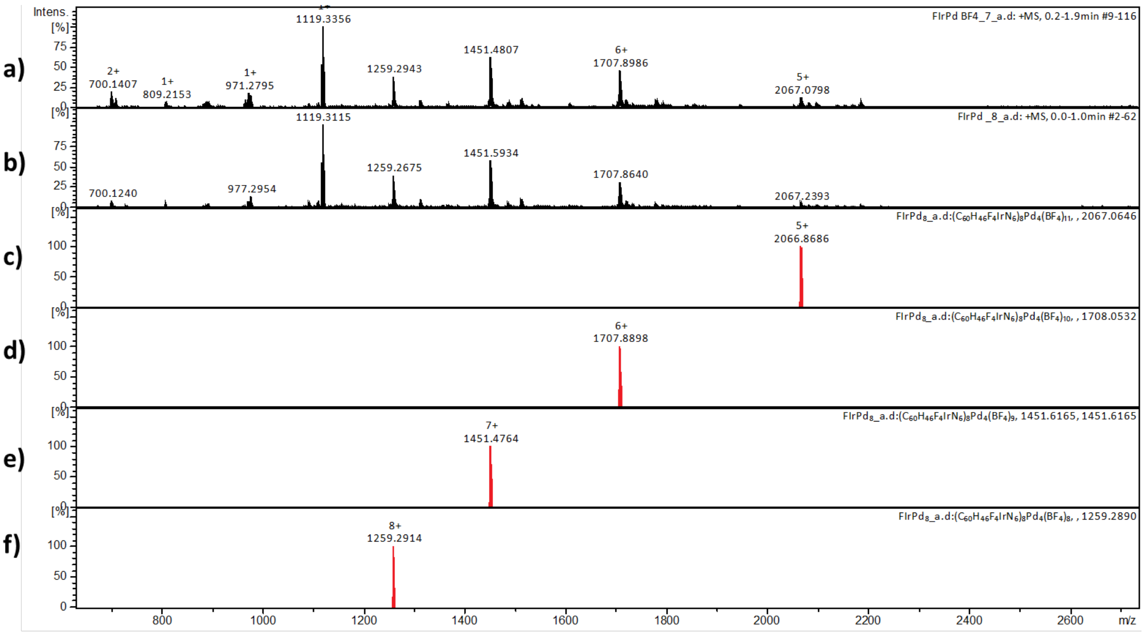


Figure **S38**. ESI-MS spectra of: **a)** $\Delta_8$**-C2-BF$_4$** and **b)** $\Lambda_8$**-C2-BF$_4$**. Simulated MS spectra of: **c)** [**C2**-$(BF_4)_5$]$^{5+}$; **d)** [**C2**-$(BF_4)_6$]$^{6+}$; **e)** [**C2**-$(BF_4)_7$]$^{7+}$ and **f)** [**C2**-$(BF_4)_8$]$^{8+}$.

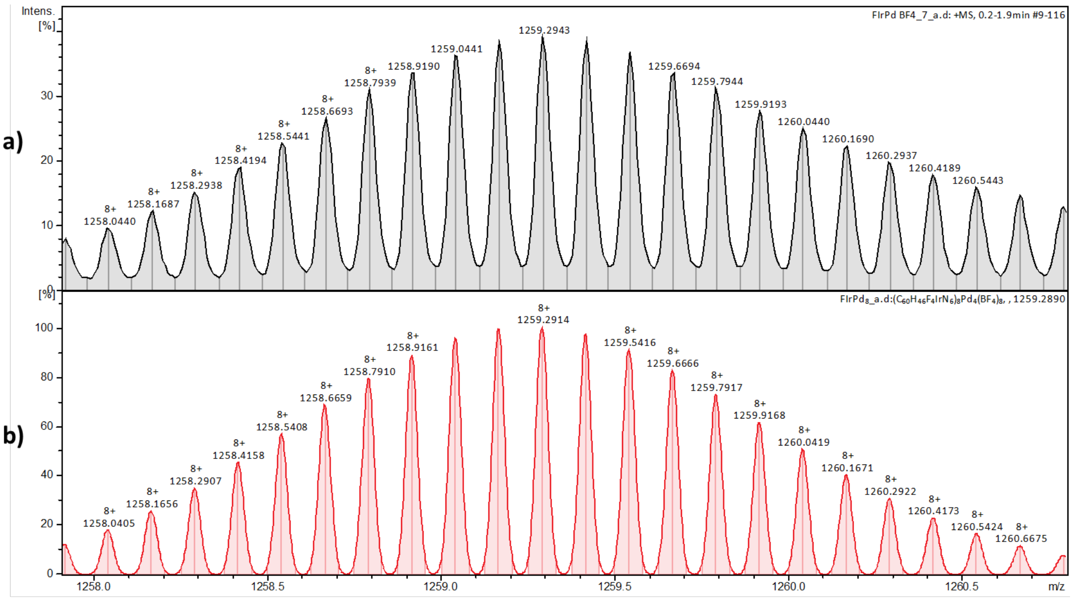


Figure **S39**. **a)** ESI-MS spectra of the [**C2**-$(BF_4)_8$]$^{8+}$ of $\Delta_8$**-C2-BF$_4$**; **b)** Simulation of isotopic distribution pattern of [**C2**-$(BF_4)_8$]$^{8+}$.

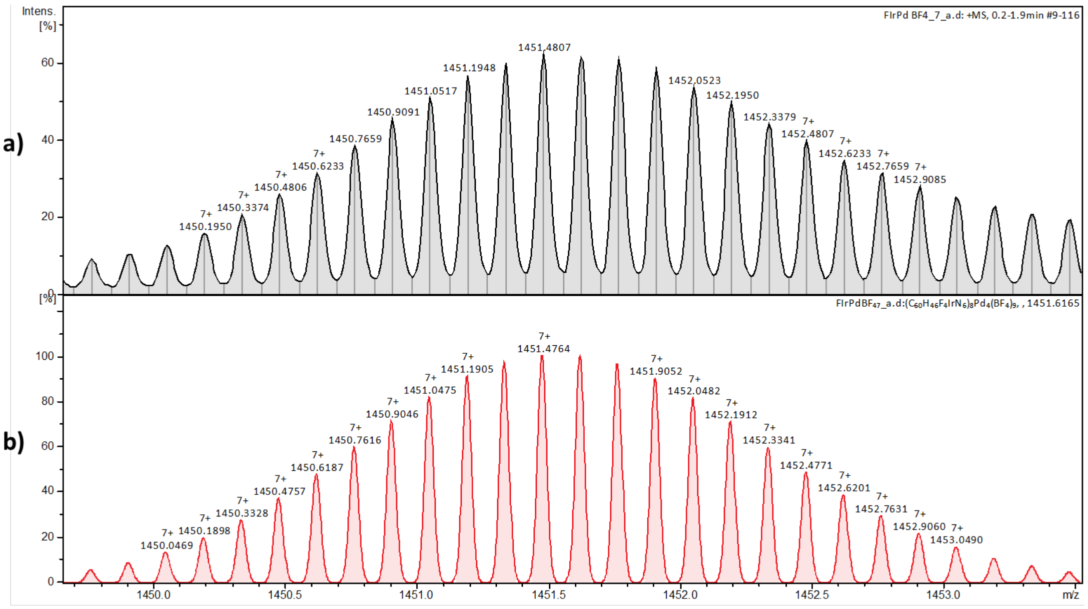


Figure **S40**. **a)** ESI-MS spectra of the [**C2**-$(BF_4)_7$]$^{7+}$ of **$\Delta_8$-C2-BF$_4$**; **b)** Simulation of isotopic distribution pattern of [**C2**-$(BF_4)_7$]$^{7+}$.

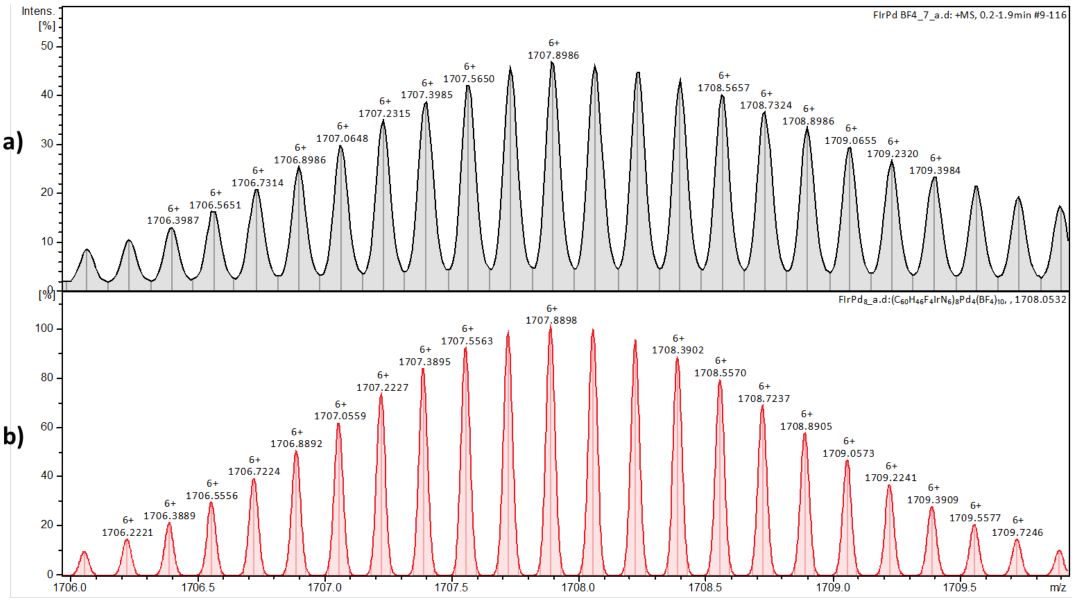


Figure **S41**. **a)** ESI-MS spectra of the [**C2**-$(BF_4)_6$]$^{6+}$ of **$\Delta_8$-C2-BF$_4$**; **b)** Simulation of isotopic distribution pattern of [**C2**-$(BF_4)_6$]$^{6+}$ with its simulation.

# Formation of Cages By Mixing Λ- or Δ-1 and Δ-2 with $Pd^{2+}$ ions.

Experiment design: In a dry 10 mL Schlenk vial, one of complexes **Λ**- or **Δ-1** (1 equiv.) as well as **Δ-2** (1 equiv.) and $[Pd(NCMe)_4](BF_4)_2$ (1 equiv.) were dissolved in DMSO-$d_6$ (1 mL) to give a concentration of the iridium metalloligands of approximately 0.05 M. The solution was degassed for five minutes by bubbling nitrogen and then heated at 85 °C for 12 h under nitrogen atmosphere. The solution was cooled to room temperature. The black solid was filtered through celite and the resulting DMSO solution was analysed directly by ESI-MS spectrometry. The formation of statistical mixtures of cages species comprising various degrees of the two metallo-ligands $[(\mathbf{\Lambda\text{-}/\Delta\text{-}1})_n(\mathbf{\Delta\text{-}2})_m Pd_4](BF_4)_{16}$ (n + m = 8, from **Λ-/Δ-1** : **Δ-2** = 7 : 1 to **Λ-/Δ-1** : **Δ-2** = 1 : 7) has been observed.

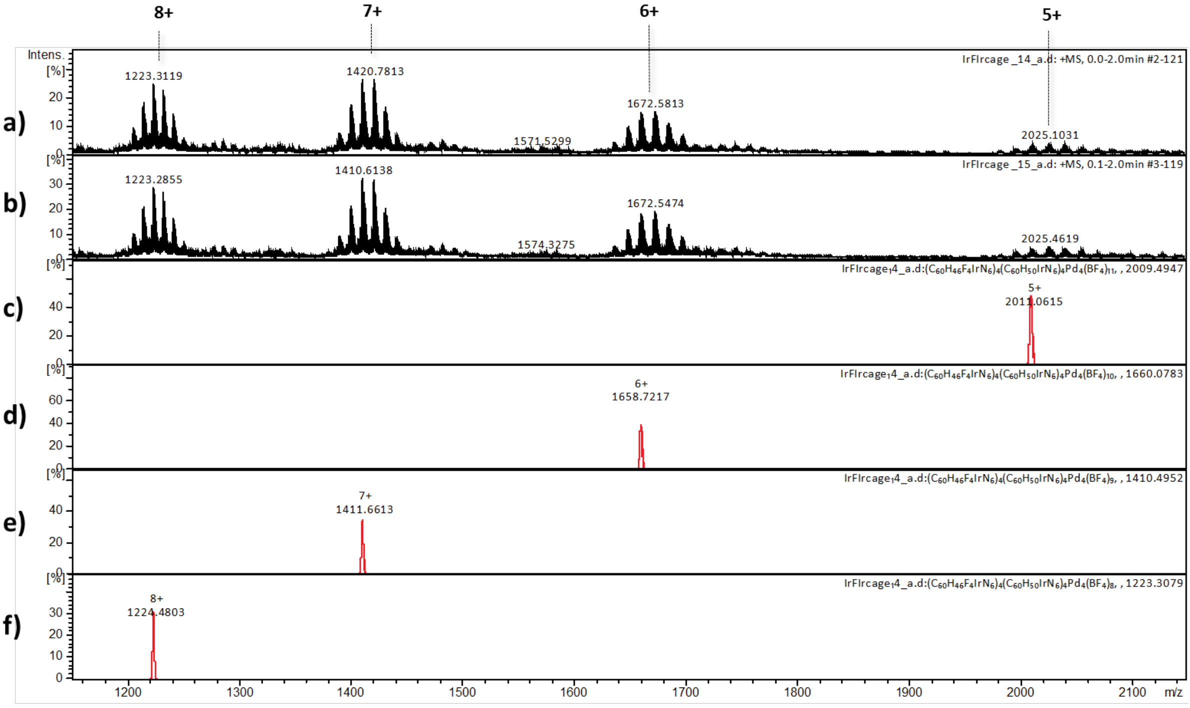


Figure **S42**. ESI-MS spectra of: **a) Δ-1-BF$_4$ + Δ-2-BF$_4$** + $[Pd(NCMe)_4](BF_4)_2$; **b) Λ-1-BF$_4$ + Δ-2-BF$_4$** + $[Pd(NCMe)_4](BF_4)_2$; Simulated MS spectra of: **c)** $[(\mathbf{1})_4(\mathbf{2})_4\text{-}(BF_4)_5]^{5+}$; **d)** $[(\mathbf{1})_4(\mathbf{2})_4\text{-}(BF_4)_6]^{6+}$; **e)** $[(\mathbf{1})_4(\mathbf{2})_4\text{-}(BF_4)_7]^{7+}$ and **f)** $[(\mathbf{1})_4(\mathbf{2})_4\text{-}(BF_4)_8]^{8+}$.

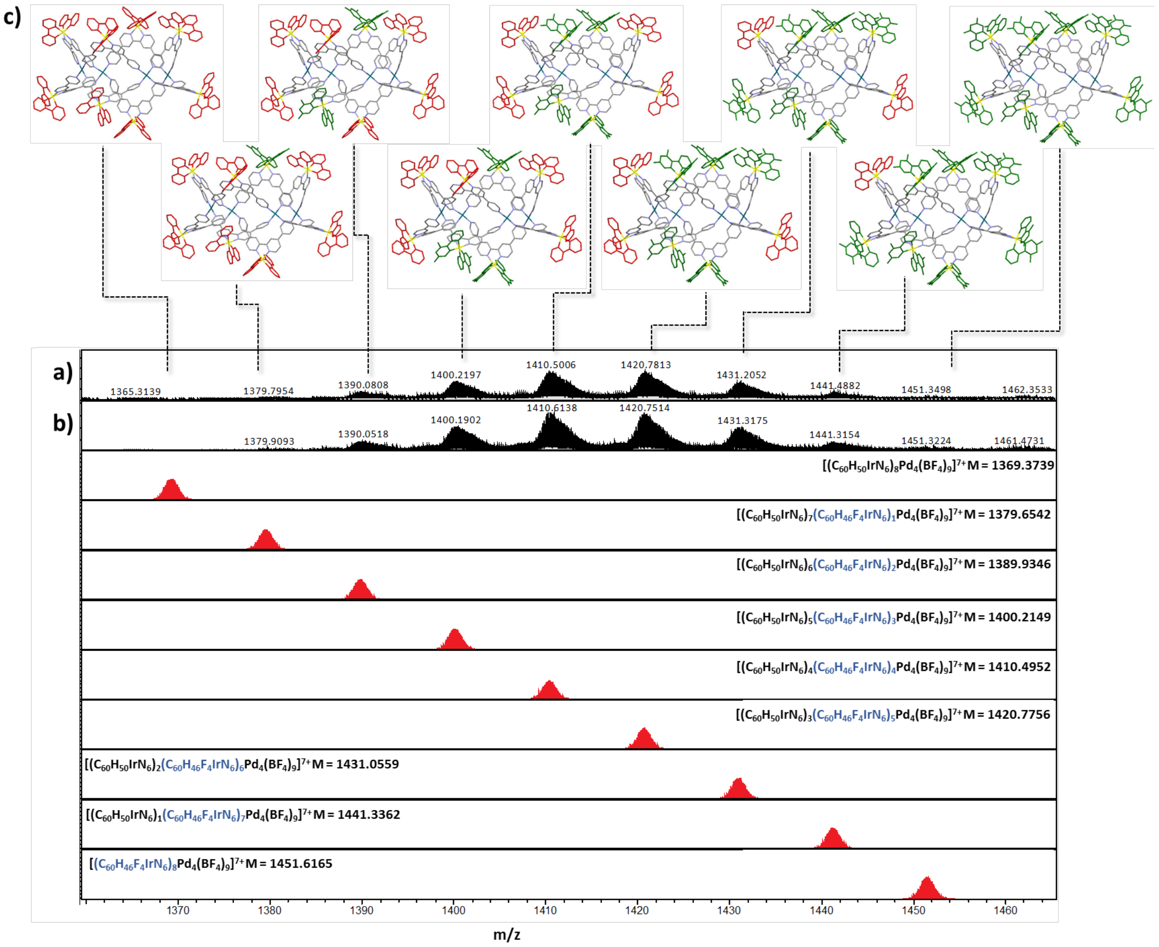


Figure **S43**. Illustration of the formation of statistical mixture of cages species of compositions [(**Λ-/Δ-1**)$_n$(**Δ-2**)$_m$Pd$_4$](BF$_4$)$_{16}$ (n + m = 8, from **Λ-/Δ-1** : **Δ-2** = 7 : 1 to **Λ-/Δ-1** : **Δ-2** = 1 : 7). ESI-MS spectra of the 7+ charge states [(**1**)$_n$(**2**)$_m$-(BF$_4$)$_7$]$^{7+}$ (n + m = 8, from **1** : **2** = 7 : 1 to **1** : **2** = 1 : 7) of: **a) Δ-1-BF$_4$** + **Δ-2-BF$_4$** + [Pd(NCMe)$_4$](BF$_4$)$_2$ and **b) Λ-1-BF$_4$** + **Δ-2-BF$_4$** + [Pd(NCMe)$_4$](BF$_4$)$_2$. In red are illustrated the simulated 7+ charge states of the heteroleptic cages. The same statistical distributions are also observed for the 8+, 6+ and 5+ charge states. **c)** representation of the heteroleptic cages; in red are illustrated the ppy ligands whereas in green the dFppy ligands. The mesityl substituents have been omitted for clarity.

# Formation of Heteronuclear Cages by Mixing the Homonuclear Cages $\Delta_8$- or $\Lambda_8$-C1 and $\Delta_8$-C2.

Experiment design: In a dry 10 mL Schlenk vial, cages **$\Lambda_8$**- or **$\Delta_8$-1** (1 equiv.) and **$\Delta_8$-2** (1 equiv.) were dissolved in DMSO-*d*$_6$ (1 mL) to give a concentration of the iridium cages of approximately 0.05 M. The solution was degassed for few minutes by bubbling nitrogen and **a)** heated at 85 °C for 12 h under nitrogen atmosphere or **b)** stirred at room temperature for 12h. The solution was cooled to room temperature and analysed directly by ESI-MS spectrometry. **a)** The formation of statistical mixtures of cages species comprising various degree of the two metalloligands [(**Λ-/Δ-1**)$_n$(**Δ-2**)$_m$Pd$_4$](BF$_4$)$_{16}$ (n + m = 8, from **Λ-/Δ-1** : **Δ-2** = 7 : 1 to **Λ-/Δ-1** : **Δ-2** = 1 : 7) have been observed when the cages were mixed at 85 °C. **b)** No ligand exchange was observed when the cages were mixed at room temperature.

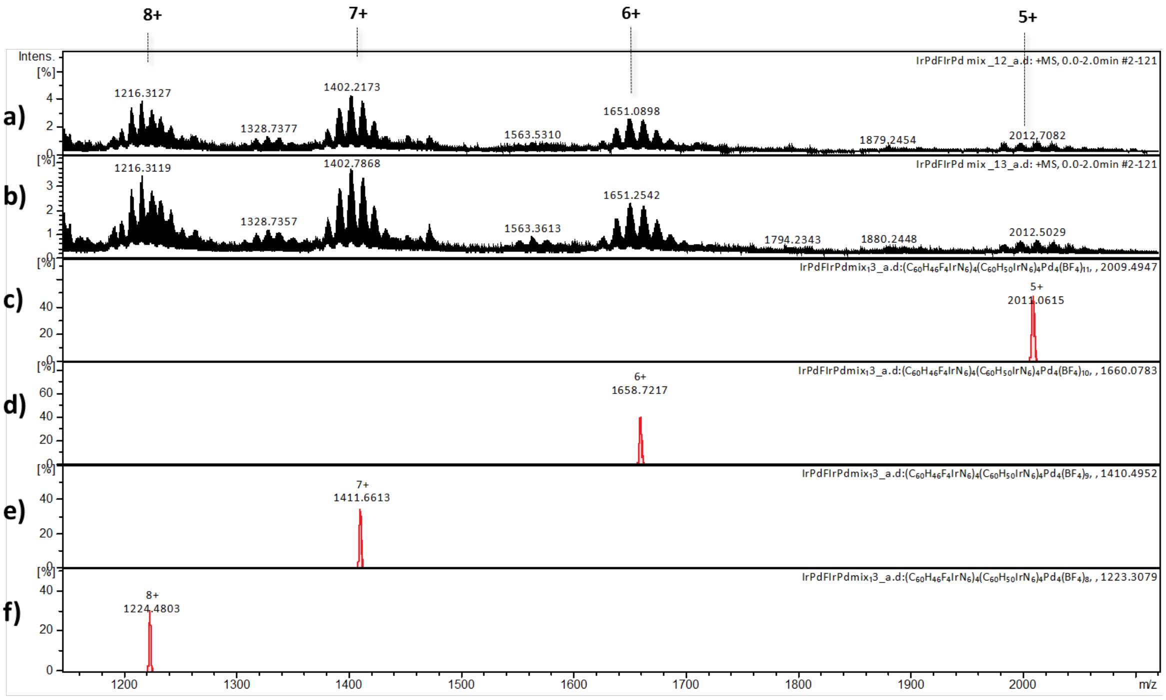


Figure **S44**. ESI-MS spectra of: **a) $\Delta_8$-C1-BF$_4$** + **$\Delta_8$-C2-BF$_4$** stirred at 85 °C for 12 h; **b) $\Lambda_8$-C1-BF$_4$** + **$\Delta_8$-C2-BF$_4$** stirred at 85 °C for 12 h; Simulated MS spectra of: **c)** [(**1**)$_4$(**2**)$_4$-(BF$_4$)$_5$]$^{5+}$; **d)** [(**1**)$_4$(**2**)$_4$-(BF$_4$)$_6$]$^{6+}$; **e)** [(**1**)$_4$(**2**)$_4$-(BF$_4$)$_7$]$^{7+}$ and **f)** [(**1**)$_4$(**2**)$_4$-(BF$_4$)$_8$]$^{8+}$.

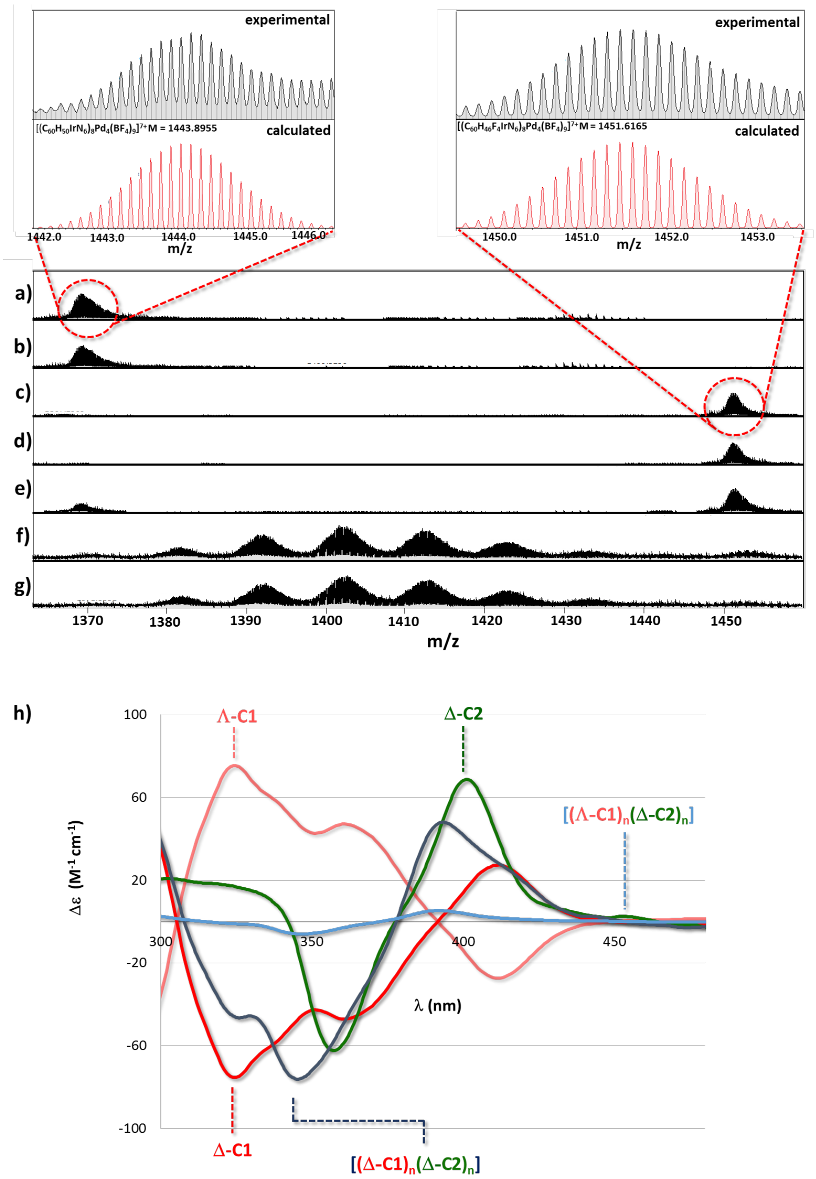


Figure **S45**. **a)** ESI mass spectrum of **Δ-C1** indicating the $\Delta_8$-[$\mathbf{1}_8$-$(BF_4)_7$]$^{7+}$ charge state with its simulation; **b)** ESI mass spectrum of **Λ-C1** indicating the $\Lambda_8$-[$\mathbf{1}_8$-$(BF_4)_7$]$^{7+}$ charge state; **c)** ESI mass spectrum of **Δ-C2** indicating the $\Delta_8$-[$\mathbf{2}_8$-$(BF_4)_7$]$^{7+}$ charge state with its simulation; **d)** ESI mass spectrum of **Λ-C2** indicating the $\Lambda_8$-[$\mathbf{2}_8$-$(BF_4)_7$]$^{7+}$ charge state; **e)** ESI mass spectrum of a mixture of **Δ-C1** and **Δ-C2** mixed at 273 K; **f)** ESI mass spectrum of a mixture of **Δ-C1** and **Δ-C2** mixed at 358 K for 16h; **g)** ESI mass spectrum of a mixture of **Λ-C1** and **Δ-C2** mixed at 358 K for 16h; **h)** CD spectra of **Δ-C1** (red line), **Λ-C1** (light-red line), **Δ-C2** (green line) and mixed cages of compositions [(**Δ-C1**)$_n$(**Δ-C2**)$_n$]$(BF_4)_{16}$ (dark-blue line) and [(**Λ-C1**)$_n$(**Δ-C2**)$_n$]$(BF_4)_{16}$ (light-blue line).

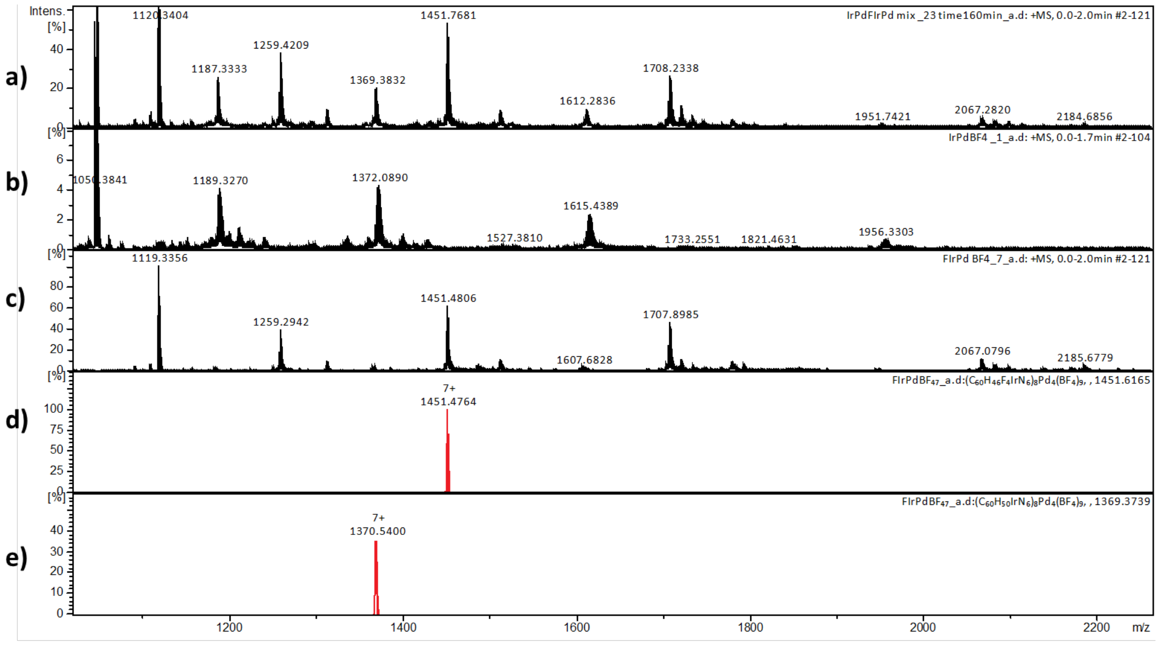


Figure **S46**. ESI-MS spectra of: **a) $\Delta_8$-C1-BF$_4$** + **$\Delta_8$-C2-BF$_4$** stirred at RT °C for 12 h; **b) $\Delta_8$-C1-BF$_4$**; **c) $\Delta_8$-C2-BF$_4$**. **d)** simulated MS spectra of $[(\mathbf{2})_8\text{-}(BF_4)_7]^{7+}$; **e)** simulated MS spectra of $[(\mathbf{2})_8\text{-}(BF_4)_7]^{7+}$.

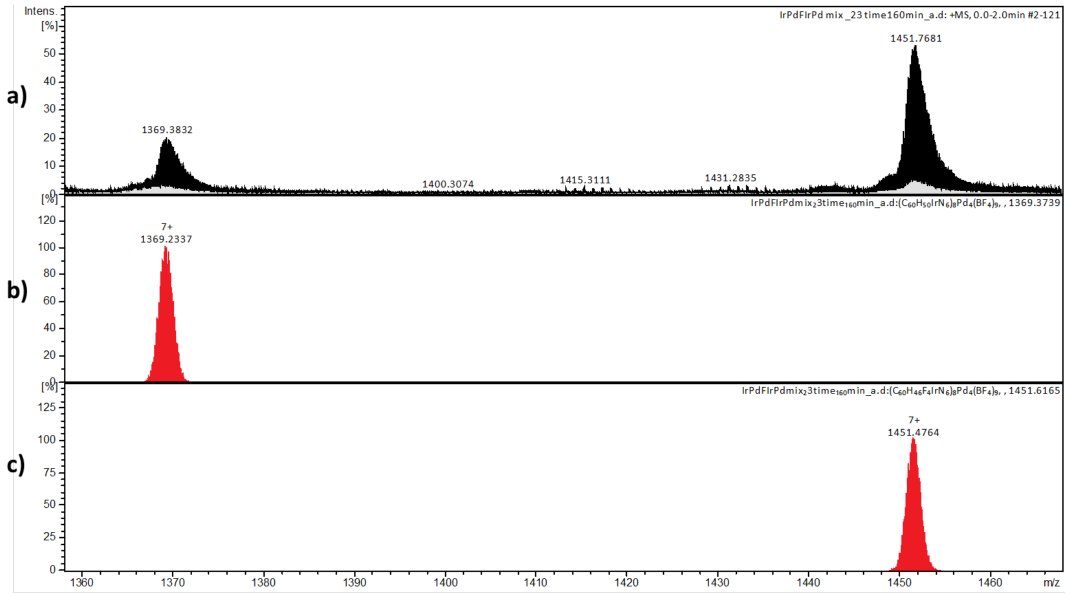


**Figure S47. a)** ESI-MS spectra illustrating the 7+ charge states $\Delta_8$-[**1**$_8$-(BF$_4$)$_7$]$^{7+}$ and $\Delta_8$-[**2**$_8$-(BF$_4$)$_7$]$^{7+}$ of a mixture of $\Delta_8$-**C1** and $\Delta_8$-**C2** left at RT for 12h. **b)** simulated MS spectra of $\Delta_8$-[**1**$_8$-(BF$_4$)$_7$]$^{7+}$ and **c)** simulated MS spectra of $\Delta_8$-[**2**$_8$-(BF$_4$)$_7$]$^{7+}$.

# Host-Guest Assemblies Monitored by NMR Spectroscopies.

Host-Guest chemistry with small organic compounds.

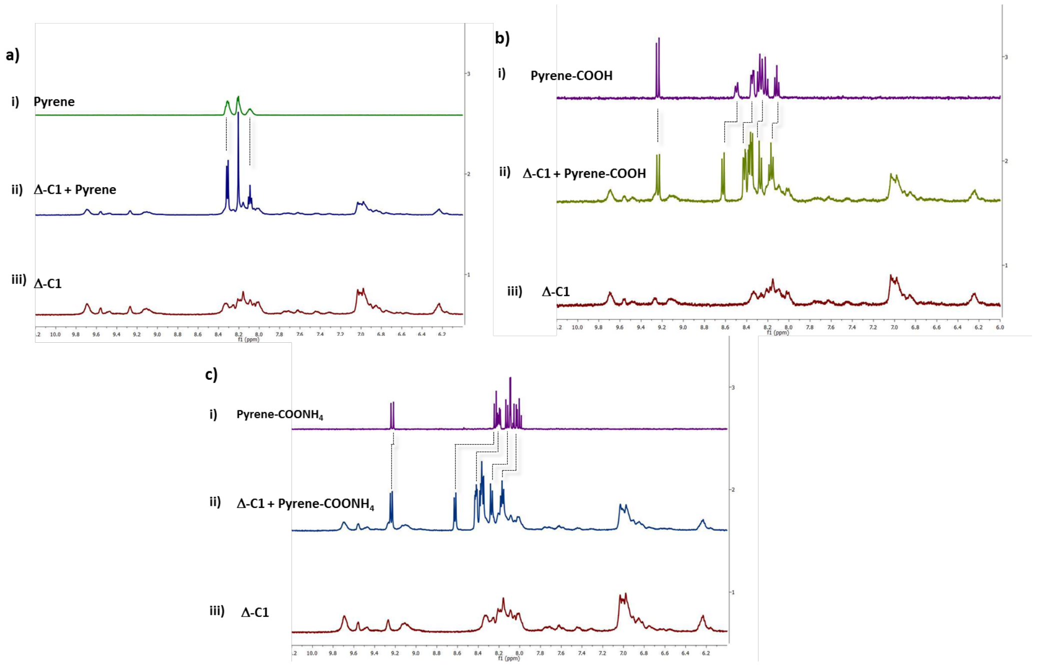


Figure **S48**. $^{1}$H NMR spectra collected in DMSO-$d_6$ at 298 K of: **a) i)** pyrene; **ii)** pyrene + **Δ-C1**; **iii) Δ-C1**. **b) i)** pyrene-carboxylic acid; **ii)** pyrene carboxylic acid + **Δ-C1**; **iii) Δ-C1**. **c) i)** ammonium pyrene carboxylate; **ii)** ammonium pyrene carboxylate + **Δ-C1**; **iii) Δ-C1**. The concentration of **Δ-C1** was kept constant at 3.0 mM and one equivalent of each guest was added.

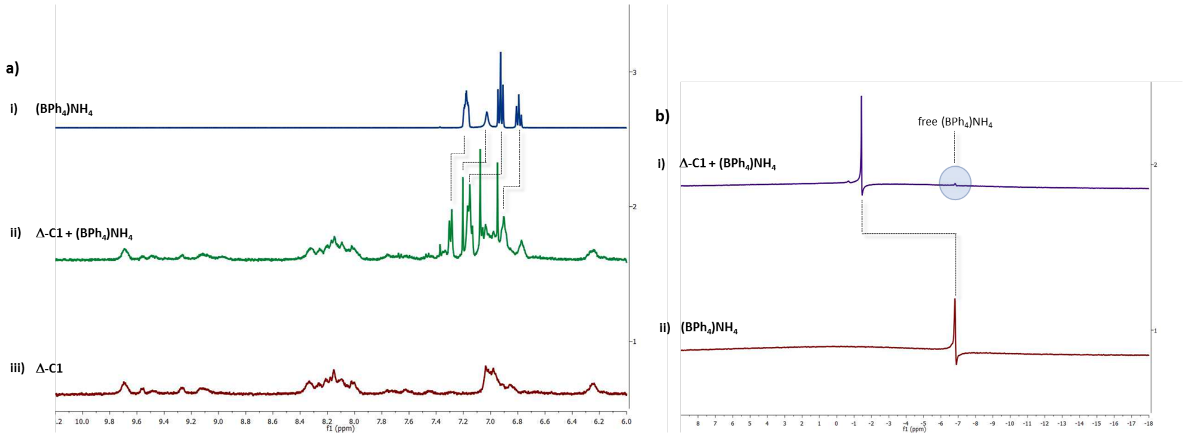


Figure **S49**. $^{1}$H NMR spectra collected in DMSO-$d_6$ at 298 K of: **a) i)** ammonium tetraphenylborate; **ii)** ammonium tetraphenylborate + **Δ-C1**; **iii) Δ-C1**. **b)** $^{11}$B NMR collected in

DMSO-$d_6$ at 298 K of: **i)** ammonium tetraphenylborate + **Δ-C1**; **ii)** ammonium tetraphenylborate. The concentration of **Δ-C1** was kept constant at 3.0 mM and one equivalent of each guest was added.

## Host-Guest chemistry with TBA[Ir(dFppy)$_2$(CN)$_2$], IrCN.

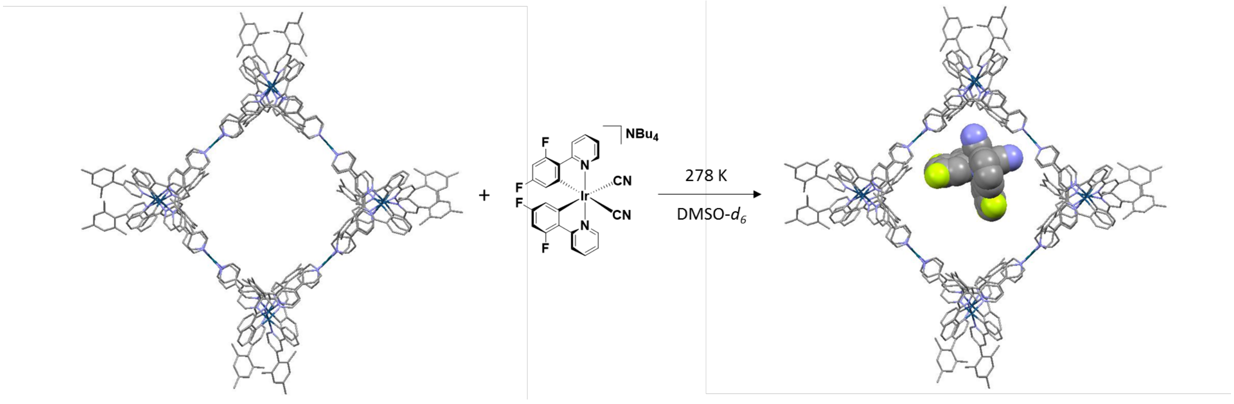


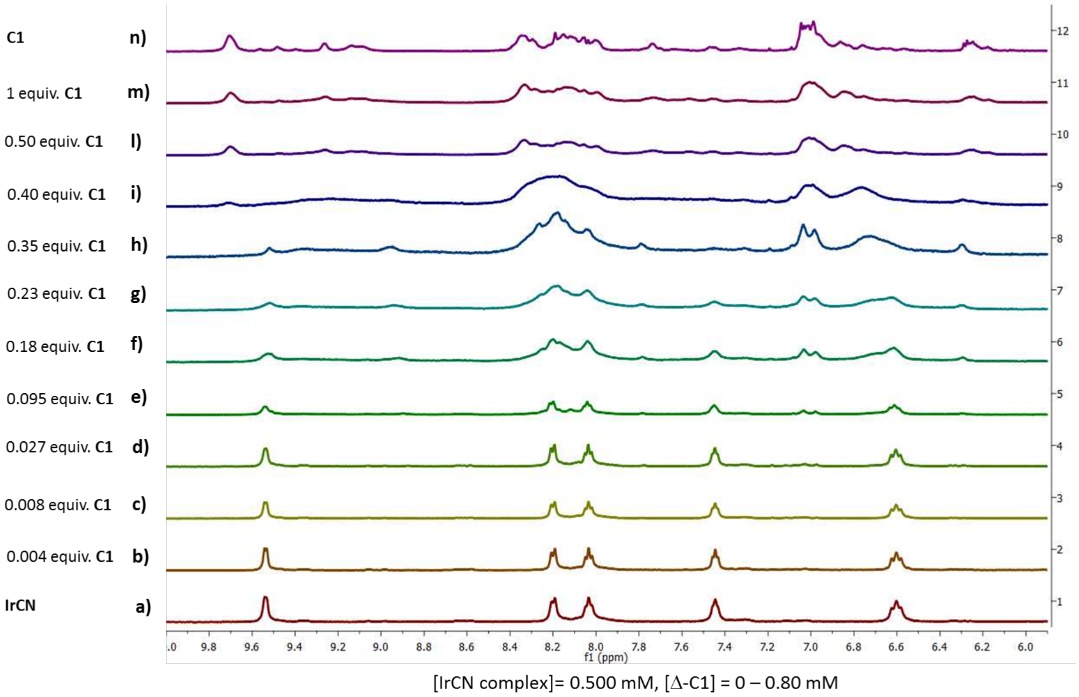


Figure **S50**. $^1$H NMR titration spectra collected in DMSO-$d_6$ at 298 K of: **a) IrCN** (the concentration of IrCN was kept constant at 0.5 mM during the titration experiment); **b)** to **m) IrCN + ** gradual addition of **Δ-C1** (from 0.10 mM to 0.8 mM); **c) Δ-C1**.

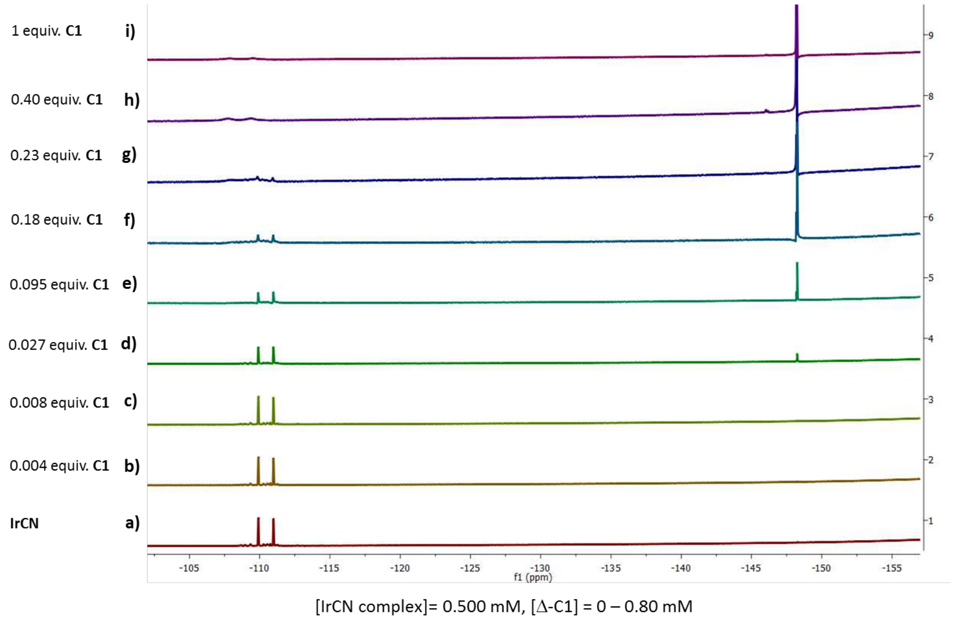


Figure **S51**. $^{19}$F NMR titration spectra collected in DMSO-$d_6$ at 298 K of: **a) IrCN** (the concentration of IrCN was kept constant at 0.5 mM during the titration experiment); **b)** to **m) IrCN + ** gradual addition of **Δ-C1** (from 0.10 mM to 0.8 mM); **c) Δ-C1**.

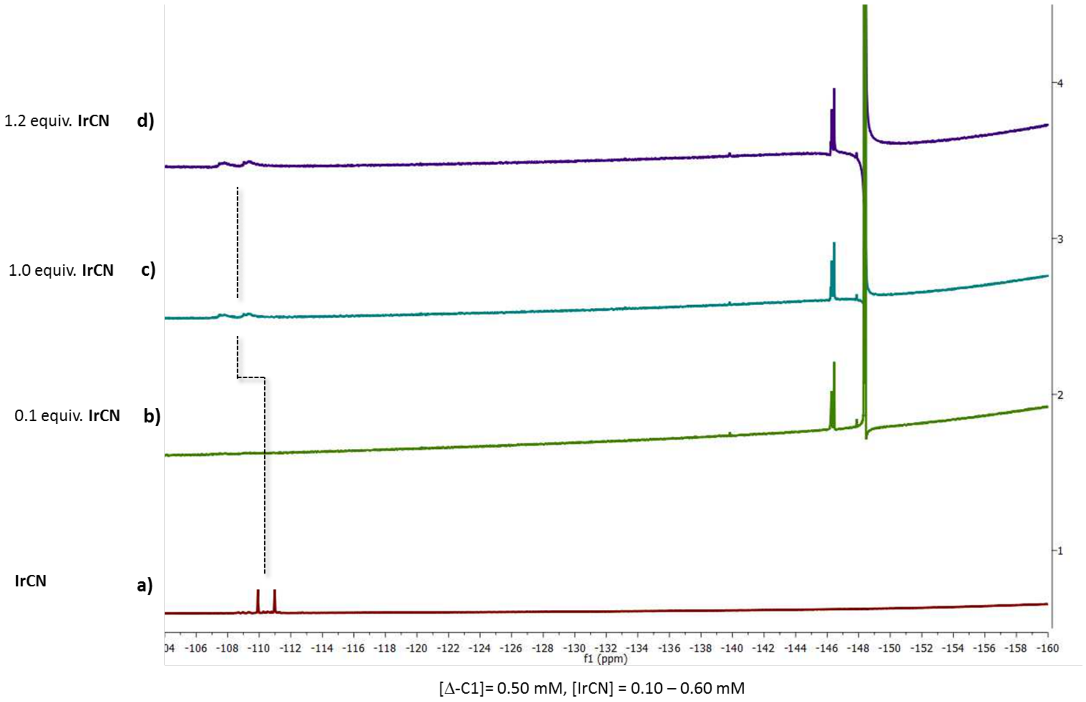


Figure **S52**. $^{19}$F NMR titration spectra collected in DMSO-$d_6$ at 298 K of: **a) IrCN**; **b)** to **d) Δ-1** cage **+** gradual addition of **IrCN** (from 0.10 mM to 0.6 mM). The concentration of **Δ-1** cage was kept constant at 0.50 mM.

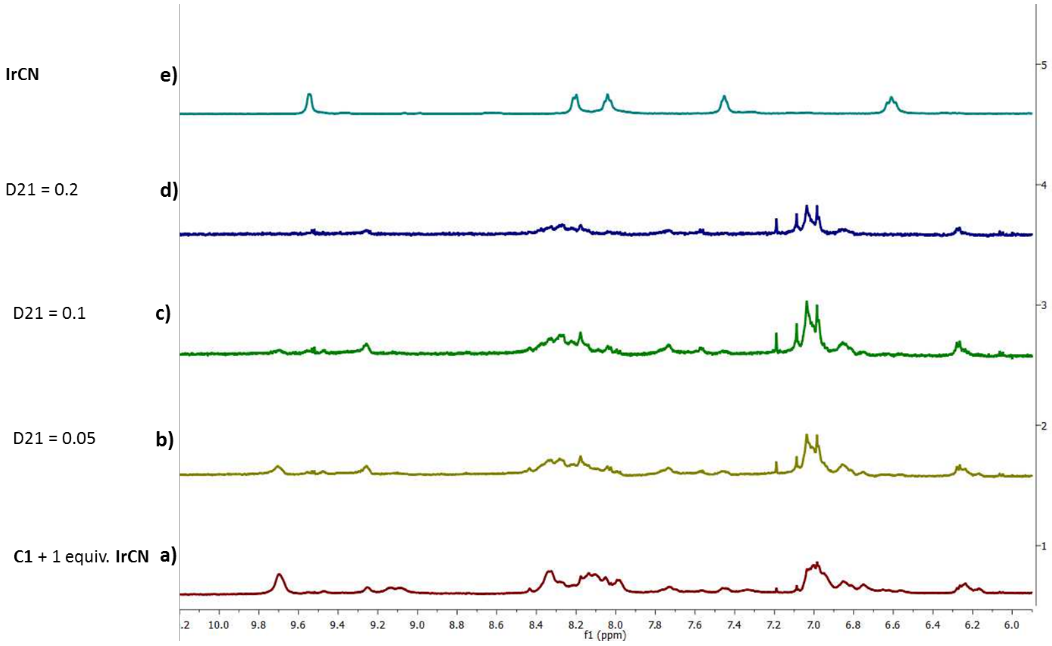


Figure **S53**. $^1$H NMR CPMGZ spectra collected in DMSO-$d_6$ at 298 K of **Δ-C1 +** 1 equiv. of **IrCN**. Filter conditions: **a)** no filter applied; **b)** D21 = 0.05; c) D21 = 0.1; d) D21 = 0.2. **e)** $^1$H NMR of **IrCN** with no filter applied.

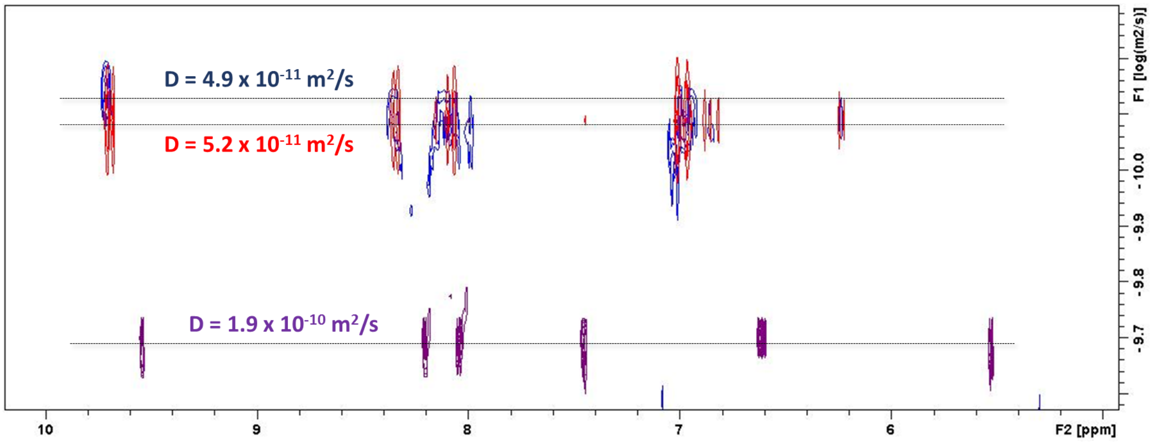


Figure **S54**. $^1$H DOSY NMR spectra collected in DMSO-$d_6$ at 298 K of: **IrCN** (in purple, D = 1.9 × $10^{-10}$ $m^2$/s); **Δ-C1** (in red, D = 4.9 × $10^{-10}$ $m^2$/s) and a 1:1 mixture of **IrCN** and **Δ-C1** (in blue, D = 4.9 × $10^{-10}$ $m^2$/s).

**Host-Guest chemistry with $[Ir(dFppy)_2(dmbpy)]PF_6$, Irdmbpy.**

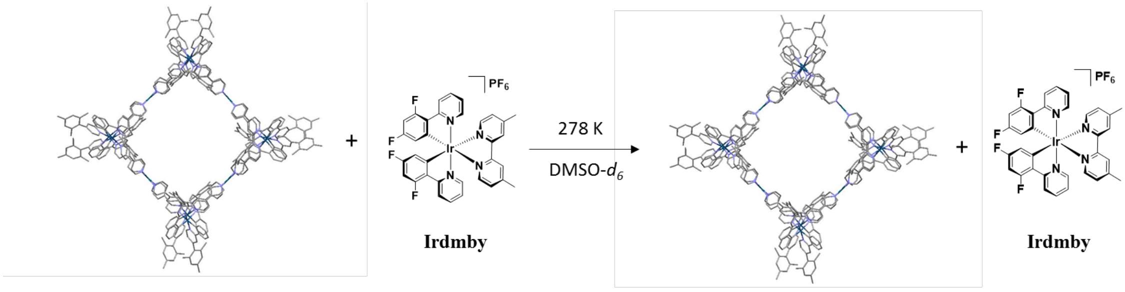


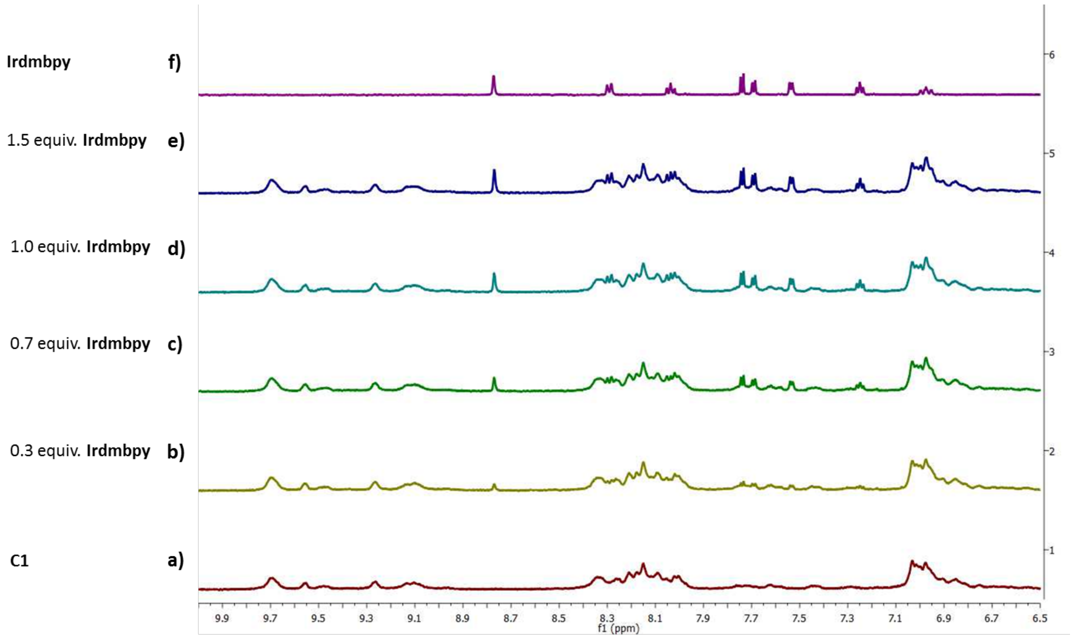


[Δ-C1]= 0.50 mM, [Irdmbpy] = 0 – 0.80 mM

Figure **S55**. $^1$H NMR titration spectra collected in DMSO-$d_6$ at 298 K of: **a) Δ-C1** (the concentration of **Δ-C1** was kept constant at 0.5 mM during the titration experiment); **b)** to **e) Δ-C1 +** gradual addition of **Irdmbpy** (from 0.10 mM to 0.80 mM); **f) Irdmbpy**.

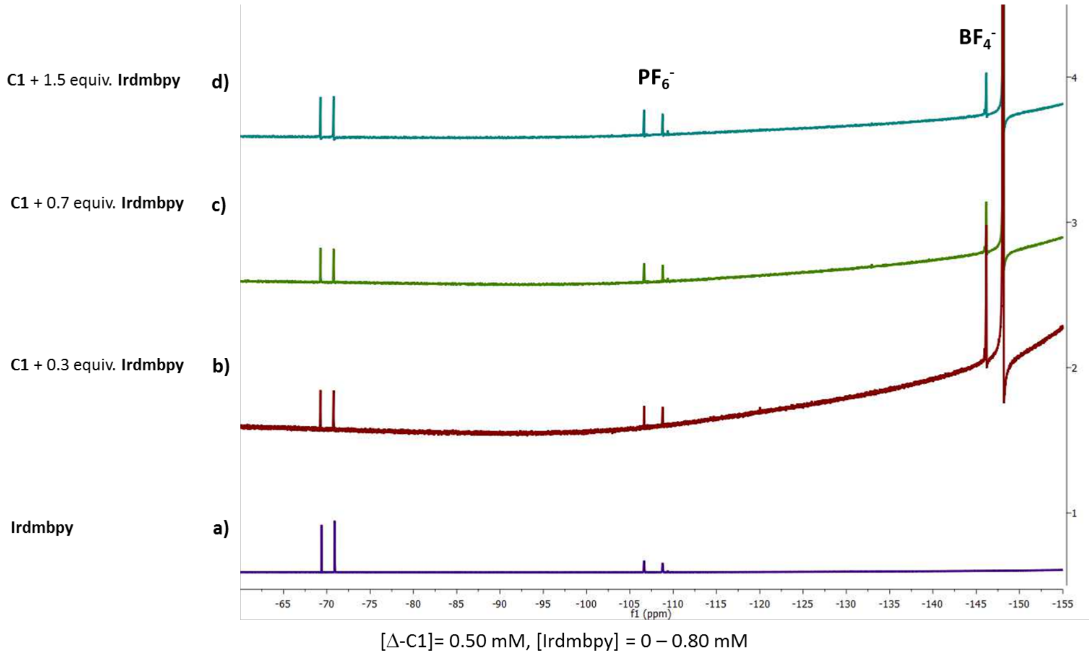


Figure **S56**. $^{19}$F NMR titration spectra collected in DMSO-$d_6$ at 298 K of: **a) Irdmbpy**; **b)** to **d) Δ-C1 +** gradual addition of **Irdmbpy** (from 0.10 mM to 0.80 mM). The concentration of **Δ-C1** was kept constant at 0.5 mM.

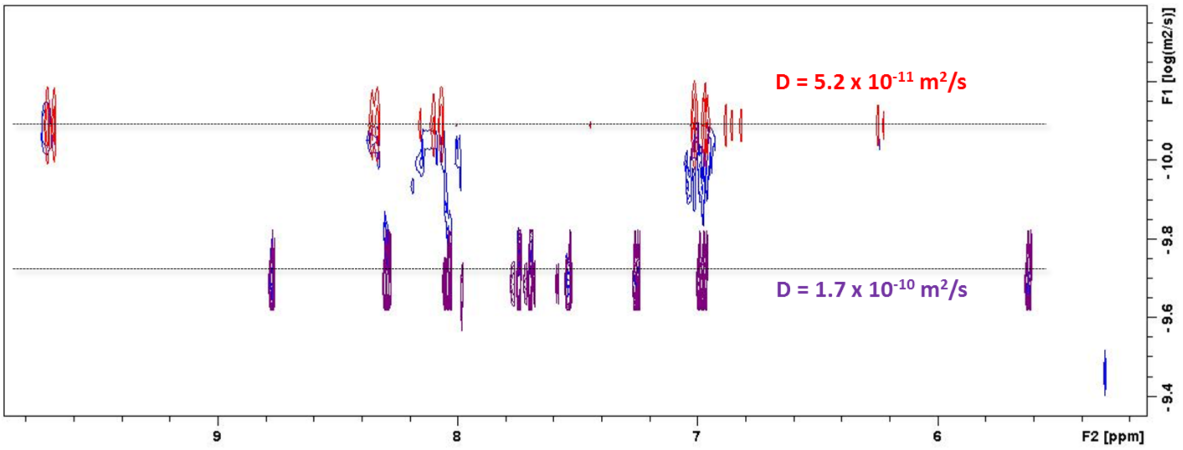


Figure **S57**. $^{1}$H DOSY NMR spectra collected in DMSO-$d_6$ at 298 K of: **Irdmbpy** (in purple, D = $1.7 \times 10^{-10}$ m$^2$/s); **Δ-C1** (in red, D = $5.2 \times 10^{-11}$ m$^2$/s) and a 1:1 mixture of **Irdmbpy** and **Δ-C1** (in blue, D = $5.2 \times 10^{-11}$ m$^2$/s - **Δ-C1** - and $1.7 \times 10^{-10}$ m$^2$/s - **Irdmbpy**).

## Control experiment by mixing [Ir(mesppy)$_2$(qpy)$_2$]BF$_4$, Δ-1 with TBA[Ir(dFppy)$_2$(CN)$_2$], Irdmbpy.

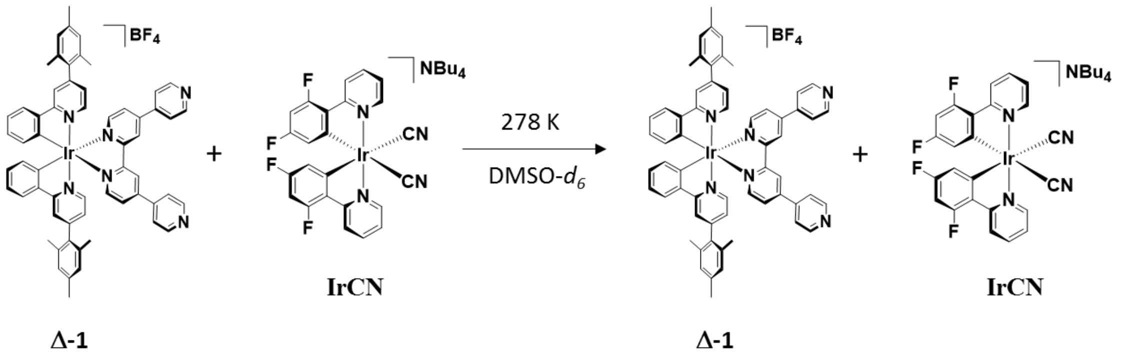


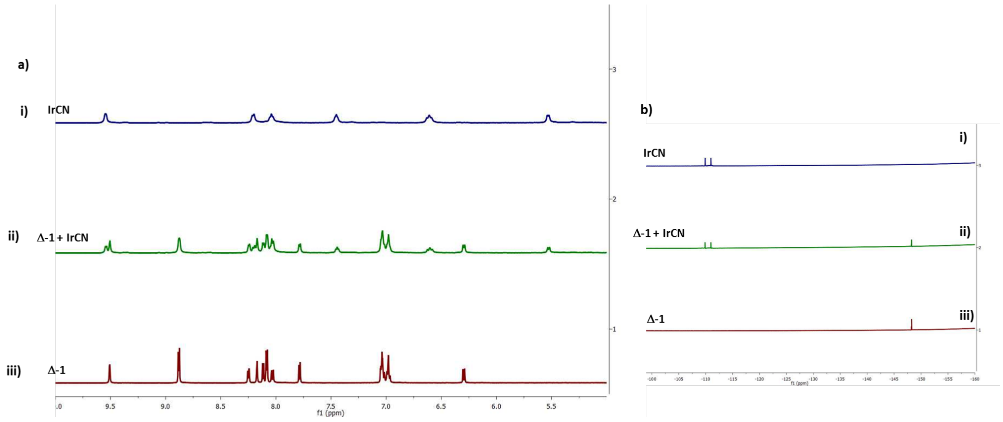


Figure **S58**. $^{1}$H NMR and 19 F NMR spectra, respectively **a)** and **b)**, collected in DMSO-$d_6$ at 298 K of: **i) IrCN**; **ii) Δ-1** (1 equiv.) **+ IrCN** (1 equiv.); **iii) Δ-1**.

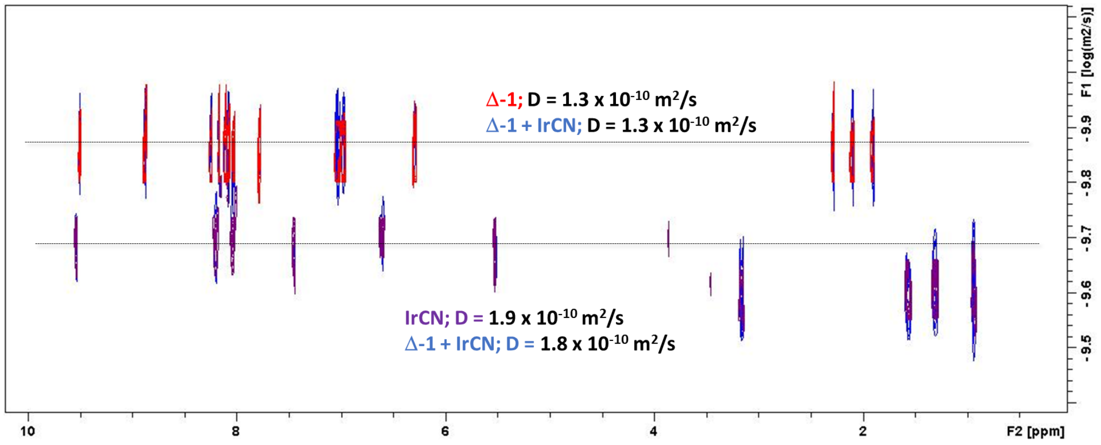


Figure **S59**. $^{1}$H DOSY NMR spectra collected in DMSO-$d_6$ at 298 K of: **IrCN** (in purple, D = 1.9 × 10$^{-10}$ m$^2$/s); **Δ-1** (in red, D = 1.3 × 10$^{-10}$ m$^2$/s) and a 1:1 mixture of **IrCN** and **Δ-1** (in blue, D = 1.3 × 10$^{-10}$ m$^2$/s - **Δ-1** - and 1.8 × 10$^{-10}$ m$^2$/s - **IrCN**).

# Crystal Structures of Λ,Λ- and Δ,Δ-D2 and *rac*-2

X-ray diffraction data for compounds **Λ,Λ-** and **Δ,Δ-D2** and ***rac*-2** were collected at 93 K by using a Rigaku FR-X Ultrahigh brilliance Microfocus RA generator/confocal optics and Rigaku XtaLAB P200 system, with Mo Kα radiation (λ = 0.71075 Å). Intensity data were collected using ω steps accumulating area detector images spanning at least a hemisphere of reciprocal space. All data were corrected for Lorentz polarization effects. A multiscan absorption correction was applied by using either CrystalClear (**Λ,Λ-D2** and ***rac*-2**)[7] or CrysalisPro (**Δ,Δ-D2**).[8] Structures were solved by Patterson methods (PATTY)[9] and refined by full-matrix least-squares against $F^2$ (SHELXL-2014).[10] Non-hydrogen atoms were refined anisotropically, and hydrogen atoms were refined using a riding model. All calculations were performed using the CrystalStructure[11] interface. CCDC 1543500-1543502 contains the supplementary crystallographic data for this paper. These data can be obtained free of charge via https://www.ccdc.cam.ac.uk/structures/ (or from the Cambridge Crystallographic Data Centre, 12, Union Road, Cambridge CB2 1EZ, UK; fax: +44 1223 336033). Crystallographic data are presented in Table **S2**. Thermal ellipsoid plots of the structures are presented in Figures **S59** (**Λ,Λ-** and **Δ,Δ-D2**) and **S60** (***rac*-2**).

Repeated attempts were made at collecting X-ray diffraction data on cages **C1** and **C2**. The best data obtained to date, for *rac*-**C1**, was collected at beamline I19, Diamond Light Source, Didcot, UK using silicon double crystal monochromated radiation (λ = 0.6889 Å, Pilatus 2M detector),[12] at 100 K. A multiscan absorption correction was applied by using CrysalisPro.[8] A solution was obtained using charge-flipping methods (Superflip),[13] which revealed the positions of the metal centers and an approximation of the structure of the ligand (Figure S62), however it has not, as yet, been possible to refine the data. Calculations were performed using the WinGX interface.[14]

**Table S2**. Crystallographic data for complexes **Λ,Λ-** and **Δ,Δ-D2** and ***rac*-2**.

| | **Λ,Λ-D2** | **Δ,Δ-D2** | ***rac*-2** |
|---|---|---|---|
| CCDC number | 1543500 | 1543501 | 1543502 |
| Formula | $C_{81}H_{66}Cl_4F_8Ir_2N_4$ | $C_{81}H_{66}Cl_4F_8Ir_2N_4$ | $C_{64}H_{56}BF_8IrN_6O$ |
| Formula weight | 1773.68 | 1773.68 | 1280.20 |
| Crystal colour, habit | Yellow, rod | Yellow, rod | Yellow, prism |
| Crystal dimensions ($mm^3$) | 0.13×0.03×0.02 | 0.13×0.02×0.02 | 0.13×0.09×0.07 |
| Space group | $P2_12_12_1$ | $P2_12_12_1$ | $P\bar{1}$ |
| a (Å) | 12.5492(16) | 12.496(4) | 13.320(2) |
| b (Å) | 24.138(3) | 24.105(7) | 14.856(2) |
| c (Å) | 26.122(3) | 26.041(8) | 19.963(4) |
| α (°) | | | 70.564(17) |
| β (°) | | | 81.23(2) |
| γ (°) | | | 67.778(18) |
| Volume ($Å^3$) | 7912.7(17) | 7844(4) | 3446.9(11) |
| Z | 4 | 4 | 2 |
| $\rho_{calcd}$ (g $cm^{-3}$) | 1.489 | 1.502 | 1.233 |
| Temperature (K) | 93 | 93 | 93 |
| μ ($mm^{-1}$) | 3.567 | 3.598 | 2.004 |
| $2\theta$ range | 2.298–25.242 | 1.836–30.140 | 1.947–25.360 |
| Reflections collected | 76850 | 123445 | 86700 |
| Independent reflections ($R_{int}$) | 14268 (0.0658) | 20443 (0.1817) | 12452 (0.1317) |
| $R_1$ [$I$>2σ($I$)] | 0.0488 | 0.0802 | 0.1078 |
| $wR_2$ (all data) | 0.1368 | 0.2194 | 0.3348 |
| Goodness of fit | 1.112 | 1.033 | 1.136 |
| Data/restraints/parameters | 14268/57/904 | 20443/921/904 | 12452/44/734 |
| Residual electron peak, hole | 2.82, -0.82 | 1.74, -3.99 | 4.45, -1.36 |

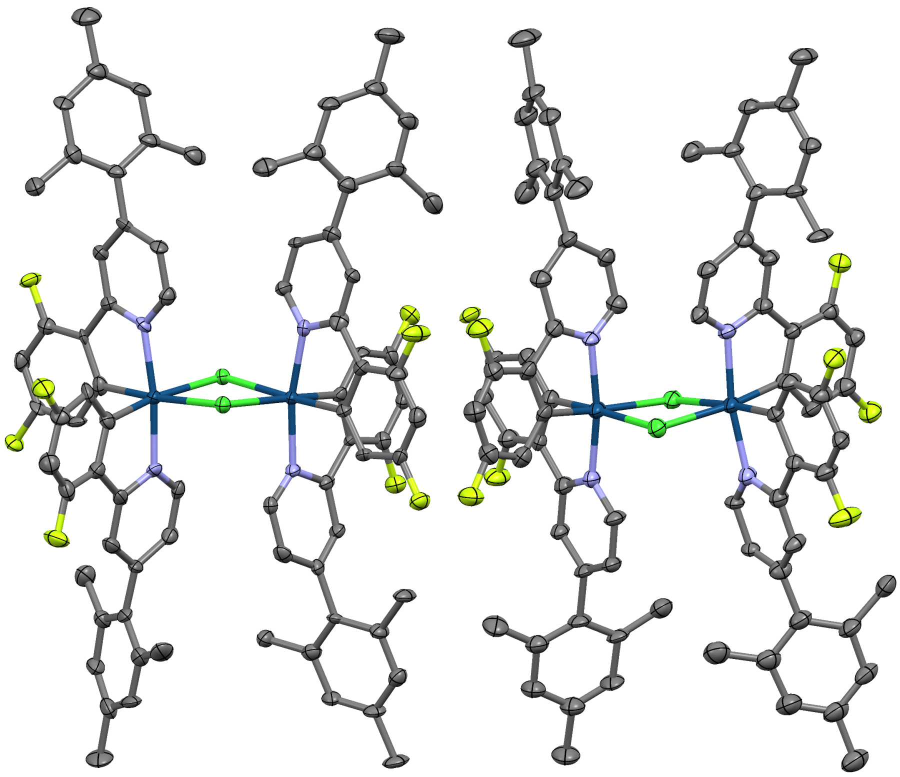

Figure **S60**. X-ray crystal structures of **a) Λ,Λ-[Ir(dFmesppy)$_2$(*μ*-Cl)]$_2$** (**Λ,Λ-D2**) and **b) Δ,Δ-[Ir(dFmesppy)$_2$(*μ*-Cl)]$_2$** (**Δ,Δ-D2**). Ellipsoids are drawn at the 50 % probability level. Solvent molecules and hydrogen atoms are omitted for clarity.

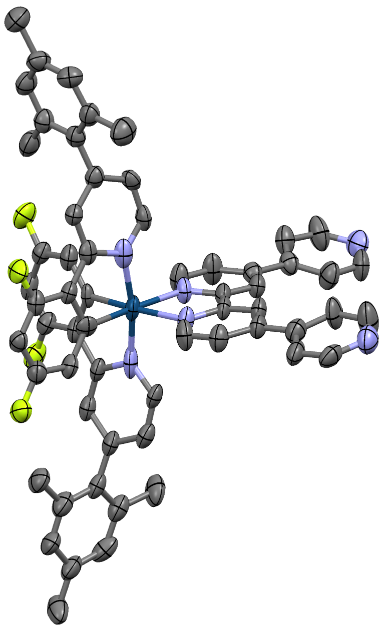

Figure **S61**. X-ray crystal structure of ***rac*-[Ir(dFmesppy)$_2$(qpy)]BF$_4$** (***rac*-2**). Ellipsoids are drawn at the 50 % probability level. Solvent molecules and hydrogen atoms are omitted for clarity.

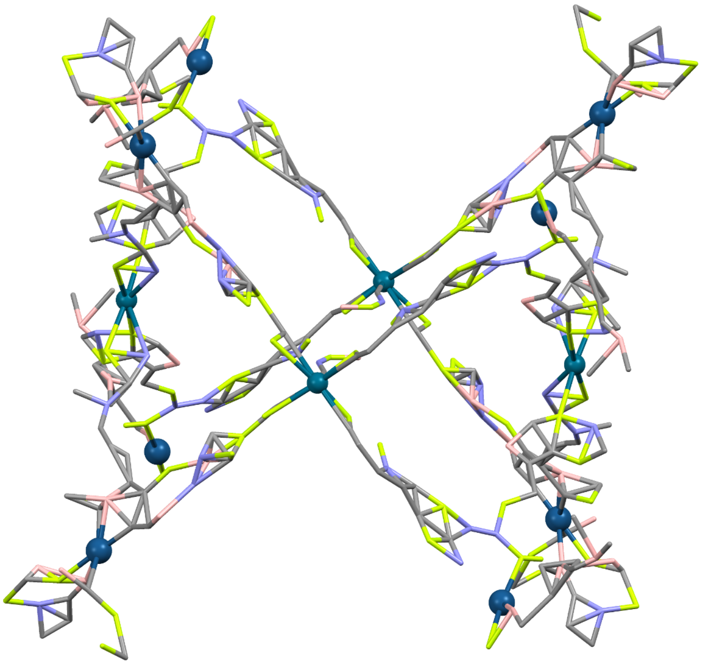

Figure **S62**. Preliminary X-ray crystal structure of the cage *rac*-**C1**. The data, as yet, isn't being amenable to further refinement due to the extremely weak diffraction exhibited by the single crystals.

# Optoelectronic Characterization.

*Photophysical measurements.* All samples were prepared in HPLC grade $CH_2Cl_2$ or DMSO with varying concentrations on the order of $10^{-4}$ – $10^{-6}$ M. Absorption spectra were recorded at room temperature using a Shimadzu UV-1800 double beam spectrophotometer. Molar absorptivity determination was verified by linear least-squares fit of the values obtained from at least four independent solutions at varying concentrations with absorbance ranging from $6.05 \times 10^{-5}$ to $2.07 \times 10^{-5}$ M.

The sample solutions for the emission spectra were prepared in HPLC-grade DCM or DMSO and degassed *via* freeze-pump-thaw cycles using a quartz cuvette designed in-house. Steady-state emission and excitation spectra and time-resolved emission spectra were recorded at 298 K using an Edinburgh Instruments F980. All samples for steady-state measurements were excited at 360 nm using a xenon lamp, while samples for time-resolved measurements were excited at 378 nm using a PDL 800-D pulsed diode laser. Photoluminescence quantum yields were determined using the optically dilute method.[15] A stock solution with absorbance of *ca.* 0.5 was prepared and then four dilutions were prepared with dilution factors between 2 and 20 to obtain solutions with absorbances of *ca.* 0.095 0.065, 0.05 and 0.018, respectively. The Beer-Lambert law was found to be linear at the concentrations of these solutions. The emission spectra were then measured after the solutions were rigorously degassed *via* three freeze-pump-thaw cycles prior to spectrum acquisition. For each sample, linearity between absorption and emission intensity was verified through linear regression analysis and additional measurements were acquired until the Pearson regression factor ($R^2$) for the linear fit of the data set surpassed 0.9. Individual relative quantum yield values were calculated for each solution and the values reported represent the slope value. The equation $\Phi_s = \Phi_r(A_r/A_s)(I_s/I_r)(n_s/n_r)^2$ was used to calculate the relative quantum yield of each of the sample, where $\Phi_r$ is the absolute quantum yield of the reference, $n$ is the refractive index of the solvent, $A$ is the

absorbance at the excitation wavelength, and $I$ is the integrated area under the corrected emission curve. The subscripts $s$ and $r$ refer to the sample and reference, respectively. A solution of quinine sulfate in 0.5 M $H_2SO_4$ ($\Phi_r$ = 54.6%)[16] was used as external reference.[17]

## UV-Vis spectroscopy (DCM solution).

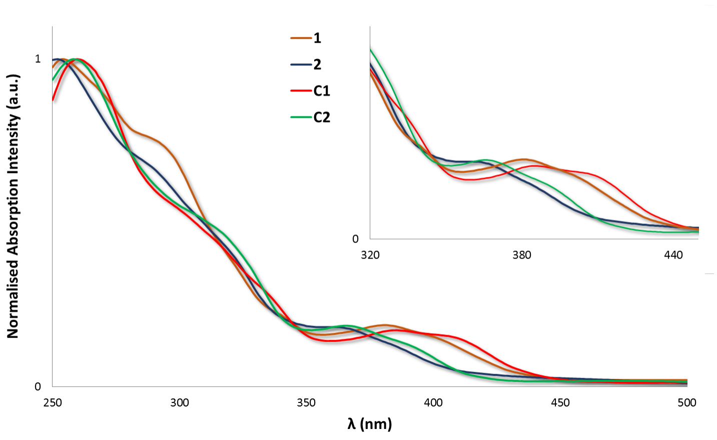


Figure **S63**. UV-Vis spectra of *rac*--[Ir(mesppy)$_2$(qpy)]$BF_4$ (**1,** in orange), *rac*--[Ir(dFmesppy)$_2$(qpy)]$PF_6$ (**2,** in blue), and cages ***rac*-C1** (in red) and ***rac*-C2** (in green) collected in $CD_2Cl_2$ at 298 K.

Table **S3**. UV-Vis absorptions of **1, 2, C1, C2**

| **Compound** | $\lambda_{max}{}^{a}$ (nm) [ε (×$10^3$ $M^{-1}$ $cm^{-1}$)]$^{b}$ |
|---|---|
| ***rac*, Λ- and Δ-1** | 255 [50.4], 294 [37.3], 381 [9.4], 403 [7.7] |
| ***rac*, Λ- and Δ-2** | 253 [51.0], 291 [33.7], 321 [18.9], 367 [8.2], 387 [5.2] |
| ***rac*, Λ- and Δ-C1** | 261 [56.8], 311 [21.5], 337 [16.2], 387 [11.3], 413 [10.8] |
| ***rac*, Λ- and Δ-C2** | 260 [58.9], 311 [26.5], 369 [15.1], 397 [9.3] |

[a] UV-Vis absorption in DCM with a concentration in the order of $10^{-5}$ – $10^{-6}$ M collected at 298 K. [b] concentration-independent extinction coefficients.

Emission spectra (DCM solution and PMMA-doped films).

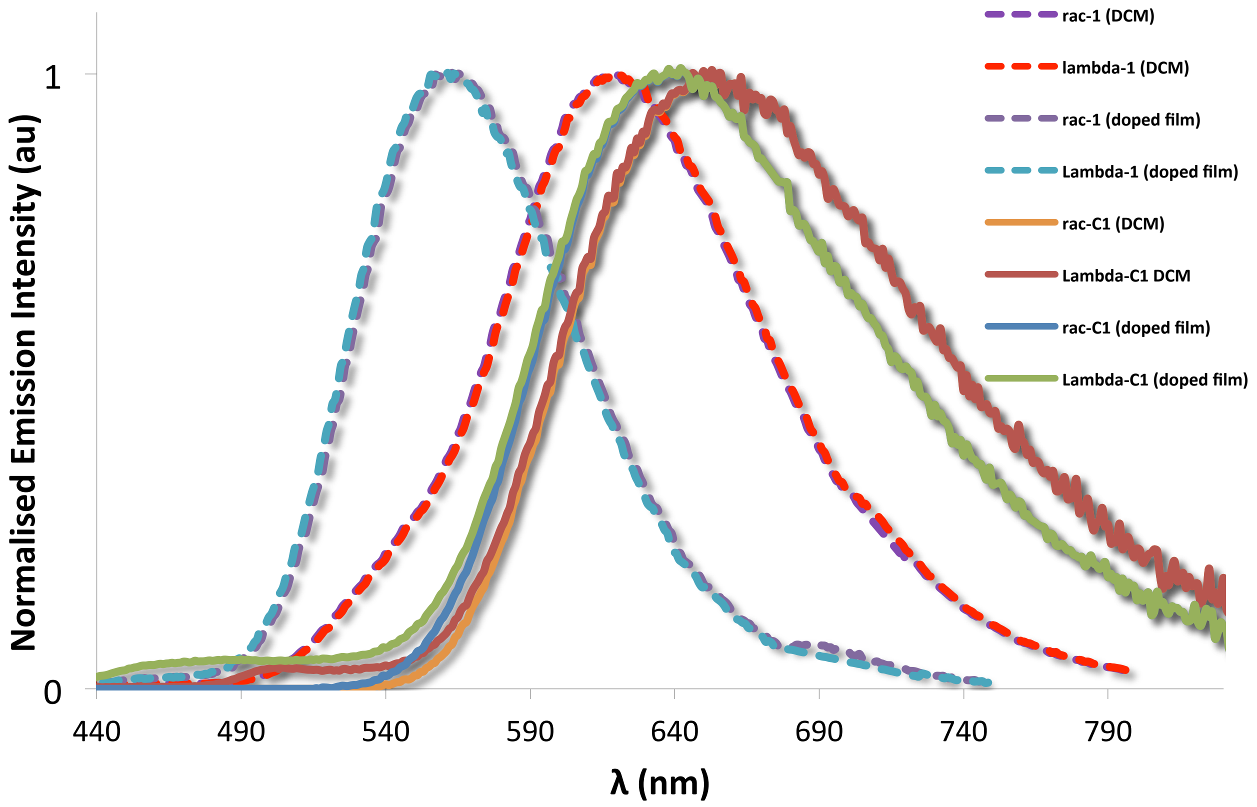


Figure **S64**. Normalized DCM emission spectra of: ***rac*-1**, dotted violet line; **Λ-1**, dotted red line; ***rac*-C1**, solid orange line; **Λ-C1**, solid red line collected in degassed DCM at 298 K and normalized emission spectra of PMMA-doped films with 5% wt % of samples spin-coated on quartz substrates of: ***rac*-1**, dotted light-violet line; **Λ-1**, dotted light-blue line; ***rac*-C1**, solid blue line; **Λ-C1**, solid green line collected at 298 K.

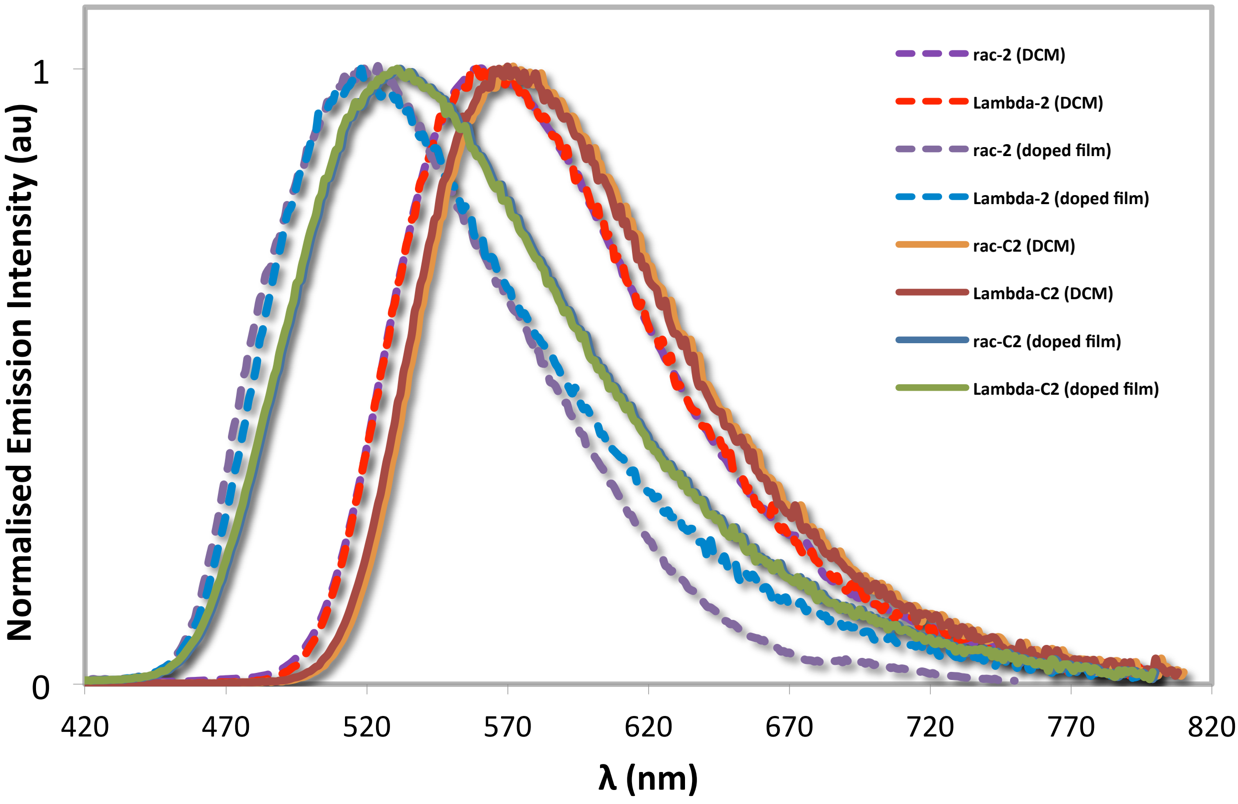


Figure **S65**. Normalized DCM emission spectra of: ***rac*-2**, dotted violet line; **Λ-2**, dotted red line; ***rac*-C2**, solid orange line; **Λ-C2**, solid red line collected in degassed DCM at 298 K and normalized emission spectra of PMMA-doped films with 5% wt % of samples spin-coated on quartz substrates of: ***rac*-2**, dotted light-violet line; **Λ-2**, dotted light-blue line; ***rac*-C2**, solid blue line; **Λ-C2**, solid green line collected at 298 K.

## Emission decays (DCM solutions and PMMA-doped films).

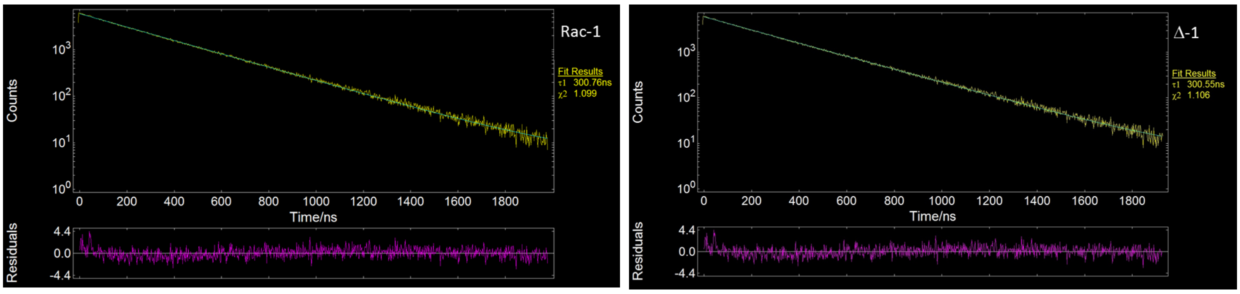


Figure **S66**. Emission decay of: ***rac*-1** (left) and **Δ-1** (right) collected in degassed DCM at 298 K ($\lambda_{exc}$ = 378 nm).

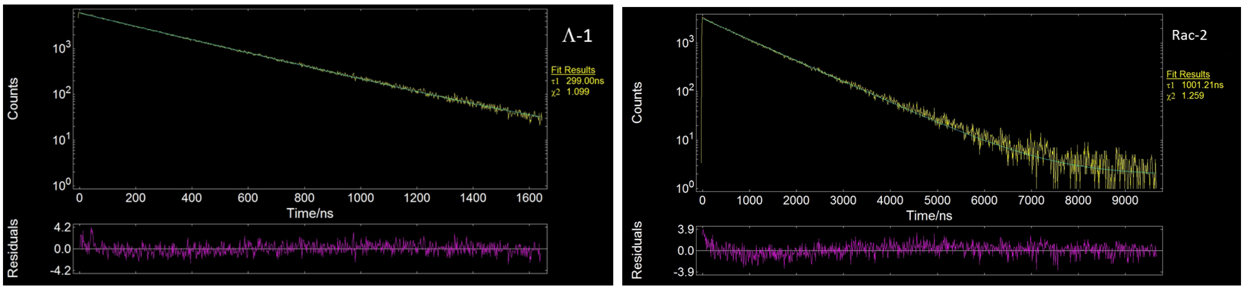


Figure **S67**. Emission decay of: **Λ-1** (left) and ***rac*-2** (right) collected in degassed DCM at 298 K ($\lambda_{exc}$ = 378 nm).

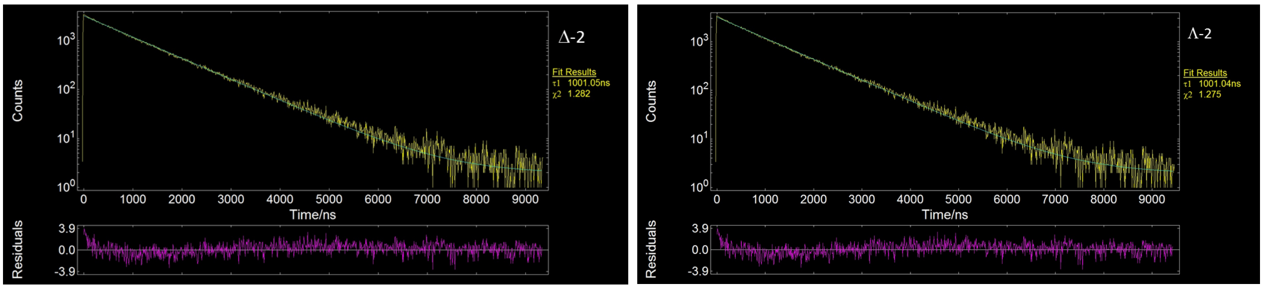


Figure **S68**. Emission decay of: **Δ-2** (left) and **Λ-2** (right) collected in degassed DCM at 298 K ($\lambda_{exc}$ = 378 nm).

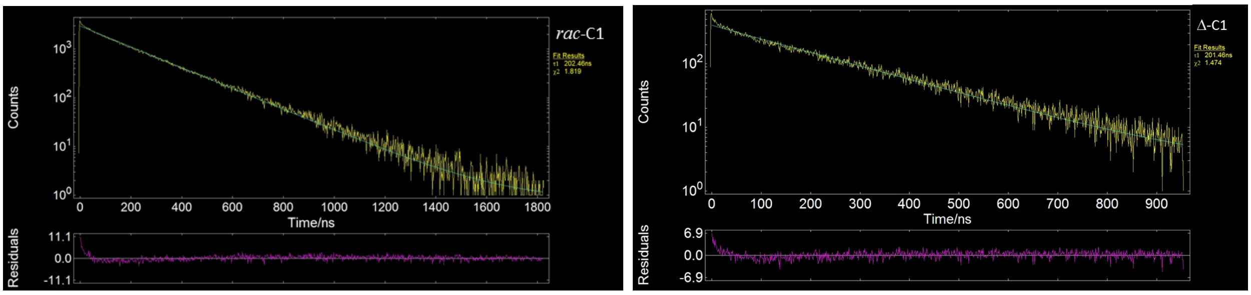


Figure **S69**. Emission decay of: ***rac*–C1** (left) and **Δ-C1** (right) collected in degassed DCM at 298 K ($\lambda_{exc}$ = 378 nm).

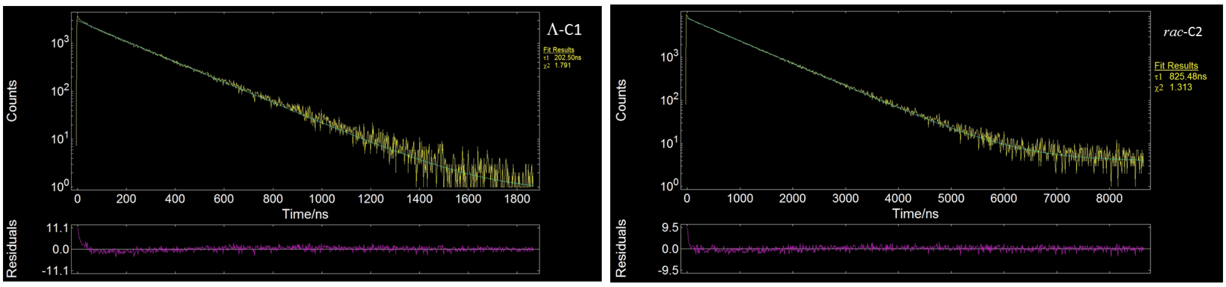


Figure **S70**. Emission decay of: **Λ-C1** (left) and ***rac*-C2** (right) collected in degassed DCM at 298 K ($\lambda_{exc}$ = 378 nm).

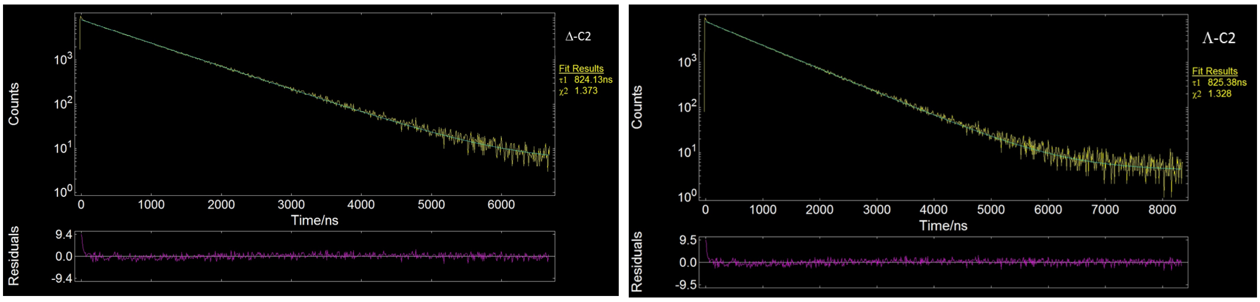


Figure **S71**. Emission decay of: **Δ-C2** (left) and **Λ-C2** (right) collected in degassed DCM at 298 K ($\lambda_{exc}$ = 378 nm).

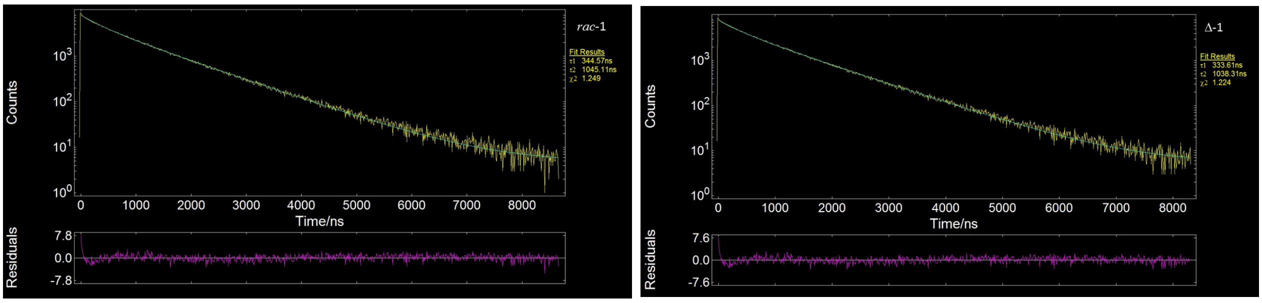


Figure **S72**. Emission decay of: ***rac*–1** (left) and **Δ-1** (right) collected in PMMA-doped films at 298 K ($\lambda_{exc}$ = 378 nm).

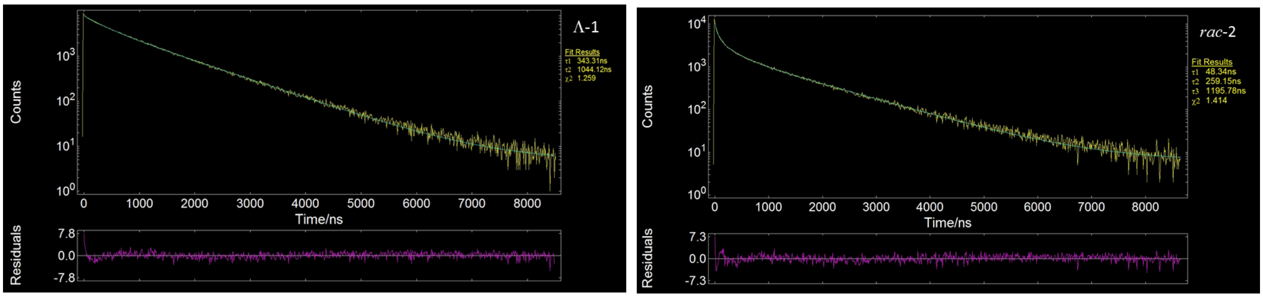


Figure **S73**. Emission decay of: **Λ−1** (left) and ***rac*−2** (right) collected in PMMA-doped films at 298 K ($\lambda_{exc}$ = 378 nm).

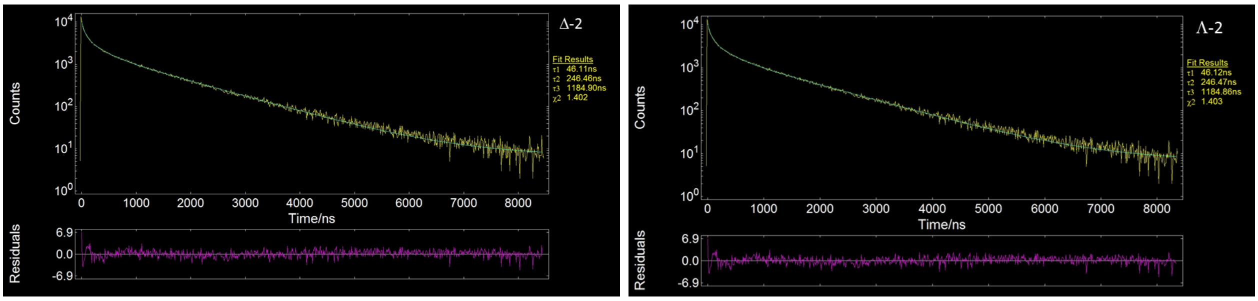


Figure **S74**. Emission decay of: **Δ−2** (left) and **Λ−2** (right) collected in PMMA-doped films at 298 K ($\lambda_{exc}$ = 378 nm).

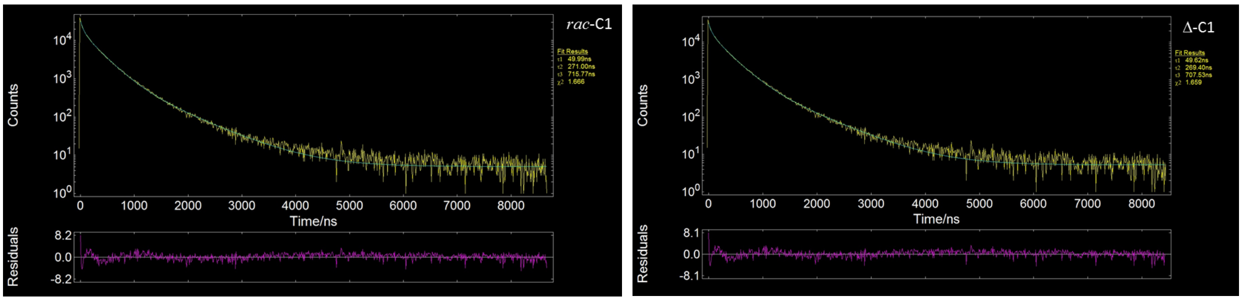


Figure **S75**. Emission decay of: ***rac*-C1** (left) and **Δ-C1** (right) collected in PMMA-doped films at 298 K ($\lambda_{exc}$ = 378 nm).

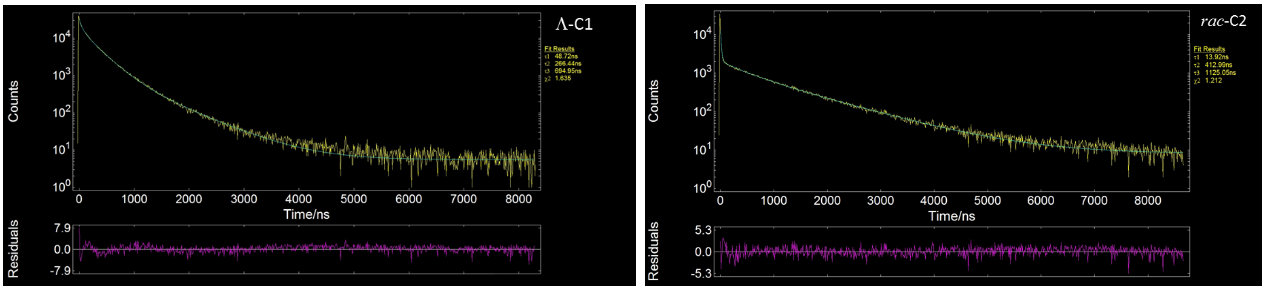


Figure **S76**. Emission decay of: **Λ-C1** (left) and ***rac*-C2** (right) collected in PMMA-doped films at 298 K ($\lambda_{exc}$ = 378 nm).

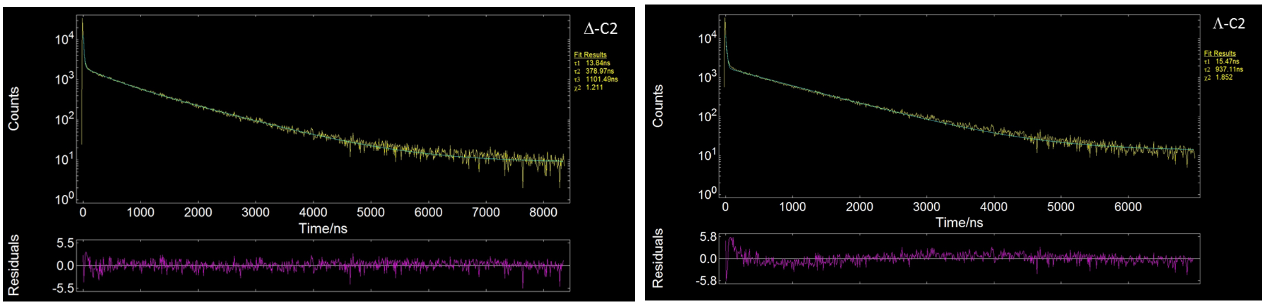


Figure **S77**. Emission decay of: **Δ-C2** (left) and **Λ-C2** (right) collected in PMMA-doped films at 298 K ($\lambda_{exc}$ = 378 nm).

# Energy Transfer studies between IrCN and Δ-C1.

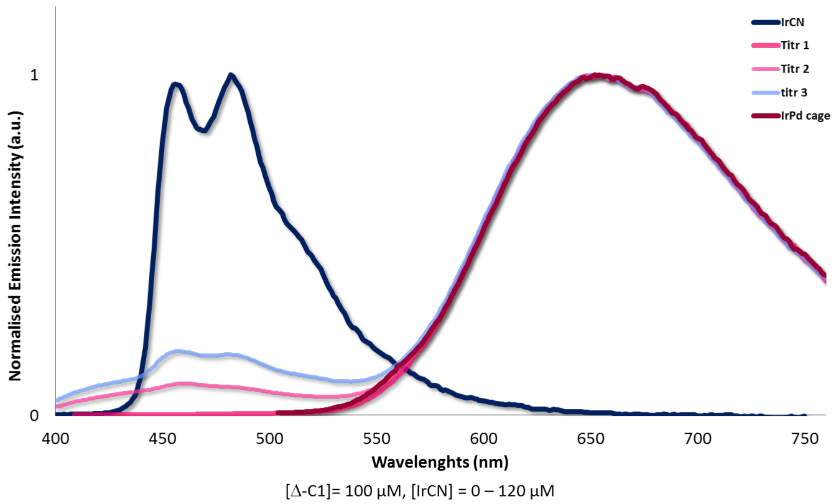


Figure **S78**. Normalized emission spectra collected in DMSO at 298 K of **IrCN** (dark-blue line), **Δ-C1** (dark red line) and of **Δ-C1** after gradual addition of **IrCN**. The concentration of **Δ-C1** was kept constant at 100 μM whereas the concentration of **IrCN** varied from 0 to 120 μM (μM of IrCN; **titr. 1** = 10, **titr. 2** = 60, **titr. 3** = 120). Due to the efficient energy transfer from **IrCN** to **Δ-C1**, the emission intensity of the red-emitting cage was found to be always predominant over the emission of the blue-emitting donor.

## Determination of binding constant for the formation of **Δ-C1⊃IrCN**

The binding constant for the host-guest structure **Δ-C1⊃IrCN** was determined from emission titration experiments (see Figure **7**). Small aliquots of **Δ-C1** were added to a 100 μM degassed solution of **IrCN** in DMSO such that the concentration of **Δ-C1** in the sample ranged from 0 μM to 120 μM. An emission spectrum of the solution was recorded after each addition and the variation of the emission intensity of **IrCN** in **Δ-C1⊃IrCN** with respect to **Δ-C1** concentration determined from this data. This data was then fitted to the sequential 1:1 binding model[18] using an iterative fitting

procedure implemented within the OpenDataFit tool of the Supramolecular program (www.supramolecular.org) (see Figure **S79**). The best fit of the binding model to the emission data afforded a value for $K_b$ of $3.9 \times 10^6 \pm 0.2$ $M^{-1}$.

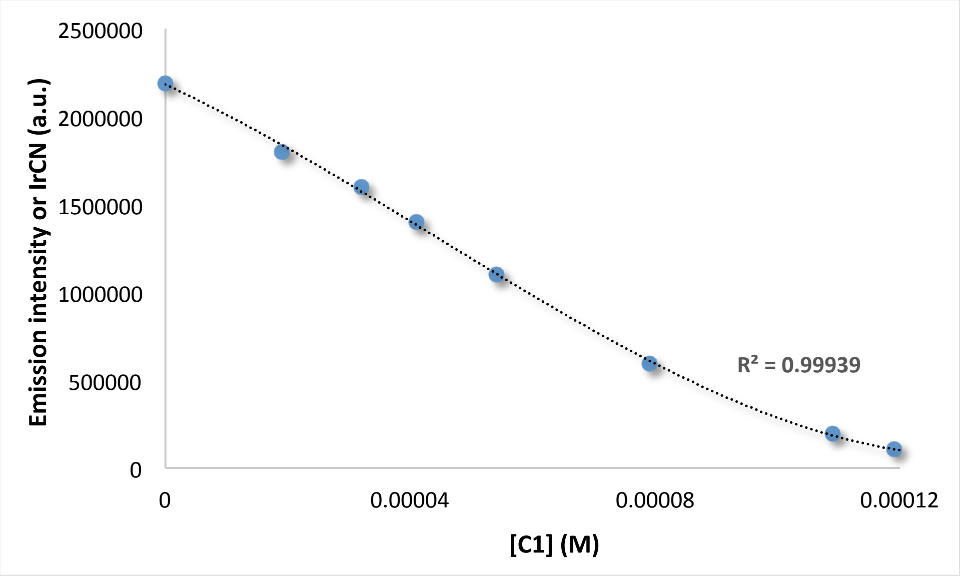


Figure **S79**. 1:1 Binding isotherm obtained by emission titration experiments of **IrCN** (donor) after gradual addition of **Δ-C1** (acceptor) in DMSO at 298 K, while monitoring the emission intensity of **IrCN** (Figure **4**) by emission spectroscopy.

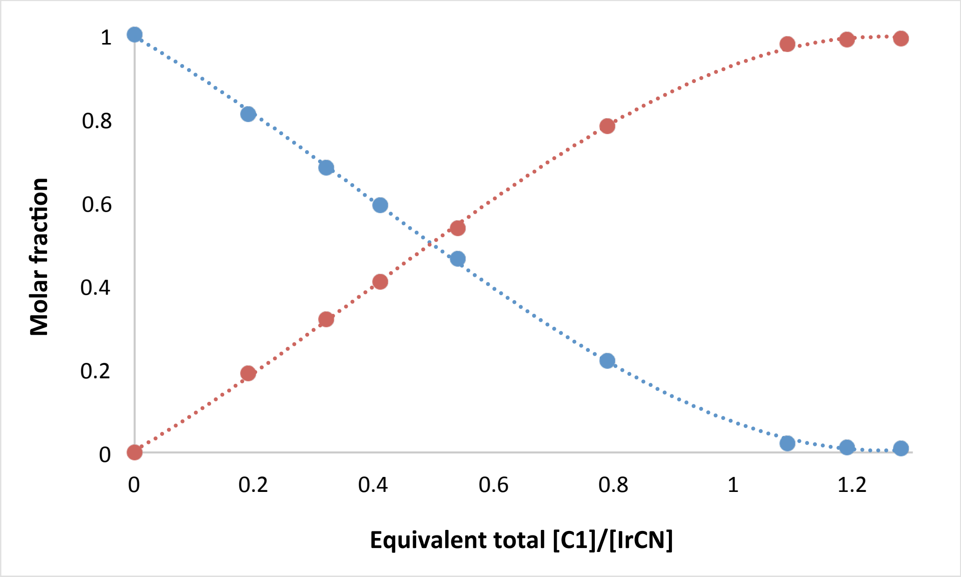


Figure **S80**. Binding model corresponding to the formation of **Δ-C1⊃IrCN**. Molar fractions of guest **Δ-C1** (in blue) and 1:1 **Δ-C1**:IrCN (in red) present in solution.

## Stern-Volmer Quenching Studies Analysis

Emission titration in DMSO at 298 K of cage **Δ-C1** (quencher) into a degassed solution of **IrCN** (donor). The concentration of **IrCN** was kept constant at 100 μM whereas the concentration of **Δ-C1** was gradually varied from 0 to 120 μM. To monitor the emission quenching of **IrCN** due to the energy transfer to the red-emitting iridium cage, we monitored the lifetime decays in correspondence of the emission maxima of **IrCN**, at $\lambda_{PL}$ 480 nm. The solutions were excited at $\lambda_{exc}$ = 378 nm.

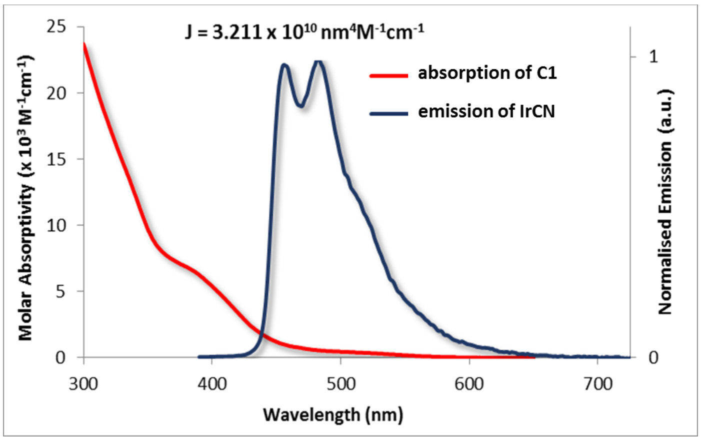


**Figure S81**. Molar absorptivity of Δ-**C1** cage in DMSO at 298 K (red line) and normalized emission of **IrCN** (blue line) collected in degassed DMSO at 298 K.

**Table S4**. Emission lifetime as a function of concentration of quencher.

| | **[IrCN]** (μM) | **[Δ-C1]** (μM) | $\tau_e$ **470 nm** (ns) |
|---|---|---|---|
| **IrCN** | 100 | - | 2915 |
| **Titr. 1** | 100 | 21 | 672 |
| **Titr. 2** | 100 | 30 | 550 |
| **Titr. 3** | 100 | 48 | 383 |

| | | | |
|---|---|---|---|
| **Titr. 4** | 100 | 101 | 181 |
| **Titr. 5** | 100 | 118 | 13 |

Emission decays monitored at 470 nm of **IrCN** and **IrCN** after gradual addition of **Δ-C1** (quencher) in degassed DMSO at 298 K. The lifetime data combined with the concentration values have been used to calculate the Stern-Volmer constants.

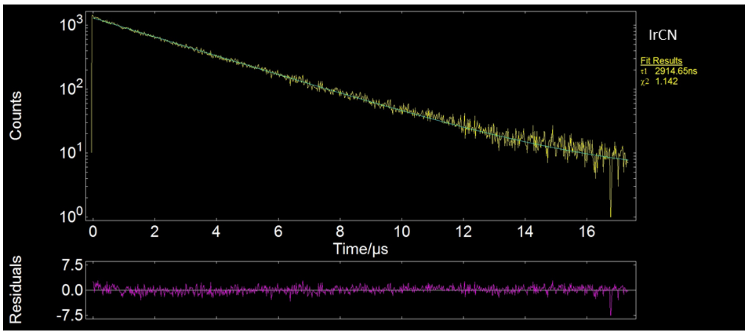


Figure **S82**. Emission decay of **TBA[Ir(dFppy)$_2$(CN)$_2$] (IrCN)** collected in degassed DMSO at 298 K ($\lambda_{exc}$ = 378 nm)**.**

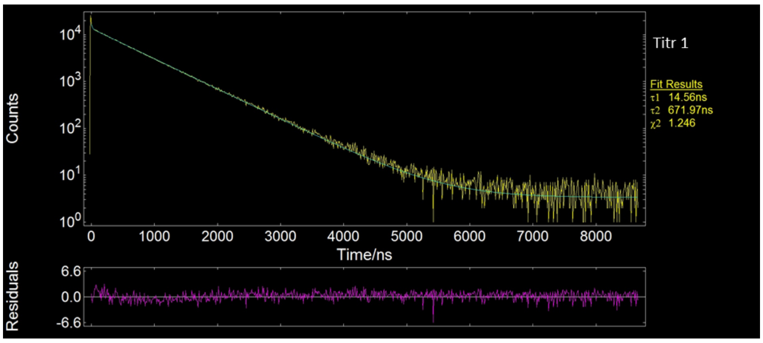


Figure **S83**. Emission decay of **TBA[Ir(dFppy)$_2$(CN)$_2$] (IrCN)** (100 μM) + **Δ-C1** (21 μM) collected in degassed DMSO at 298 K ($\lambda_{exc}$ = 378 nm).

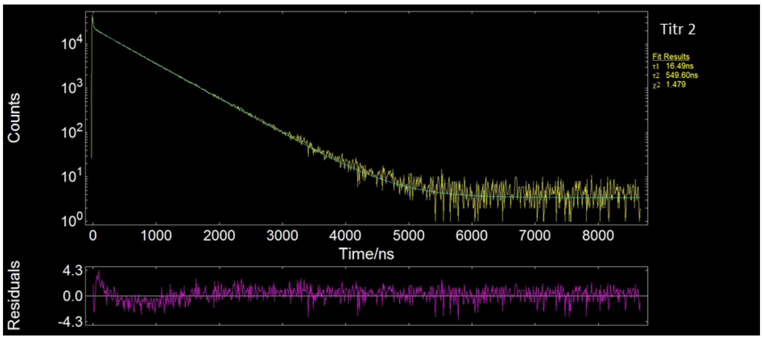

Figure **S84**. Emission decay of **TBA[Ir(dFppy)$_2$(CN)$_2$] (IrCN)** (100 μM) + **Δ-C1** (30 μM) collected in degassed DMSO at 298 K ($\lambda_{exc}$ = 378 nm).

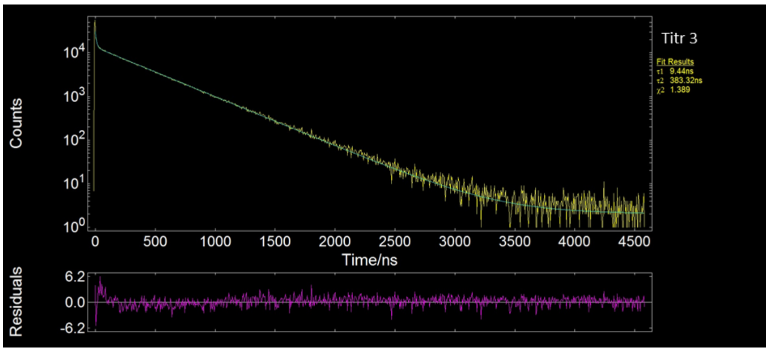


Figure **S85**. Emission decay of **TBA[Ir(dFppy)$_2$(CN)$_2$] (IrCN)** (100 μM) + **Δ-C1** (48 μM) collected in degassed DMSO at 298 K ($\lambda_{exc}$ = 378 nm).

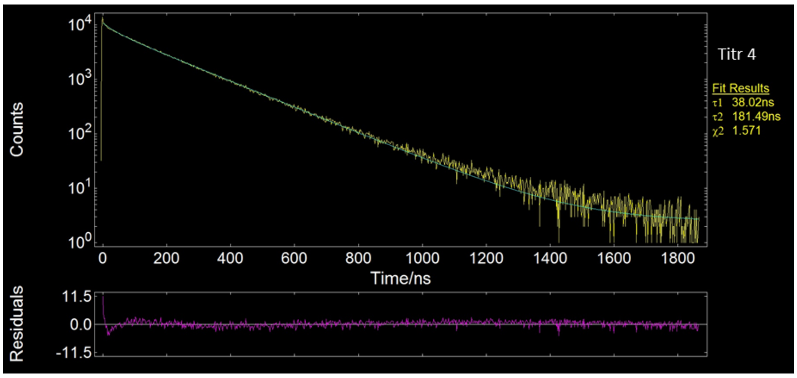


Figure **S86**. Emission decay of **TBA[Ir(dFppy)$_2$(CN)$_2$] (IrCN)** (100 μM) + **Δ-C1** (101 μM) collected in degassed DMSO at 298 K ($\lambda_{exc}$ = 378 nm).

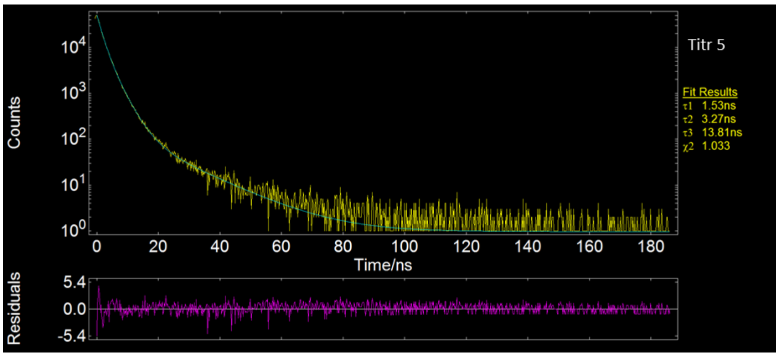


Figure **S87**. Emission decay of **TBA[Ir(dFppy)$_2$(CN)$_2$] (IrCN)** (100 μM) + **Δ-C1** (118 μM) collected degassed DMSO at 298 K ($\lambda_{exc}$ = 378 nm).

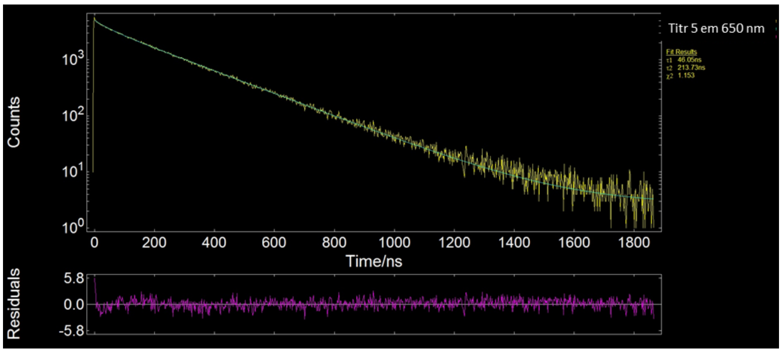


Figure **S88**. Emission decay of **TBA[Ir(dFppy)$_2$(CN)$_2$] (IrCN)** (100 μM) + **Δ-C1** (118 μM) monitored at 650 nm collected in degassed DMSO at 298 K ($\lambda_{exc}$ = 378 nm).

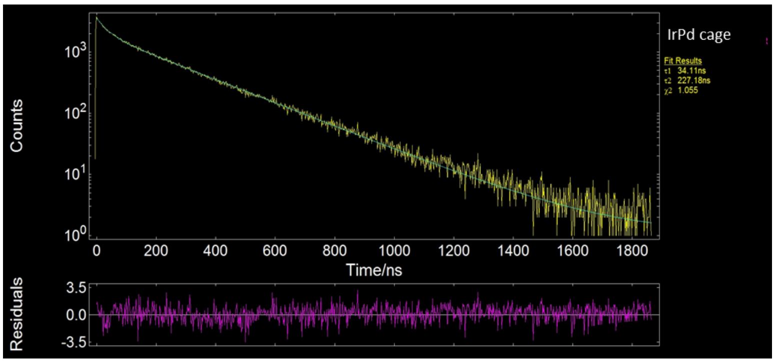


Figure **S89**. Emission decay of **Δ-C1** monitored at 650 nm collected in degassed DMSO at 298 K ($\lambda_{exc}$ = 378 nm).

# Energy Transfer Studies between Irdmbpy and Δ-C1.

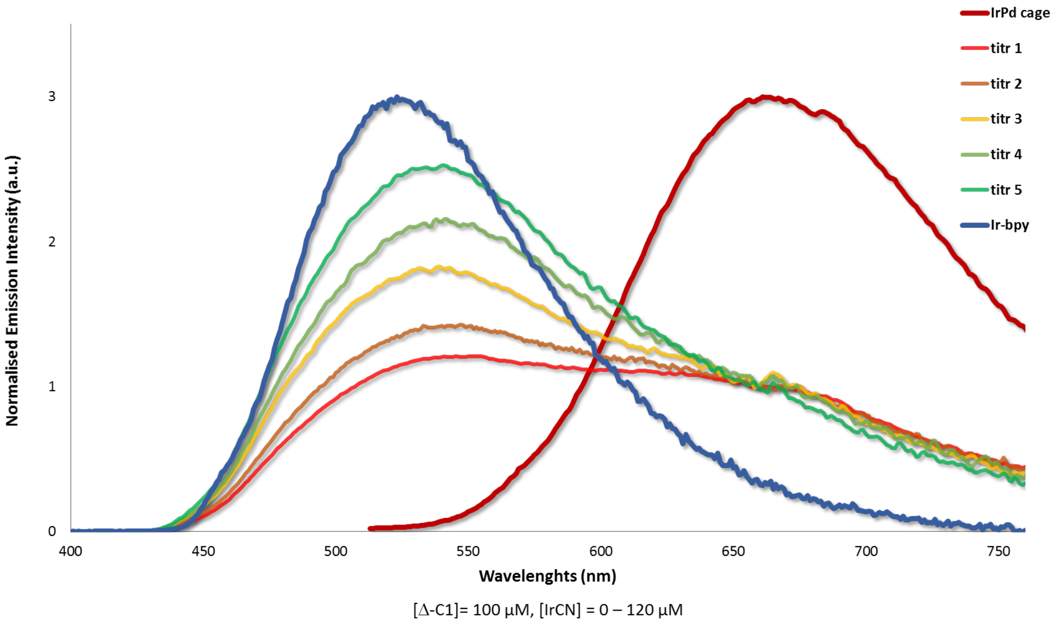


Figure **S90**. Normalized emission spectra collected in DMSO at 298 K of **Irdmbpy** (blue line), **Δ-C1** (dark red line) and of **Δ-C1** after gradual addition of **Irdmbpy**. The concentration of **Δ-C1** was kept constant at 100 μM whereas the concentration of **Irdmbpy** varied from 0 to 120 μM (μM of Irdmbpy; **titr. 1** = 10, **titr. 2** = 30, **titr. 3** = 70, **titr. 4** = 90, **titr. 5** = 120).

In contrast to that observed for the **Δ-C1⊃IrCN** assemblies, no energy transfer was observed between **Irdmbpy** and **Δ-C1**. Indeed, the emission intensity of **Irdmbpy** ($\lambda_{em}$ = 480 nm) increases proportionally with concentration. The emission intensity of **Δ-C1** is also not affected by the addition of **Irdmbpy**. The same conclusion can be extracted from the time-resolved emission experiments presented below.

**Table S5**. Lifetime decays.

| | **[Irdmbpy]** (μM) | **[Δ-C1]** (μM) | $\tau_e$ **470 nm** (ns) |
|---|---|---|---|
| **Irdmbpy** | 100 | - | 1167 |
| **Titr. 5** | 100 | 118 | 950 |

Emission decays monitored at 520 nm of **Irdmbpy** and **Irdmbpy** after gradual addition of **Δ-C1** (quencher) in degassed DMSO at 298 K.

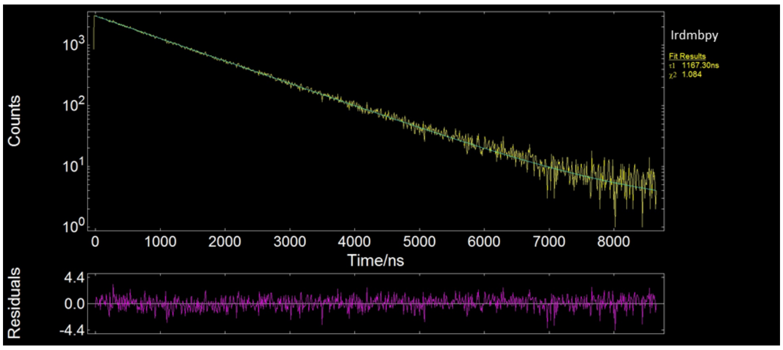


Figure **S91**. Emission decay of **[Ir(dFppy)$_2$(dmbpy)]PF$_6$ (Irdmbpy)** monitored at 530 nm collected in degassed DMSO at 298 K ($\lambda_{ex}$ = 378 nm).

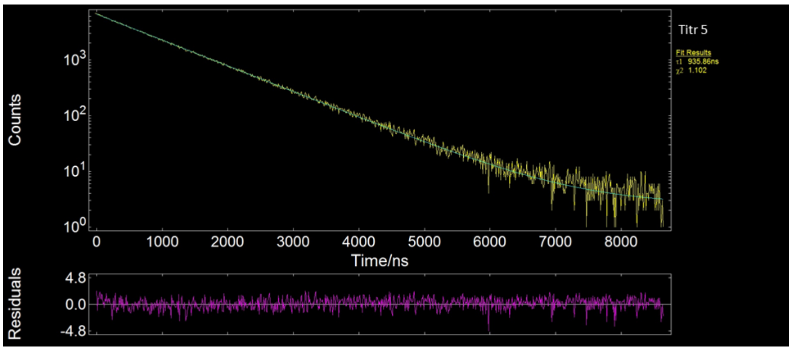


Figure **S92**. Emission decay of **[Ir(dFppy)$_2$(dmbpy)$_2$] (Irdmbpy)** (100 μM) + **Δ-C1** (118 μM) monitored at 530 nm collected in degassed DMSO at 298 K ($\lambda_{ex}$ = 378 nm).

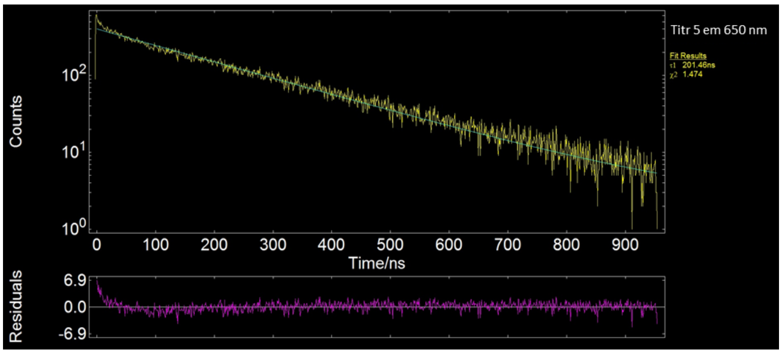


Figure **S93**. Emission decay of **[Ir(dFppy)$_2$(dmbpy)$_2$] (Irdmbpy)** (100 μM) + **Δ-C1** (118 μM) monitored at 650 nm collected in degassed DMSO at 298 K ($\lambda_{ex}$ = 378 nm).

# Computational details

All calculations are based on Hartree Fock theory (HF) theory. The geometries of the host and host-guest complexes were fully optimized without symmetry restriction in the gas phase using the 6-31G(d) basis set for the light atoms. Scalar relativistic effects were included for the Ir and Pd atoms by using the LANL2DZ pseudopotentials.[19] All calculations were carried out with the Gaussian09.D01 program package.[20]

# References

(1). D. Rota Martir, A. K. Bansal, V. Di Mascio, D. B. Cordes, A. F. Henwood, A. M. Z. Slawin, P. C. J. Kamer, L. Martinez-Sarti, A. Pertegas, H. J. Bolink, I. D. W. Samuel and E. Zysman-Colman, *Inorganic Chemistry Frontiers*, 2016, **3**, 218-235.
(2). D. Rota Martir, G. J. Hedley, D. B. Cordes, A. M. Z. Slawin, D. Escudero, D. Jacquemin, T. Kosikova, D. Philp, D. M. Dawson, S. E. Ashbrook, I. D. W. Samuel and E. Zysman-Colman, *Dalton Trans.*, 2016, **45**, 17195-17205.
(3). D. R. Martir, C. Momblona, A. Pertegás, D. B. Cordes, A. M. Z. Slawin, H. J. Bolink and E. Zysman-Colman, *ACS Applied Materials & Interfaces*, 2016, **8**, 33907-33915.
(4). M. Nonoyama, *Bull. Chem. Soc. Jpn.*, 1974, **47**, 767-768.
(5). M. Mauro, K. C. Schuermann, R. Prétôt, A. Hafner, P. Mercandelli, A. Sironi and L. De Cola, *Angew. Chem. Int. Ed.*, 2010, **49**, 1222-1226.
(6). K. P. S. Zanoni, B. K. Kariyazaki, A. Ito, M. K. Brennaman, T. J. Meyer and N. Y. Murakami Iha, *Inorg. Chem.*, 2014, **53**, 4089-4099.
(7). *CrystalClear-SM Expert v. 2.1;* , Rigaku Americas, Rigaku Americas, The Woodlands, Texas, USA and Rigaku Corporation, Tokyo, Japan, 2010-2014.

(8). *CrysAlisPro v1.171.38.43i.*, Rigaku Oxford Diffraction, Rigaku Corporation, 2015.
(9). P. T. Beurskens, G. Beurskens, R. de Gelder, S. Garcia-Granda, R. O. Gould, R. Israel and J. M. M. Smits, Crystallography Laboratory, University of Nijmegen, The Netherlands, , 1999.
(10). G. Sheldrick, *Acta Crystallogr., Sect. C: Cryst. Struct. Commun.*, 2015, **71**, 3-8.
(11). *CrystalStructure v4.2.*, The Woodlands, Texas, USA and Rigaku Corporation, Tokyo, Japan, 2014.
(12). H. Nowell, S. A. Barnett, K. E. Christensen, S. J. Teat and D. R. Allan, *Journal of Synchrotron Radiation*, 2012, **19**, 435-441.
(13). L. Palatinus and G. Chapuis, *J. Appl. Crystallogr.*, 2007, **40**, 786-790.
(14). L. Farrugia, *J. Appl. Crystallogr.*, 1999, **32**, 837-838.
(15). G. A. Crosby and J. N. Demas, *J. Phys. Chem.*, 1971, **75**, 991-1024.
(16). W. H. Melhuish, *J. Phys. Chem.*, 1961, **65**, 229-235.
(17). A. M. Brouwer, *Pure Appl. Chem.*, 2011, **83**, 2213-2228.
(18). P. Thordarson, *Chem. Soc. Rev.*, 2011, **40**, 1305-1323.
(19). (a) P. J. Hay and W. R. Wadt, *J Chem. Phys.*, 1985, **82**, 299-310; (b) L. E. Roy, P. J. Hay and R. L. Martin, *J. Chem. Theory Comput.*, 2008, **4**, 1029-1031; (c) A. W. Ehlers, M. Böhme, S. Dapprich, A. Gobbi, A. Höllwarth, V. Jonas, K. F. Köhler, R. Stegmann, A. Veldkamp and G. Frenking, *Chem. Phys. Lett.*, 1993, **208**, 111-114.
(20). M. J. Frisch, G. W. Trucks, H. B. Schlegel, G. E. Scuseria, M. A. Robb, J. R. Cheeseman, G. Scalmani, V. Barone, B. Mennucci, G. A. Petersson, H. Nakatsuji, M. Caricato, X. Li, H. P. Hratchian, A. F. Izmaylov, J. Bloino, G. Zheng, J. L. Sonnenberg, M. Hada, M. Ehara, K. Toyota, R. Fukuda, J. Hasegawa, M. Ishida, T. Nakajima, Y. Honda, O. Kitao, H. Nakai, T. Vreven, J. A. Montgomery Jr., J. E. Peralta, F. Ogliaro, M. Bearpark, J. J. Heyd, E. Brothers, K. N. Kudin, V. N. Staroverov, R. Kobayashi, J. Normand, K. Raghavachari, A. Rendell, J. C. Burant, S. S. Iyengar, J. Tomasi, M. Cossi, N. Rega, J. M. Millam, M. Klene, J. E. Knox, J. B. Cross, V. Bakken, C. Adamo, J. Jaramillo, R. Gomperts, R. E. Stratmann, O. Yazyev, A. J. Austin, R. Cammi, C. Pomelli, J. W. Ochterski, R. L. Martin, K. Morokuma, V. G. Zakrzewski, G. A. Voth, P. Salvador, J. J. Dannenberg, S. Dapprich, A. D. Daniels, Ö. Farkas, J. B. Foresman, J. V. Ortiz, J. Cioslowski and D. J. Fox, Gaussian Inc., Wallingford, CT, 2009.
